# The MetabolomeExpress Project: enabling web-based processing, analysis and transparent dissemination of GC/MS metabolomics datasets

**DOI:** 10.1186/1471-2105-11-376

**Published:** 2010-07-14

**Authors:** Adam J Carroll, Murray R Badger, A Harvey Millar

**Affiliations:** 1 Australian Research Council Centre of Excellence in Plant Energy Biology, University of Western Australia, Perth, Western Australia, Australia; 2 Australian Research Council Centre of Excellence in Plant Energy Biology; 3 Research School of Biology, The Australian National University, Acton, Australian Capital Territory, Australia

## Abstract

**Background:**

Standardization of analytical approaches and reporting methods via community-wide collaboration can work synergistically with web-tool development to result in rapid community-driven expansion of online data repositories suitable for data mining and meta-analysis. In metabolomics, the inter-laboratory reproducibility of gas-chromatography/mass-spectrometry (GC/MS) makes it an obvious target for such development. While a number of web-tools offer access to datasets and/or tools for raw data processing and statistical analysis, none of these systems are currently set up to act as a public repository by easily accepting, processing and presenting publicly submitted GC/MS metabolomics datasets for public re-analysis.

**Description:**

Here, we present MetabolomeExpress, a new File Transfer Protocol (FTP) server and web-tool for the online storage, processing, visualisation and statistical re-analysis of publicly submitted GC/MS metabolomics datasets. Users may search a quality-controlled database of metabolite response statistics from publicly submitted datasets by a number of parameters (eg. metabolite, species, organ/biofluid etc.). Users may also perform meta-analysis comparisons of multiple independent experiments or re-analyse public primary datasets via user-friendly tools for t-test, principal components analysis, hierarchical cluster analysis and correlation analysis. They may interact with chromatograms, mass spectra and peak detection results via an integrated raw data viewer. Researchers who register for a free account may upload (via FTP) their own data to the server for online processing via a novel raw data processing pipeline.

**Conclusions:**

MetabolomeExpress https://www.metabolome-express.org provides a new opportunity for the general metabolomics community to transparently present online the raw and processed GC/MS data underlying their metabolomics publications. Transparent sharing of these data will allow researchers to assess data quality and draw their own insights from published metabolomics datasets.

## Background

The rapidly growing field of metabolomics aims to monitor the levels of as many metabolites as possible in living systems as they respond to genetic and/or environmental perturbations. A variety of analytical technologies have been applied to metabolomics studies. However, GC/MS is by far the most commonly employed technology. Reasons for this include its relative affordability, sensitivity and reproducibility. GC/MS is therefore a particularly attractive target platform for development of web-based tools to support community-scale comparisons and mining of raw and processed metabolomics data.

Before biological insights derived from metabolomics-based studies can be communicated, they must first be extracted from raw instrumental datasets [1]. Raw GC/MS metabolomic datasets are typically large and complex in nature, frequently comprised of tens to hundreds of data files - each containing convoluted signals for hundreds to thousands of analytes. Sophisticated algorithms are therefore required to identify and quantify signals corresponding to biologically relevant analytes and obtain a quantitative description of the metabolic effect(s) associated with the experimental factor(s) of interest [1]. This data extraction process may be broken down into a number of general steps:

1. Detection of analytically useful signal features (ie. 'peaks')

2. Identification of biologically relevant signal features by matching against a reference library of known signal characteristics for biological analytes.

3. Assignment of some quantitative signal measurement to each identified biological analyte.

4. Construction of a [Metabolite x Sample] data matrix, usually with some form of data normalization against internal standard(s) and/or biological sample mass/volume/amount.

5. Use of statistical and exploratory data analysis tools to determine the effect(s) of experimental factors on metabolite levels.

6. Interpretation of observed metabolite-level changes in the context of prior knowledge about the metabolic system under investigation (eg. by visual mapping of observed metabolite level changes onto metabolic pathway diagrams).

A general lack of software for carrying out the above data analysis steps quickly and easily has been one of the greatest challenges hampering the establishment of metabolomics as a mainstream technique. This challenge has triggered widespread efforts to develop data processing software tailored to the needs of metabolomics researchers, resulting in a variety of specialised open-source and proprietary tools now being available. In most cases, these packages do not perform every step of the metabolomics data processing workflow and therefore require transfer of data to and from third party software. For example, some tools such as AMDIS [2], XCMS and XCMS2 [3,4], MetaQuant [5], MetAlign [6], MetaboliteDetector [7], MSFACTs [8], MET-IDEA [9] and TagFinder [10] focus on raw data pre-processing, peak detection and/or quantification but provide relatively little functionality to aid statistical analysis or biological interpretation. On the other hand, other tools such as MetaFIND [11], TICL [12], MetaboAnalyst [13] and MeltDB [14] provide relatively little or no raw signal processing functionality of their own but focus more on biological interpretation via multivariate explorative- and statistical-analysis of pre-processed datasets produced by third-party software packages.

It has been demonstrated in the microarray field that standardization of analytical approaches and reporting methods via community-wide collaboration and enforcement by scientific journals can work synergistically with web-tool development to result in rapid community-driven expansion of online data repositories suitable for data mining and meta-analysis. While GC/MS provides the reproducibility required to support a similar community effort in metabolomics, there have only been a few reports on systems designed to undertake this (eg. the SetupX database [15] and the Golm Metabolome Database (GMD, [16]) but each have limitations, for example, requiring propriety software for data processing or limited opportunities for public submissions.

Here, we introduce MetabolomeExpress, a new web-tool for the online storage, processing, visualization and re-analysis of publicly-submitted raw and processed GC/MS metabolomics datasets. The MetabolomeExpress web-server application https://www.metabolome-express.org was designed to perform three main functions. The first was to centrally store complete GC/MS metabolomics datasets (including raw data, processed data, experimental metadata and GC/MS reference libraries) in a way that makes them highly accessible and useful to the public without requiring them to download and locally reprocess the enormous amounts of data (frequently > > 1 GB) that constitute the typical GC/MS metabolomics dataset. The second function was to provide researchers with cost-free online access to a powerful raw data processing pipeline that extracts biological information from raw GC/MS data with minimal effort. The third function was to store metabolite response statistics in a central database that provides tools enabling the searching, comparison and verification of results. Registration is free for non-commercial use.

## Construction and content

### Structural overview

**Figure 1:**
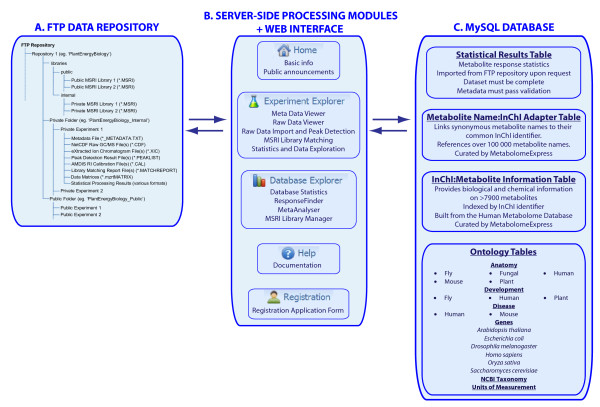
**Structural overview of the MetabolomeExpress webserver**. MetabolomeExpress consists of four interacting layers: i) an FTP repository; ii) an SQL database; iii) a set of server-side data-processing modules; and iv) a web-interface. The contents and general structure of each layer are indicated schematically. Further details are provided in the main text.

In a structural sense, MetabolomeExpress is comprised of four interacting layers. The first is an FTP-accessible repository system on the server file system that stores raw and processed GC/MS datasets. The second layer is a simple MySQL database containing three core tables: i) a table of highly annotated pre-processed metabolite response statistics with references to underlying data including raw data signals contained in files in the file system; ii) a table linking ~100,000 commonly used metabolite names to their unique InChI structural identifier strings; and iii) a table linking InChI-encoded chemical structures to a variety of biochemical, bioinformatic and physicochemical attributes. Most of the information in the latter two database tables was obtained by parsing the file 'metabocards.txt' http://www.hmdb.ca/public/downloads/current/metabocards.zip representing Version 2.5 of the publicly available Human Metabolome Database [17]. A number of missing plant metabolites have been added since and curation of the metabolite information tables will be an ongoing process. The MySQL database also contains a variety of tables storing different ontologies and controlled vocabulary terms. The third layer of MetabolomeExpress consists of a set of novel server-side data processing modules implemented in PHP http://www.php.net and R http://www.r-project.org. The fourth structural layer is a JavaScript-based web interface that provides integrated access to all the data-processing, -visualisation and -analysis tools. See Figure 1 for a structural overview. Usage instructions and finer structural details are presented in the MetabolomeExpress Users Manual (additional file 1 with updates available from the MetabolomeExpress website) while a detailed commentary on the FTP repository system, data and metadata formats and controlled vocabularies can be found in additional file 2.

### The MetabolomeExpress database of metabolite response statistics

One of the central purposes of a metabolomics database is to store treatment:control metabolite signal intensity ratio statistics in a central database and provide tools to: i) search for metabolic phenotypes of interest; ii) compare the results of different experiments; and iii) manually verify original interpretations of raw data. For this purpose, MetabolomeExpress uses a simple database table containing columns for statistical information and a wide variety of administrative, biological and technical metadata. The database currently contains > 9500 metabolite ratio statistics derived from 11 experiments published in 8 articles in high-ranking plant science journals [18-25] and all future datasets published by our group will be disseminated in this way. We also plan to proactively gather and incorporate metabolite response data from the literature into the database.

### Quality control of datasets submitted to the MetabolomeExpress database of metabolite response statistics

Any user with a complete dataset stored in a MetabolomeExpress FTP repository may submit this dataset to be imported into the main statistical database. The quality control model used by MetabolomeExpress follows essentially the same principles as the major microarray data repositories. There is no requirement for data to be processed with the MetabolomeExpress data processing pipeline, as long as all the required data is provided in the correct formats. The MetabolomeExpress team does not make any subjective assessment of the quality of data or the scientific merit of a submitted experiment, nor does the data need to be published in a peer-reviewed journal. Rather, quality control is totally objective (carried out automatically by a computer script) and serves only to ensure that the dataset provided is complete (ie. it includes: a correctly completed metadata file, all raw data files, peak lists, a library match report, a normalised data matrix and a statistical results file - all formatted correctly). The validation script uses human-readable 'validation template' files defining reporting requirements and controlled vocabularies for major metabolomics research areas (eg. plant, animal, bacterial, fungal and environmental) and model systems with highly-developed bioinformatics resources (eg. *Arabidopsis thaliana*, rice, human, mouse, *Escherichia coli *and *Saccharomyces cerevisiae*). These templates have been designed to facilitate reporting according to recommendations of the Metabolomics Standards Initiative (MSI; http://msi-workgroups.sourceforge.net/). Currently implemented templates are available from the MetabolomeExpress website while instructions for their interpretation are provided in the MetabolomeExpress User's Guide (additional file 1). If a submitted dataset is cleared by the validation script, a final security check is made by the MetabolomeExpress curator before results are imported into the database.

## Utility

### Overview of the MetabolomeExpress web interface

The MetabolomeExpress web interface provides two main tools - *Experiment Explorer *and *Database Explorer*. *Experiment Explorer *is used to process and analyse raw and processed experimental datasets located in user FTP repositories while *Database Explorer *is used to interact with the contents of the metabolite response statistics database and visualise the contents of shared GC/MS reference libraries. A navigation tree allows contents of the FTP repositories to be browsed, downloaded or loaded into *Experiment Explorer*. See Figure 1 for an overview of the web-interface.

### The raw data viewer

One of the distinguishing features of MetabolomeExpress compared to other available GC/MS metabolomics web-tools is its *Raw Data Viewer *tool (a component of *Experiment Explorer*). A key goal of the project was to provide raw data interaction capability comparable to that offered by modern desktop chromatogram analysis programs. The current version of the viewer has two windows: one for displaying chromatograms and one for displaying arbitrary mass-spectral scans. One or more chromatograms may be simultaneously overlaid in the viewer and two 'colour-channels' are available so that two sets of chromatograms may be compared. Peak detection and/or library matching results may also be overlaid on chromatograms.

To our knowledge, MeltDB [14] is the only other web application currently featuring online GC/MS chromatogram visualisation. The MetabolomeExpress raw data viewer offers a number of advantages over the MeltDB chromatogram view. These include abilities to: i) visualise arbitrary MS scans and extracted ion chromatograms (EICs) as opposed to only pre-processed spectra and total ion chromatograms (TICs); ii) zoom in and out on chromatograms to visualise fine chromatographic details; iii) overlay multiple chromatograms in a conventional side-on line graph format. Another unique feature of our viewer is a chromatographic statistical scan tool that highlights retention time regions (MS scans) that show a statistically significant difference in signal intensity (and an intensity ratio greater than a user-specified threshold) between the two chromatogram sets loaded into the two colour channels. This is useful for rapidly locating peaks corresponding to differentially expressed metabolites independently of peak detection and library matching.

### Overview of the MetabolomeExpress data processing pipeline

**Figure 2:**
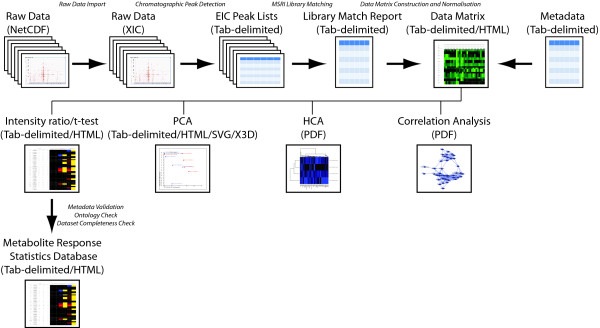
**Overview of the MetabolomeExpress data processing pipeline**. Data flows through the MetabolomeExpress pipeline are illustrated schematically as a series of processing steps. File formats generated at each processing stage are indicated in brackets. Further details are provided in the main text.

As a GC/MS data processing and analysis pipeline, MetabolomeExpress provides tools for chromatographic peak detection, peak identification (library matching), data matrix construction and a range of statistical and multivariate analysis tools including t-test, principal components analysis (PCA) and hierarchical clustering analysis (HCA). The basic mechanics and validation of each of these tools is described briefly in the sections below while detailed algorithm descriptions and a demonstration of the use of each tool are provided in the MetabolomeExpress User's Guide (additional file 1). See Figure 2 for a flow diagram of the data processing pipeline.

### Chromatographic peak detection

The aim of chromatographic peak detection is to capture useful information about analytically important instrument signals (ie. chromatographic peaks) while discarding signals devoid of useful analytical information (such as baseline noise). For this purpose, MetabolomeExpress uses a simple yet highly effective slope-based peak detection algorithm (*PeakFinder*) to detect chromatographic peaks in all extracted ion chromatograms and generate, for each raw data file, a corresponding tab-delimited peak list report file. The algorithm may be passed a number of settings that tailor the algorithm to suit particular chromatographic and mass spectral conditions. These include: i) a minimum 'slope' threshold that a signal must exceed in order to be considered as possibly being part of a peak; ii) minimum peak height; iii) minimum peak width; iv) minimum peak area; and v) a minimum peak purity factor (defined as the proportion of total peak area that lies above its lowest point). In our experience, once the optimal settings for a given type of data have been set, no further adjustment is required as long as no major changes to mass spectral or chromatographic protocols have been made. A detailed description of the algorithm's mechanics is presented in the MetabolomeExpress User's Guide (additional file 1).

To validate the *PeakFinder *algorithm, we first empirically determined the optimal parameter settings for detecting as many real chromatographic peaks as possible while minimising the number of false peak detections where no obvious chromatographic peak was present. We then compared the output of *PeakFinder *(additional file 3) with that of the widely used proprietary software package, ChemStation (Agilent Technologies, additional file 3), under default settings. To do this, we processed the same chromatogram (the m/z = 73 extracted ion chromatogram from the analysis of a complex metabolite standard mixture, additional file 3) with each algorithm and manually aligned peak detection results corresponding to the same peaks. *PeakFinder *detected a total of 129 peaks, which included 74 (96%) of the 77 EIC peaks detected by the ChemStation algorithm plus an additional 55 peaks not reported by ChemStation (additional file 3). Visual examination of peak detection results overlaid on the chromatogram revealed that all the peaks detected by ChemStation and 115 (90%) of the peaks detected by *PeakFinder *were clear and valid peak integrations while, of the 129 peaks detected by *PeakFinder*, 7 (5%) were partial but otherwise valid integrations of very small and noisy peaks, 4 (3%) were detections of very small signal features that were difficult to discern from baseline noise and 3 (2%) were clear false positive detections in regions of baseline noise. Increasing the minimum peak purity factor from 2 to 6 removed all but one false-integration. However, this increased stringency came at the expense of 33 valid integrations. Importantly, *PeakFinder *still outperformed ChemStation by 11 valid integrations under these settings.

Given that MetabolomeExpress library matching (subsequent to EIC peak detection) requires both correct retention index and multiple ions with correct intensity ratios and that analyte quantification is based on carefully selected specific quantifier ions (which are likely to be relatively abundant in positively matched spectra), it is highly unlikely that the occasional false integration of near-baseline signal features will end up affecting final results. We therefore recommend accepting the above 'false-positive' EIC integration rate of ≤ 10% for the sake of gaining a 40% increase in the number of 'true-positive' integrations. To further validate *PeakFinder*, we performed linear regressions to determine the correlations (R^2 ^values) between *PeakFinder- *and ChemStation-reported retention times and peak areas. Regression of retention times yielded an R^2 ^of 1.0000 while regression of integrated peak areas gave an R^2 ^of 0.997 indicating excellent correlation between the two algorithms. Detailed tables of integration results and regression analyses are presented in additional file 3.

### Mass-spectral and retention-index (MSRI) library matching and quantification

Any detailed biological interpretation of a GC/MS metabolomics data set requires that signals corresponding to analytes of biological origin are correctly identified and distinguished from those corresponding to artefacts or internal standard compounds. Compliance with Metabolomics Standards Initiative (MSI) standards for metabolite identification requires that at least two orthogonal analytical parameters are used to match analytical signals to particular metabolites [26]. For this purpose, MetabolomeExpress uses two widely-accepted identification parameters: retention index (RI) and mass spectrum.

The MetabolomeExpress MSRI library matching algorithm accepts EIC peak lists (as generated by the *PeakFinder *algorithm), a mass-spectral and retention index (MSRI) GC/MS reference library (a table of retention indices, mass spectra and quantifier ion information for authentic standards and unknown analytes observed in biological samples) and a simple RI calibration file (a table of retention times and retention indices for an array of RI standards such as n-alkanes) as input and generates a match report file as output. A number of matching criteria may be set.

The library matching process employed by the algorithm is detailed in the MetabolomeExpress User's Guide (additional file 1). Briefly, EIC peaks are grouped (on the basis of their apex RIs) into 0.1 RI unit bins which may then be considered as 'preliminary spectra'. A library search is then performed on each RI bin. Each library search begins by searching the supplied MSRI library for entries with RIs within a specified RI tolerance window of the query bin's RI. If a library entry with an RI match is thus identified, the bin spectrum is searched for each analyte-specific quantifier ion specified for in the library entry. If no such quantifier ion is found, the match attempt is aborted. However, if a quantifier ion is found, the intensity of that ion and the library spectrum are used to predict the expected intensities of qualifier ions that should be present in the observed spectrum. Since these may apex at slightly different times than the quantifier ion due to subtle differences in signal shape, EIC peaks within a small user-definable RI window of the quantifier ion are at this point gathered from the total peak list and added to the RI bin being queried. If enough qualifier ions with the expected intensities are found and the average deviation of all potential qualifier ions from expected intensities is below a set threshold, the match is passed and the peak area of the quantifier reported as the signal intensity for the matched analyte.

The algorithm supports both internal and external RI calibration and quantification based on multiple library-specified analyte-specific quantifier ions (a separate match attempt is made for each quantifier ion). Users may provide their own reference library or use one of the public libraries provided by MetabolomeExpress or one of its members. The MetabolomeExpress MSRI library format is a simple tab-delimited structure that should be easy to generate from other formats. Alternatively, libraries may be constructed in AMDIS format (*.MSL) using the freely available AMDIS software package http://chemdata.nist.gov/mass-spc/amdis/ and then converted to MetabolomeExpress format using the *MSRI Library Manager *tool that is part of *Database Explorer*. *MSRI Library Manager *also provides a facility to check whether the names of library entries are recognised by the MetabolomeExpress metabolite name recognition system.

### Comparison of MetabolomeExpress- and AMDIS-based MSRI library matching

The Automated Mass-spectral Deconvolution and Identification System (AMDIS) software package http://chemdata.nist.gov/mass-spc/amdis/ is widely regarded as one of the best freely available qualitative analysis tools for the deconvolution of complex GC/MS datasets and matching of GC/MS signals to reference libraries on the basis of mass spectra and retention indices. Notable metabolomics applications of AMDIS include creation and implementation of the popular Golm Metabolome Database 'Q_MSRI_ID' GC-Quadrupole-MS MSRI library [27] (freely available at http://csbdb.mpimp-golm.mpg.de) and integration with quantitative data processing tools such as the freely available MET-IDEA [9] and the proprietary Deconvolution and Reporting Software (DRS; Agilent Technologies; http://www.chem.agilent.com) to create metabolomics data processing workflows capable of both identification and quantification of metabolite signals in complex GC/MS metabolomics datasets.

To demonstrate the effectiveness of the MetabolomeExpress MSRI library matching workflow, we used AMDIS and MetabolomeExpress in parallel to identify metabolite signals in a representative GC/MS chromatogram produced by analysis of a typical complex biological extract (a crude methanolic extract of an Arabidopsis plant cell suspension culture) using the same MSRI library and compared the results (detailed in additional file 4; summarised in Table 1). For AMDIS processing, we used the same deconvolution settings as those used to generate the GMD 'Q_MSRI_ID' library [27] including two different adjustments of the AMDIS 'Sensitivity' setting ('low' and 'high'). Full details on AMDIS settings used are provided in additional file 4. For MetabolomeExpress processing, we also conducted two searches - one with default settings and one with the 'Min. Peak Area' setting adjusted from the default of 25,000 to 5,000 which will be referred to as 'low-sensitivity' (LS) and 'high-sensitivity' (HS) settings, respectively. This setting determines the minimum area value an EIC peak must have to be imported from the submitted peak list for RI-binning and library matching. Similarly to having a higher AMDIS 'Sensitivity' setting, having a lower 'Min. Peak Area' setting in MetabolomeExpress enables smaller peaks to be identified but increases the chances of false-positive detections or quantification based on small, noisy peaks which may give inaccurate results.

**Table 1:** Comparison of AMDIS and MetabolomeExpress MSRI library matching performance: summary of results.

	AMDIS		MetabolomeExpress	
**Settings:**	**Low Sensitivity**	**High Sensitivity**	**Low Sensitivity**	**High Sensitivity**

Total Peak Identifications Reported	152	165	153	170

True Positive	140	150	149	163

False Positive	7	12	0	1

False Negative	35	24	20	7

Ambiguous	5	5	6	7

A raw data file from a representative GC/MS analysis of a complex methanolic plant tissue extract was processed and searched against a single MSRI reference library using either AMDIS or MetabolomeExpress. Each software package was used under two different sensitivity settings to allow a more meaningful comparison of results. All peak identification results were manually verified by inspection of relevant raw GC/MS signals as true positives, false positives, false negatives or ambiguous calls. False negatives were cases where a search failed to identify a component that was successfully identified by another search. Detailed results including manual validation comments are presented in additional file 4.

After running all four searches, we aligned all the peak identification results in MS Excel before manually validating each call of analyte presence or absence by inspection of relevant raw GC/MS signals. The results of these manual validations, including justifications for our conclusions where any ambiguity between AMDIS and MetabolomeExpress results was apparent, are presented in additional file 4. In many cases where we observed false negative detections, we explain why these occurred. Based on our validations, we then classified each presence/absence call as a true positive, false positive, ambiguous positive, true negative, false negative or ambiguous negative. These results are summarised in Table 1.

Overall, the results from AMDIS and MetabolomeExpress were very similar with 141 (72%) of the total set of 197 peak identifications being reported by both programs. However, MetabolomeExpress outperformed AMDIS in all regards by reporting more true positives, fewer false/ambiguous positives and fewer false/ambiguous negatives (Table 1). This was particularly pronounced under HS settings where MetabolomeExpress gave 163 true positives compared to the 150 reported by AMDIS. However, the biggest strength of MetabolomeExpress relative to AMDIS was its lower false reporting rate. Under HS settings, AMDIS and MetabolomeExpress reported 15 and 7 false/ambiguous positives; and 26 and 8 false/ambiguous negatives, respectively (Table 1).

A common data-processing approach employed by metabolomics researchers is to use AMDIS-based peak deconvolution and identification to assist creation of target compound quantification libraries for specific quantifier ion-based quantification in their GC/MS manufacturer's proprietary data-analysis software (eg. Agilent's ChemStation). To demonstrate that the MetabolomeExpress workflow can achieve results of at least the same level of quality, we used the results generated in the above analysis to build an RT and quantifier ion-based ChemStation compound quantitation library for the 80 identified compounds of known structure and quantitatively processed the representative plant cell extract data file using ChemStation with an optimised sensitivity setting. Linear regression of ChemStation- and MetabolomeExpress-reported RTs and peak areas for the 78 target peaks successfully integrated by ChemStation (using the MetabolomeExpress values generated previously under HS settings, above) gave near perfect R^2 ^values of 1.0000 and 0.998, respectively. This demonstrates that the MetabolomeExpress workflow not only gives excellent qualitative performance, but quantitative performance as well. Moreover, construction of the RT-based quantitation library in ChemStation was a relatively laborious process that requires manual pre-definition of a limited number of qualifier ions via the ChemStation interface rather than dynamic selection of an unlimited number of qualifier ions based on deconvoluted library spectra that are easily manageable within tab-delimited files. Furthermore, unlike the RI-based approach of MetabolomeExpress, ChemStation-based quantitation would require either: a) regular re-adjustment of target RT settings to account for changes in RT associated with drift or column maintenance; or b) regular time-consuming 'retention time locking' of the instrument by fine tuning of carrier gas flow rates based on flow:RT calibration runs.

### Quantitative validation of the MetabolomeExpress data processing workflow

**Figure 3:**
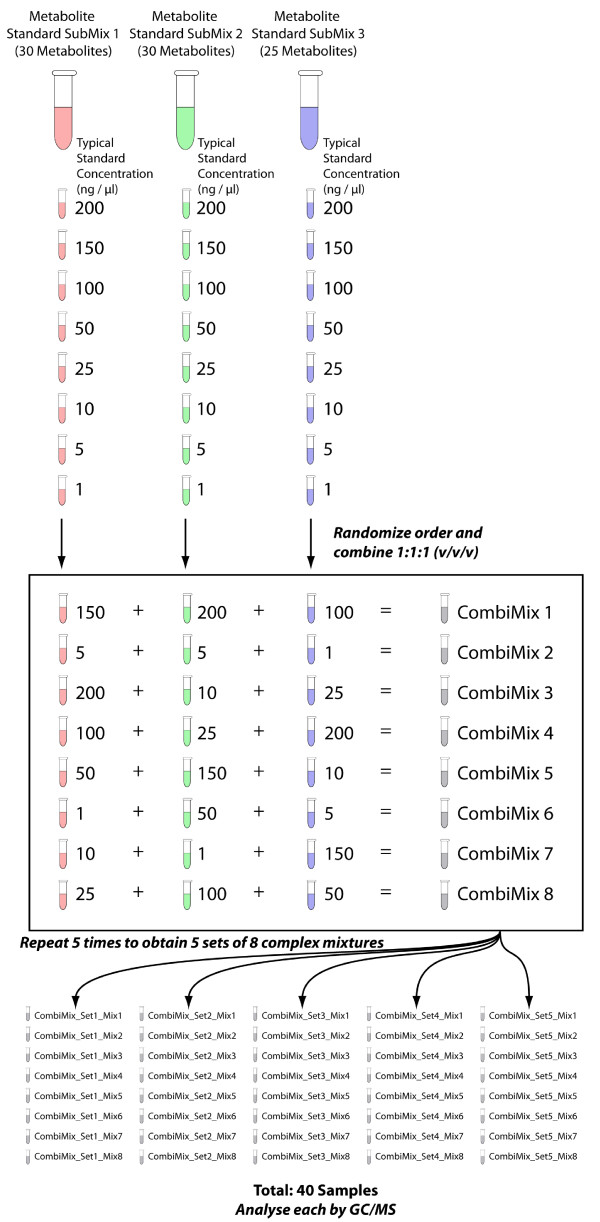
**Validation experiment: randomised combinatorial metabolite standard mixing**. Three metabolite mixtures each contained a set of approximately 30 different non-co-eluting high-purity authentic metabolite standards at known concentrations (typical metabolite concentration: 200 ng/μl). The components of these mixtures were chosen such that no chromatographic co-elution would occur between components of the same mixture but co-elution would occur between components of different mixtures when two or more of the mixtures were combined into a single analysis. An eight point dilution series was prepared from each of the three mixtures, generating three sets of eight solutions. The order of each dilution series was randomised to generate a randomised mixing protocol table and aliquots of solutions were combined accordingly. This randomised mixing process was repeated 5 times to generate 5 sets of 8 solutions (40 solutions). These complex solutions, each containing the same set of 85 metabolite standards, were analysed by a standard GC/MS metabolomics protocol.

In order to further quantitatively validate the MetabolomeExpress data processing workflow, we generated a challenging dataset through the GC/MS analysis of a set of complex standard mixtures carefully designed to introduce large known variations in the relative concentrations of co-eluting analytes and hence the relative intensities of overlapping or closely neighbouring signals. The procedure used to prepare these complex mixtures is detailed in Figure 3. Essentially, three simple mixtures each containing a unique set of 20-30 chromatographically resolved analytes were repeatedly combined in widely varying concentration ratios to generate a set of complex mixtures (each containing ~90 metabolites) in which co-elution of differentially diluted analytes would occur during GC/MS analysis. This dataset was chosen (as opposed to a simple single-mixture dilution series) to strengthen our validation by increasing the likelihood of misidentification or quantitative interference between overlapping or neighbouring signals. The raw dataset thus generated was processed using the MetabolomeExpress data processing pipeline including peak detection, MSRI library matching and data matrix construction steps. Data matrix construction included normalisation to the internal standard, ribitol, and automatic raw data-assisted missing value replacement whereby each missing value was replaced with the sum of the relevant EIC signal between the average integration start and end points for runs where the analyte with the missing value was successfully matched (see the MetabolomExpress User's Guide for details on data matrix construction).

**Figure 4:**
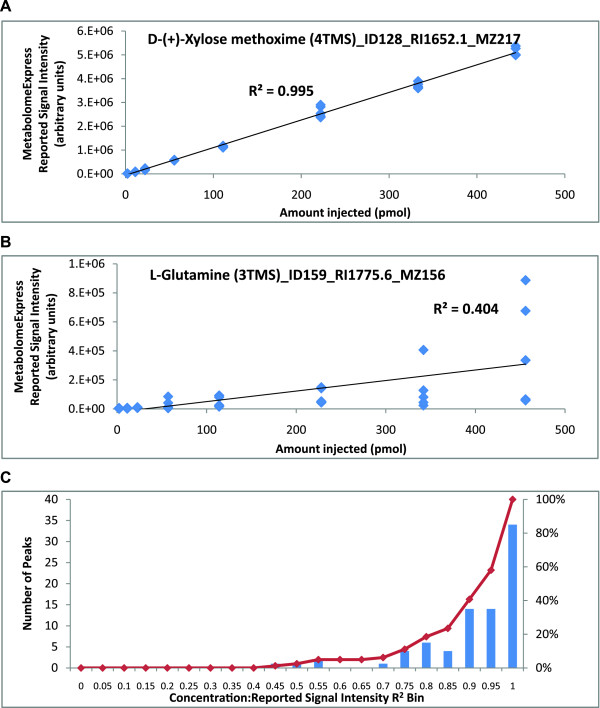
**Validation of the MetabolomeExpress MSRI Library Matching algorithm**. The challenge dataset described in Figure 3 was processed using MetabolomeExpress and the strength of the relationship between metabolite concentration and reported signal intensity assessed for each metabolite derivative peak by calculating coefficients of determination (ie. R^2 ^values). Linear regression plots are shown for (A) the best performing peak, Xylose (4TMS) and (B) the worst performing peak, Glutamine (3TMS). A histogram (C) shows the distribution of R^2 ^values across the 81 metabolite derivative peaks examined.

The basic premise of our validation approach was that if peak integration, library matching and data matrix construction worked correctly (ie. neighbouring signals were quantitatively resolved and correctly identified), then a strong relationship between the signal intensity ascribed to a particular metabolite and the known concentration of that metabolite should be observed. Therefore, to validate peak integration, library matching and data matrix construction, we calculated the coefficient of determination (R^2^) between concentration and reported signal intensity for 81 metabolite peaks representing a wide range of retention times and chemical classes (ie. all the peaks representing the standards in our test mixtures). Indeed, a strong average concentration:signal intensity R^2 ^value of 0.88 was observed when the best performing library spectrum and quantifier ion was selected for each of the 81 metabolite peaks (additional file 5). The best and worst performing peaks were Xylose methoxime (4TMS, quantified on m/z = 217; Figure 4A) and Glutamine (3TMS, quantified on m/z = 156; Figure 4B) with concentration:signal intensity R^2 ^values of 0.995 and 0.404, respectively. R^2 ^values were generally high with 66 (81%) of the 81 metabolite peaks showing an R^2 ^value > 0.8 (see Figure 4C for a histogram).

**Figure 5:**
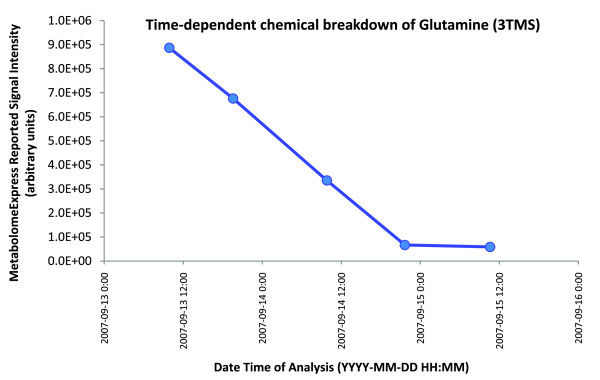
**Poor 'concentration:signal intensity' correlation for glutamine (3TMS) was due to its time-dependent consumption by an unknown chemical process**. The graph above shows the relationship between Glutamine (3TMS) signal intensity and time of analysis for 5 derivatised samples each originally supplied with the same amount of glutamine (450 pmol/μl). All samples were derivatised at the same time so a later time of analysis corresponds to a greater sample age.

To determine whether the poor performance of Glutamine (3TMS) was due to some kind of processing error, we manually inspected the Glutamine (3TMS) signals and found their sizes were consistent with reported intensity values. Upon further investigation, we found that, for samples originally containing the same amount of glutamine, the observed Glutamine (3TMS) signal was negatively correlated with the length of time between derivatisation and GC/MS analysis (Figure 5). This indicated that the high variability in Glutamine (3TMS) signal that caused the poor concentration:signal intensity correlation was due to an unknown time-dependent chemical process causing loss of Glutamine (3TMS) in samples over time. Interestingly, the signal for the only detectable Glutamine (4TMS) isomer was stable over the course of analysis (based on manual inspection of appropriate m/z 227 and 317 signals; data not shown) and did not exhibit a time-dependent increase as would be expected if the variability in Glutamine (3TMS) were due to slow derivatisation of the relatively inert amido group to form the observed Glutamine (4TMS). We could find no evidence for the presence of Glutamine (5TMS) or any reports in the literature of this theoretical derivative being detected by GC/MS. We therefore conclude that the time-dependent depletion of Glutamine (3TMS) was due to an unknown chemical process other than slow derivatisation.

### Statistics and exploratory data analysis

The *Statistics and Data Exploration *panel of the *Experiment Explorer *module currently provides tools to carry out data matrix construction, data matrix renormalisation, data matrix heatmap visualisation, Welch's t-tests, principal components analysis (PCA) and hierarchical clustering analysis (HCA). Statistical results are displayed in the web interface but may also be downloaded for offline analysis. Colour is used to aid data interpretation wherever possible. T-test results are presented as a red/blue heatmap table that allows results to be sorted by metabolite name, chemical class, retention time, retention index, signal intensity ratio or p-value. Wherever possible, displayed results are linked to their underlying raw GC/MS signals by point-and-click access - thus aiding manual verification of processed results. PCA plots are provided in 2D and 3D formats. PCA plots and HCA heatmap clustergrams are provided in vector formats for creation of publication quality figures.

**Figure 6:**
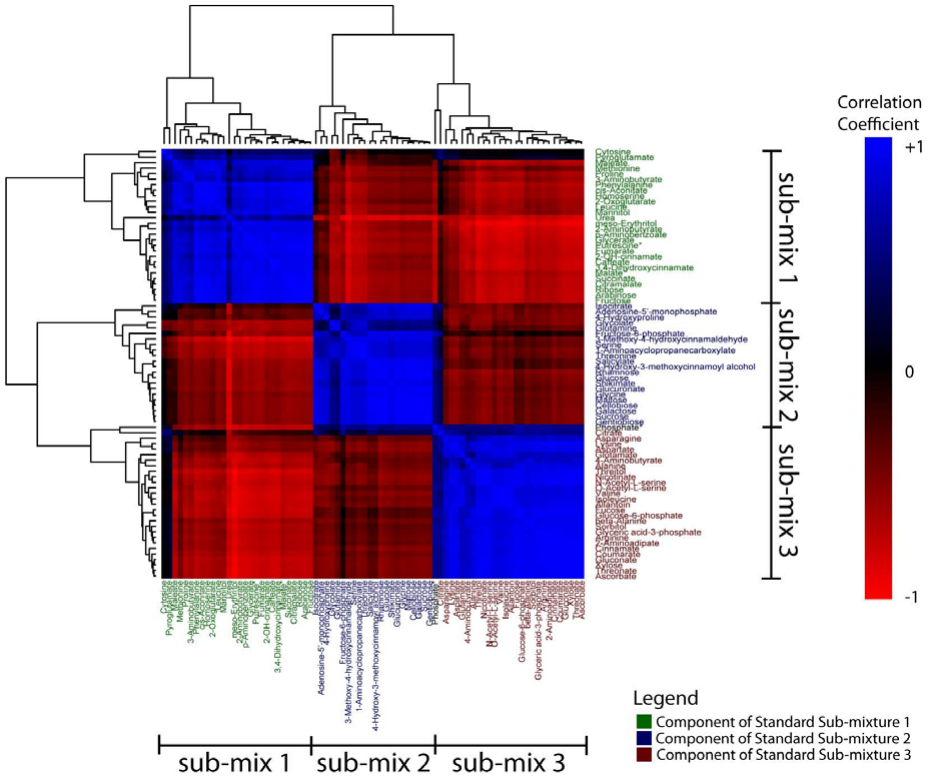
**Global validation by correlation analysis of a combinatorial metabolite standard mixing GC/MS data set**. The data matrix generated by MetabolomeExpress processing of the combinatorial standard mixing GC/MS data set (see Figure 3) was filtered to remove internal standards and analytes of unknown structure and then used to generate a correlation matrix which was used as input for hierarchical clustering in the statistical package, R. The reordered correlation matrix is shown as a heatmap with colours corresponding to analyte-analyte correlation coefficients (see color bar to the right). As expected, analytes were clustered into three major clusters corresponding to metabolite sub-mixes 1, 2 and 3 (see Figure 3). Analyte names have been coloured according to their metabolite submixture of origin (Mix 1 = red; Mix 2 = green; Mix 3 = blue). * Known breakdown product.

To further demonstrate the validity of MetabolomeExpress data processing, we performed hierarchical clustering analysis on the data matrix generated from the challenge dataset to show that we could resolve three major clusters of highly correlated metabolites (Figure 6) that corresponded precisely to the components of each of the three simple metabolite mixtures combined in different ratios to make each analysed complex mixture (see Figure 3).

### The Database Explorer

The *Database Explorer *module of MetabolomeExpress provides a number of tools with which to explore the contents of the MetabolomeExpress database of metabolite signal intensity ratio statistics. The first of these, *Database Statistics*, provides an overview of the current database contents and buttons to load experiments into the *Experiment Explorer *module for more detailed analysis. The second tool, *ResponseFinder*, is a simple query tool that allows users to search for metabolite responses of interest. Results are returned with links to experimental metadata and underlying raw GC/MS signals in the raw data viewer. The third tool is *MetaAnalyser*. This allows the results of multiple experiments to be aligned, clustered and compared in heatmap form.

### Future developments

Development of the MetabolomeExpress platform will be an ongoing process. Developments planned for MetabolomeExpress include: i) support for data exchange formats developed or endorsed by the Metabolomics Standards Initiative and similar initiatives; ii) enhanced capability for processing LC/MS and CE/MS metabolomic and quantitative proteomic data (including accurate-mass signals); iii) expansion of the suite of statistical data mining tools to include multivariate pattern matching tools to identify relationships between different metabolome response patterns and hence different biological processes; iv) enhanced facilities for capturing experimental metadata during experimental workflows; v) increased variety of import/export options for easier integration with external data processing workflows; vi) support for integrated analysis of parallel metabolite-, gene- and protein-expression data; and vii) a capacity to accept GET requests for information about experiments or statistical results so that results presented in electronic articles and other databases may be directly linked to MetabolomeExpress database content.

## Discussion

As GC/MS metabolomics becomes an increasingly mainstream bio-analytical technique, it is crucial that steps are taken to ensure published metabolomics data are accessible, reliable, reusable and transparent. This is largely the case in the microarray field where journals have enforced compliance to metadata reporting standards and submission of raw microarray data to online public repositories. However, the development of similar repositories for raw and processed metabolomics datasets is a far greater challenge due to the sheer size, complexity and heterogeneity of these datasets. Precisely for these reasons, it is essential that online metabolomics databases do not act merely as warehouses for raw and/or processed datasets that must be painstakingly downloaded and pieced back together manually using local desktop programs. Rather, online metabolomics databases should provide easy, in-situ access to the various levels of metabolomics datasets in an integrated, interactive manner. This is the philosophy we have adopted in the development of MetabolomeExpress. For an overview of the features that distinguish MetabolomeExpress from existing comparable GC/MS metabolomics web-tools, see Table 2.

**Table 2:** Features distinguishing MetabolomeExpress from existing GC/MS metabolomics web-tools.

	PlantMetabolomics.org	SetupX	MeltDB	MetaboAnalyst	MetabolomeExpress
**Peer-reviewed biological content**					

Number of peer-reviewed, biology-focused publication datasets publicly available	0*	0	0	0	8

**Data upload/storage**					

Accepts public raw data submissions				+	+
Accepts public processed data submissions	+				+
Long-term data storage	+	+	+		+

**Data resources for download**					

MSI-compliant metadata	+	+			+
Raw GC/MS data files		+			+
Mass peak lists		+			+
Library match lists		+			+
Data matrices	+	+			+
Mass-spectral and retention-index libraries					+
Precomputed fold-change/comparative statistical results	+				+
PCA Results			+		+
HCA Results			+		+
Statistical heatmap spreadsheets					+
Metabolite-metabolite correlation network graphs					+
Metabolite-metabolite correlation tables					+
MapMan importable fold-change data files					+
Cytoscape-importable fold-change data files					+

**Raw data processing**					

Provides integrated access to peak detection			+	+	+
Provides own peak detection algorithm					+
Performs peak identification without offline pre-processing					+
Supports upload of custom MSRI libraries					+

**Statistics**					

Allows users to perform custom statistical comparisons	+		+	+	+
Fold change	+		+	+	+
t-test			+	+	+
Principal Components Analysis (PCA)			+	+	+
Cluster Analysis			+	+	+
Metabolite-metabolite correlation Analysis					+

**Raw data visualisation**					

Provides chromatogram visualisation			+		+
Chromatogram viewer displays TICs			+		+
Chromatogram viewer displays EICs					+
Chromatogram viewer allows overlays of multiple chromatograms					+
Chromatogram viewer zoomable					+
Provides MS spectral visualisation			+		+
Provides MS spectra of library entries			+		+
Provides MS spectra of arbitrary MS scans					+

Features of MetabolomeExpress that distinguish it from the existing GC/MS metabolomics web-tools - PlantMetabolomics.org ([28]), SetupX [15], MeltDB [14] and MetaboAnalyst [13] - are listed. A '+' indicates that a feature is present. * We were unable to find any information indicating that any of the datasets stored in PlantMetabolomics.org have been published in peer-reviewed, biology-focused research articles.

## Conclusions

MetabolomeExpress https://www.metabolome-express.org provides a new opportunity for the metabolomics community to transparently and interactively present online the raw and processed GC/MS metabolomics data underlying their research findings. Transparent sharing of these data has the potential to increase the value of metabolomics publications by allowing the broader research community to assess data quality and draw their own insights from disseminated datasets. Moreover, the centralised storage and systematic annotation of metabolite response information from diverse biological systems and experimental perturbations, together with meta-analysis tools, will reveal previously unrecognised patterns and thus facilitate mechanistic and evolutionary discoveries that would have otherwise been far more difficult to make.

### Availability and requirements

The MetabolomeExpress web application is freely accessible for non-commercial, academic use at https://www.metabolome-express.org. Registered users may access the FTP repository at ftp://www.metabolome-express.org.

The major client hardware and software requirements to use the MetabolomeExpress web interface and FTP server are:

• A modern version of one of the major web browsers (ie. Microsoft Internet Explorer, Mozilla Firefox, Google Chrome or Safari)

• Minimum of 512 MB of RAM (1GB+ recommended)

• A relatively fast internet connection

• A two-button mouse

• An FTP client program (eg. FileZilla; Only required if uploading data).

## Authors' contributions

AJC carried out project conception, programming, testing, wet experimental work and wrote the manuscript. AHM and MRB critically revised the manuscript. All authors read and approved of the final manuscript.

## Supplementary Material

Additional file 1 **MetabolomeExpress v1 User's Guide**. The current MetabolomeExpress user's manual including detailed instructions and examples on how to upload and manage datasets via FTP and how to use all current features of the MetabolomeExpress web interface. Click here for file

Additional file 2 **Notes on FTP repository and data formats**. Detailed comments about the MetabolomeExpress FTP repository and data formats supported by MetabolomeExpress and rationales behind their designs. Click here for file

Additional file 3 **MetabolomeExpress peak detection validation results**. Comparison of peak integration results from Agilent ChemStation and MetabolomeExpress *PeakFinder *algorithm. Includes detailed peak detection information. Click here for file

Additional file 4 **Comparison of MetabolomeExpress Library Matching with AMDIS and ChemStation**. Detailed results of the comparison of AMDIS-based analyte signal identification (and subsequent ChemStation-based quantitation) with the integrated identification and quantitation approach of MetabolomeExpress (described in the section entitled 'Comparison of MetabolomeExpress- and AMDIS-based MSRI library matching' with results summarised in Table 1). Click here for file

Additional file 5 **MetabolomeExpress validation - standard curves**. The data matrix obtained by MetabolomeExpress processing of the dataset described in Figure 3. This matrix includes R^2 ^values describing the relationships between metabolite concentration and reported signal intensity, as determined by MetabolomeExpress. Click here for file
