# Supplementary material for: The MetabolomeExpress Project: enabling web-based processing, analysis and transparent dissemination of GC/MS metabolomics datasets
Source: BMC Bioinformatics. 2010 Jul 14;11:376. doi: 10.1186/1471-2105-11-376 (PMC2912306; doi:10.1186/1471-2105-11-376)
Supplement: Additional file 1 — MetabolomeExpress v1 User's Guide. The current MetabolomeExpress user's manual including detailed instructions and examples on how to upload and manage datasets via FTP and how to use all current features of the MetabolomeExpress web interface. [file 1471-2105-11-376-S1.PDF]

The MetabolomeExpress User's Guide (v 1.0)

By Dr Adam J. Carroll

QUICK START

Load a dataset by left-clicking or right-clicking (or control-clicking if you are using a single-button mouse on an Apple computer) on the experiment folder and left-clicking 'Load Folder Contents'

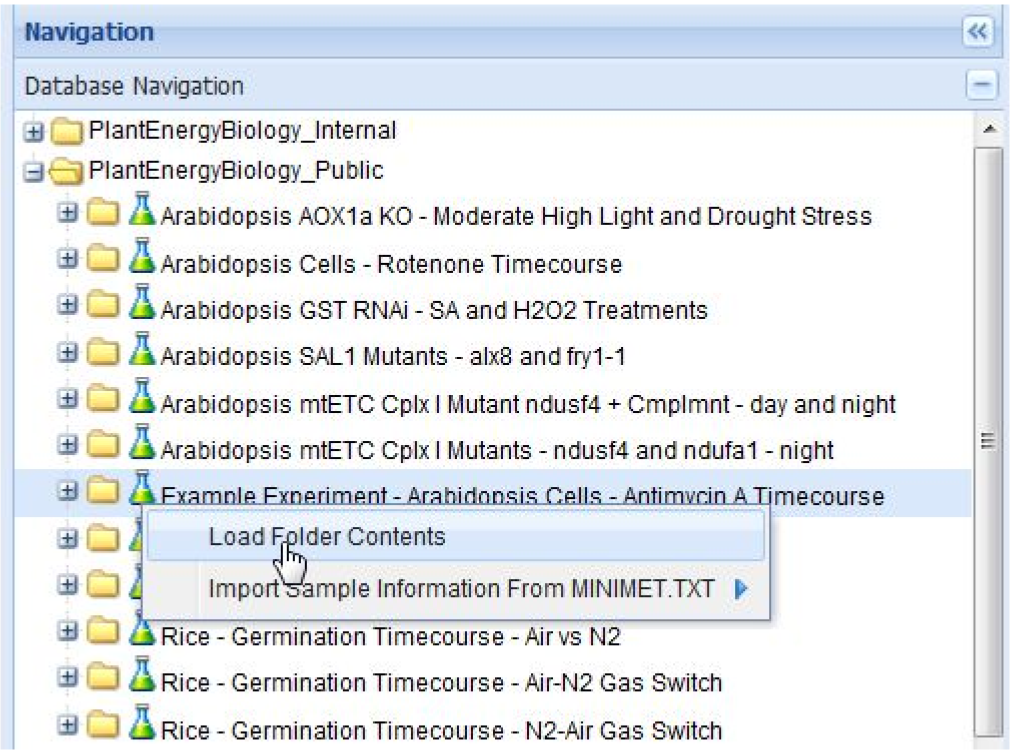

Table of Contents

1 MetabolomeExpress: Getting started 4

1.1 Structural overview 4

1.2 Public vs. private data access 5

1.3 Obtaining your own MetabolomeExpress data repository: Registration 5

1.4 Uploading and managing your datasets via FTP 6

1.4.1 Logging in 6

Step 1 - Create a data folder for the experiment 6

Step 2 - Upload raw GC/MS files 6

Step 3 - Perform Data Import and Peak Detection 7

Step 4 - Upload sample information table: 7

Step 5 - Upload a retention index calibration file 10

1.4.2 Choosing a research area and importing sample information..................11

1.5 Building and using custom Mass Spectral and Retention Index (MSRI) libraries 13

1.5.1 An overview of the MetabolomeExpress MSRI library format..................13

1.5.2 Creating MSRI libraries from AMDIS .MSL files 15

1.5.3 Adding metadata to MSRI libraries 18

1.5.4 Displaying and Validating MSRI Libraries with MSRI Library Manager ...20

1.5.5 Using Analyte Annotations Tables to customise data filtering 20

1.6 Supported Data Formats (including example files)..22

1.6.1 The main MetabolomeExpress metadata exchange format (*_METADATA.TXT) 22

1.6.2 Raw GC/MS data: NetCDF and the MetabolomeExpress eXtracted Ion Chromatogram (.XIC) format..27

1.6.3 Peak list tables (.PEAKLIST) 27

1.6.4 MSRI library matching report tables (.MATCHREPORT).........................28

1.6.5 Data matrices (.mzrtMATRIX)..29

2 Re-analysis of publicly disseminated data using *Database Explorer* 31

2.1 Getting an overview of the database with the *Database Statistics* panel 31

2.2 Finding experiments of interest using *ResponseFinder* 32

2.3 Comparing metabolite response patterns across multiple publications using *MetaAnalyser*..33

2.4 Identifying phenocopies using PhenoMeter (in development)........................36

3 Processing and analysis of experimental GC/MS datasets with *Experiment Explorer* 38

3.1 Example dataset: Timecourse metabolomic analysis of plant cells treated with Antimycin A..38

3.2 Loading a dataset..38

3.2.1 Loading a dataset from the Navigation panel 38

3.3 Using the Raw Data Viewer 39

3.4 The raw data viewer control panel 40

3.5 Zooming in and out 41

3.6 Viewing mass spectral scans 42

3.7 Finding differentially expressed peaks using the chromatographic statistical tool 42

4 Data import and peak detection (registered users only)......................................43

4.1 A Guide to the MetabolomeExpress PeakFinder Algorithm 44

4.2 The Peak Detection Control Panel 45

5 MSRI library matching...46

5.1 How to conduct an MSRI library matching process..46

Mass-Spectral Tag (MST) reconstruction and the MSRI library matching process..47

5.2 Interacting with MSRI library matching results 47

6 Statistics and data exploration 49

6.1 Construction of data matrices from MSRI library-matching reports 50

6.1.1 Some notes on normalisation and quality control 50

6.1.2 Raw data-assisted missing value replacement.......................................51

6.1.3 How to build a data matrix using the *Match Report to Data Matrix* tool ...51

6.2 Matrix renormalisation...54

6.3 Using the interactive matrix explorer 55

6.4 Comparative statistics..56

6.5 Principal Components Analysis (PCA) 58

6.6 Hierarchical Cluster Analysis (HCA) ... 60

6.7 Correlation network construction ... 63

6.8 Submitting a dataset to the main database of metabolite response statistics 65

7 APPENDIX A – Interpretation of MetabolomeExpress Metadata Validation Templates ... 67

7.1 Background ... 67

7.2 Interpretation of MetabolomeExpress Metadata Template Files ... 67

7.3 Validation Codes ... 69

7.4 Data types and associated configuration parameters ... 69

1 MetabolomeExpress: Getting started

1.1 Structural overview

MetabolomeExpress is comprised of three interacting layers:

1. An FTP repository where registered users may upload and manage their own GC/MS datasets

2. A MySQL database that stores general metabolite information and metabolite response statistics from datasets present in the quality-controlled MetabolomeExpress database of metabolite response statistics.

3. A web interface to interact with data present in the FTP repository and MySQL database

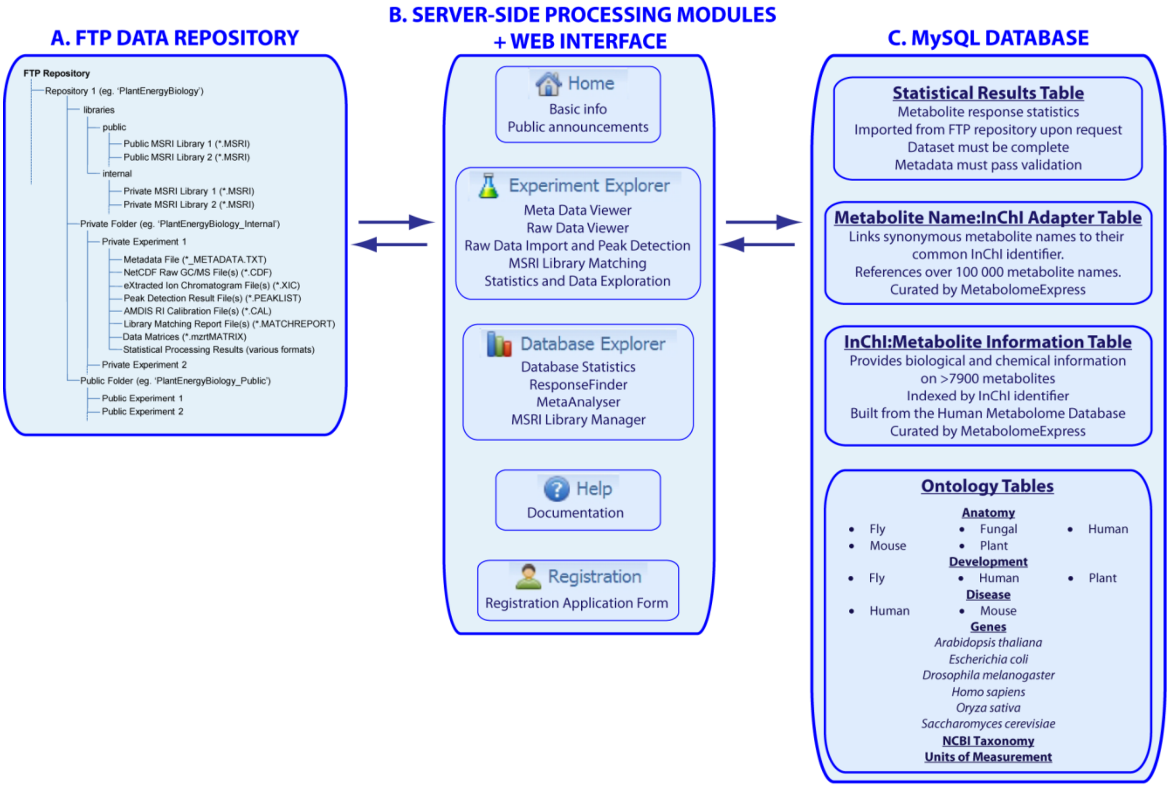

Figure 1. Structural overview of MetabolomeExpress

1.2 Public vs. private data access

MetabolomeExpress houses both public and private data (including GC/MS libraries). Public data is accessible to anonymous users (ie. users automatically logged in as 'guest') via the web interface. Private data may only be accessed by registered users who are logged in and have permission to access the private data (see Section 1.3 below for details).

1.3 Obtaining your own MetabolomeExpress data repository: Registration

In order to analyse and share your own GC/MS datasets with MetabolomeExpress, you must first register to obtain a username and password.

Registration is FREE.

Once your application to register has been received, you will be contacted by email to determine whether you wish to create a new repository (and if so, what you want to call the repository) or simply to join an existing repository. If you wish to join an existing repository, we will require authorisation (via email) from the original creator of that repository. It IS possible for you to have your own repository and also have access to another repository via the web-interface - we just need to receive an email request to give you repository access permissions from the owner of the other repository. NOTE: Each registered username will obtain FTP access to only one repository. If you wish to upload data to another repository, you must obtain the

username and password from an FTP user of that repository or register for another username and password for use with that FTP repository.

1.4 Uploading and managing your datasets via FTP

1.4.1 Logging in

To upload and manage your datasets via FTP, you will need an FTP client program. We use the free FTP client, FileZilla, but most FTP clients should work fine. To connect to your FTP repository, enter the following into your FTP client program:

Hostname: www.metabolome-express.org

username: your username

password: your password

and connect.

Once connected, you should see your repository.

This screenshot below shows an example of the result of logging in as AdamC using FileZilla:

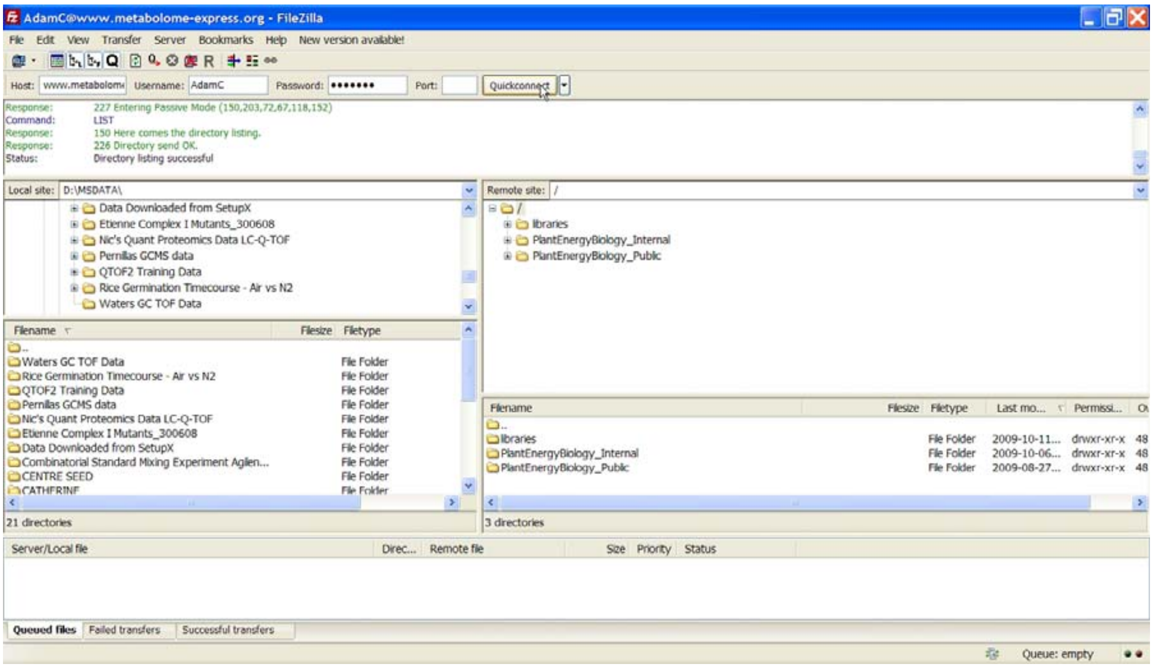

Once connected, there are a few simple steps to create a new experiment:

Step 1 - Create a data folder for the experiment: Open either your public or internal data folder and create a new subfolder with the name of the folder being the name of the experiment (eg. 'Nutrient supplementation timecourse 1').

Step 2 - Upload raw GC/MS files: Copy all the raw NetCDF/ANDI MS/AIA GC/MS files (.CDF) for that experiment into the folder you just created. If you don't have your files

in NetCDF format, you should be able to export them from your instrument manufacturer's data processing software.

HINT: It will make things easier for everyone if you name your files descriptively rather than with meaningless numbers or letters. Name your files something like '20091015_Nutritional Regime A_Day 1_Replicate 1.CDF', '20091015_Nutritional Regime A_Day 1_Replicate 2.CDF' etc. (where the first 8 numbers represent the date on which the batch sequence of GC/MS runs began, in YYYYMMDD format) rather than something like '1.CDF', '2.CDF' etc.

Step 3 - Perform Data Import and Peak Detection: Now, log in to MetabolomeExpress and you should be able to find your new experiment folder containing your data files in the 'Database Navigation' panel on the left hand side of the interface. To begin processing your files, right click on the experiment folder name and left click 'Load folder contents'. Your files should now be ready for selection in the Data Import and Peak Detection Control Panel inside the Data Import and Peak Detection tab (see Section 3.4 - Data Import and Peak Detection). You may perform the data import and peak detection process while completing the next two steps required for library matching, data matrix construction and statistical analysis.

Step 4 - Upload sample information table: The next step is to provide basic sample information required for signal normalisation and statistical grouping. This information is provided in the form of a simple tab-delimited table which must be called MINIMET.TXT (which stands for MINImal METadata). This simple metadata file can be used to construct a starting template of the somewhat more complex _METADATA.TXT metadata format. This allows you to process your data and get statistical results quickly without having to spend time completing the larger _METADATA.TXT file. Many of the fields in the larger format are not required for data processing, but are required for proper Metabolomics Standards Initiative (MSI)-compliant public dataset dissemination.

The MINIMET.TXT format has 6 columns which must be labeled 'Sample ID', 'Genotype', 'Treatment', 'Organ or Biomaterial Type', 'Timepoint' and 'Sample Mass or Volume'. The order of the columns is not important, but the column headings must be exactly as shown. Each row represents a single GC/MS run.

The information to put in each column is as follows:

Sample ID - This is the name of the NetCDF GC/MS data file (without the '.CDF' extension)

Genotype - This is the short name of the genotype of the organism that was analysed in the sample. Make sure that all samples of the same genotype have *exactly* the same entry here. Try to use a well established short name if there is one.

Treatment - This is a short descriptive ID for the experimental treatment applied to the organism analysed in the sample. Different treatment durations or treatment doses are considered different treatments and must be given different IDs. Make sure that all samples of the same treatment have *exactly* the same entry here.

Organ or Biomaterial Type - This is the standard name of the organ/tissue/biofluid type that was used to prepare the sample. Check reporting standards in your biological field for the appropriate controlled vocabulary or ontology to use here. Make sure that all samples of the same organ or biomaterial type have *exactly* the same entry here.

Timepoint - This is the time of harvest of the sample with respect to the beginning of the treatment period. Make sure that all samples of the same timepoint have *exactly* the same entry here.

Sample Mass or Volume - This is the mass or volume of the biological sample that was extracted and analysed. The units do not matter at this stage. They may be added to the more detailed _METADATA.TXT file later. These numeric values will be used to normalise signal intensities later during data matrix construction.

We will now run through some examples to demonstrate how you can set up MINIMET.TXT files for different types of experiments.

The screenshot below shows an example MINIMET.TXT file being created in Microsoft Excel. You can probably tell that the file represents an experiment where two genotypes of animal have each been fed on two different diets and their blood has been collected one day and two days after beginning of the diet-feeding period.

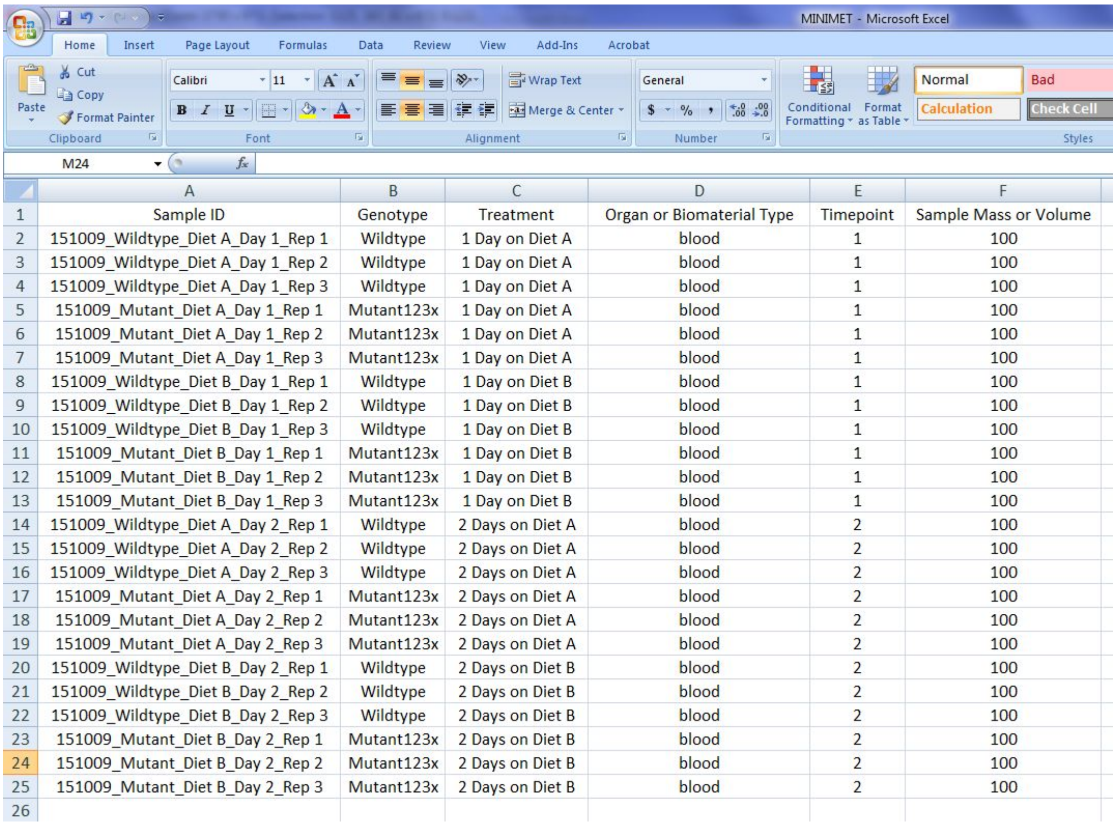

	A	B	C	D	E	F
1	Sample ID	Genotype	Treatment	Organ or Biomaterial Type	Timepoint	Sample Mass or Volume
2	151009_Wildtype_Diet A_Day 1_Rep 1	Wildtype	1 Day on Diet A	blood	1	100
3	151009_Wildtype_Diet A_Day 1_Rep 2	Wildtype	1 Day on Diet A	blood	1	100
4	151009_Wildtype_Diet A_Day 1_Rep 3	Wildtype	1 Day on Diet A	blood	1	100
5	151009_Mutant_Diet A_Day 1_Rep 1	Mutant123x	1 Day on Diet A	blood	1	100
6	151009_Mutant_Diet A_Day 1_Rep 2	Mutant123x	1 Day on Diet A	blood	1	100
7	151009_Mutant_Diet A_Day 1_Rep 3	Mutant123x	1 Day on Diet A	blood	1	100
8	151009_Wildtype_Diet B_Day 1_Rep 1	Wildtype	1 Day on Diet B	blood	1	100
9	151009_Wildtype_Diet B_Day 1_Rep 2	Wildtype	1 Day on Diet B	blood	1	100
10	151009_Wildtype_Diet B_Day 1_Rep 3	Wildtype	1 Day on Diet B	blood	1	100
11	151009_Mutant_Diet B_Day 1_Rep 1	Mutant123x	1 Day on Diet B	blood	1	100
12	151009_Mutant_Diet B_Day 1_Rep 2	Mutant123x	1 Day on Diet B	blood	1	100
13	151009_Mutant_Diet B_Day 1_Rep 3	Mutant123x	1 Day on Diet B	blood	1	100
14	151009_Wildtype_Diet A_Day 2_Rep 1	Wildtype	2 Days on Diet A	blood	2	100
15	151009_Wildtype_Diet A_Day 2_Rep 2	Wildtype	2 Days on Diet A	blood	2	100
16	151009_Wildtype_Diet A_Day 2_Rep 3	Wildtype	2 Days on Diet A	blood	2	100
17	151009_Mutant_Diet A_Day 2_Rep 1	Mutant123x	2 Days on Diet A	blood	2	100
18	151009_Mutant_Diet A_Day 2_Rep 2	Mutant123x	2 Days on Diet A	blood	2	100
19	151009_Mutant_Diet A_Day 2_Rep 3	Mutant123x	2 Days on Diet A	blood	2	100
20	151009_Wildtype_Diet B_Day 2_Rep 1	Wildtype	2 Days on Diet B	blood	2	100
21	151009_Wildtype_Diet B_Day 2_Rep 2	Wildtype	2 Days on Diet B	blood	2	100
22	151009_Wildtype_Diet B_Day 2_Rep 3	Wildtype	2 Days on Diet B	blood	2	100
23	151009_Mutant_Diet B_Day 2_Rep 1	Mutant123x	2 Days on Diet B	blood	2	100
24	151009_Mutant_Diet B_Day 2_Rep 2	Mutant123x	2 Days on Diet B	blood	2	100
25	151009_Mutant_Diet B_Day 2_Rep 3	Mutant123x	2 Days on Diet B	blood	2	100
26						

In some types of experiments, such as human clinical metabolomics experiments, different disease states are considered different treatments. The example below shows a dummy clinical experiment investigating the urine metabolome for interactions between disease and drug treatment at two different time points with

respect to some starting time point which you can explain later in the more detailed metadata format if necessary.

	A	B	C	D	E	F
1	Sample ID	Genotype	Treatment	Organ or Biomaterial Type	Timepoint	Sample Mass or Volume
2	151009_Wildtype_Disease_Drug_Week 1_Rep 1	Wildtype	Disease + drug_week 1	urine	1	100
3	151009_Wildtype_Disease_Drug_Week 1_Rep 2	Wildtype	Disease + drug_week 1	urine	1	100
4	151009_Wildtype_Disease_Drug_Week 1_Rep 3	Wildtype	Disease + drug_week 1	urine	1	100
5	151009_Wildtype_Disease_Placebo_Week 1_Rep 1	Wildtype	Disease + placebo_week 1	urine	1	100
6	151009_Wildtype_Disease_Placebo_Week 1_Rep 2	Wildtype	Disease + placebo_week 1	urine	1	100
7	151009_Wildtype_Disease_Placebo_Week 1_Rep 3	Wildtype	Disease + placebo_week 1	urine	1	100
8	151009_Wildtype_Healthy_Drug_Week 1_Rep 1	Wildtype	Healthy + drug_week 1	urine	1	100
9	151009_Wildtype_Healthy_Drug_Week 1_Rep 2	Wildtype	Healthy + drug_week 1	urine	1	100
10	151009_Wildtype_Healthy_Drug_Week 1_Rep 3	Wildtype	Healthy + drug_week 1	urine	1	100
11	151009_Wildtype_Healthy_Placebo_Week 1_Rep 1	Wildtype	Healthy + placebo_week 1	urine	1	100
12	151009_Wildtype_Healthy_Placebo_Week 1_Rep 2	Wildtype	Healthy + placebo_week 1	urine	1	100
13	151009_Wildtype_Healthy_Placebo_Week 1_Rep 3	Wildtype	Healthy + placebo_week 1	urine	1	100
14	151009_Wildtype_Disease_Drug_Week 4_Rep 1	Wildtype	Disease + drug_week 2	urine	4	100
15	151009_Wildtype_Disease_Drug_Week 4_Rep 2	Wildtype	Disease + drug_week 2	urine	4	100
16	151009_Wildtype_Disease_Drug_Week 4_Rep 3	Wildtype	Disease + drug_week 2	urine	4	100
17	151009_Wildtype_Disease_Placebo_Week 4_Rep 1	Wildtype	Disease + placebo_week 2	urine	4	100
18	151009_Wildtype_Disease_Placebo_Week 4_Rep 2	Wildtype	Disease + placebo_week 2	urine	4	100
19	151009_Wildtype_Disease_Placebo_Week 4_Rep 3	Wildtype	Disease + placebo_week 2	urine	4	100
20	151009_Wildtype_Healthy_Drug_Week 4_Rep 1	Wildtype	Healthy + drug_week 2	urine	4	100
21	151009_Wildtype_Healthy_Drug_Week 4_Rep 2	Wildtype	Healthy + drug_week 2	urine	4	100
22	151009_Wildtype_Healthy_Drug_Week 4_Rep 3	Wildtype	Healthy + drug_week 2	urine	4	100
23	151009_Wildtype_Healthy_Placebo_Week 4_Rep 1	Wildtype	Healthy + placebo_week 2	urine	4	100
24	151009_Wildtype_Healthy_Placebo_Week 4_Rep 2	Wildtype	Healthy + placebo_week 2	urine	4	100
25	151009_Wildtype_Healthy_Placebo_Week 4_Rep 3	Wildtype	Healthy + placebo_week 2	urine	4	100

Sometimes, the “disease state” is more to do with genotype. For example, if you were doing an experiment to compare the metabolomics responses of a normal mammalian cell line and some mutated cancer cell line to a treatment like, say, hypoxia, your file might look something like this:

	A	B	C	D	E	F
1	Sample ID	Genotype	Treatment	Organ or Biomaterial Type	Timepoint	Sample Mass or Volume
2	151009_wildtype_hypoxia_Rep 1	wildtype cell line	Hypoxia	cultured cell	1	100
3	151009_wildtype_hypoxia_Rep 2	wildtype cell line	Hypoxia	cultured cell	1	100
4	151009_wildtype_hypoxia_Rep 3	wildtype cell line	Hypoxia	cultured cell	1	100
5	151009_wildtype_hypoxia_Rep 4	wildtype cell line	Hypoxia	cultured cell	1	100
6	151009_wildtype_hypoxia_Rep 5	wildtype cell line	Hypoxia	cultured cell	1	100
7	151009_wildtype_hypoxia_Rep 6	wildtype cell line	Hypoxia	cultured cell	1	100
8	151009_wildtype_normoxia_Rep 1	wildtype cell line	Normoxia	cultured cell	1	100
9	151009_wildtype_normoxia_Rep 2	wildtype cell line	Normoxia	cultured cell	1	100
10	151009_wildtype_normoxia_Rep 3	wildtype cell line	Normoxia	cultured cell	1	100
11	151009_wildtype_normoxia_Rep 4	wildtype cell line	Normoxia	cultured cell	1	100
12	151009_wildtype_normoxia_Rep 5	wildtype cell line	Normoxia	cultured cell	1	100
13	151009_wildtype_normoxia_Rep 6	wildtype cell line	Normoxia	cultured cell	1	100
14	151009_cancer cells_hypoxia_Rep 1	cancer cell line	Hypoxia	cultured cell	1	100
15	151009_cancer cells_hypoxia_Rep 2	cancer cell line	Hypoxia	cultured cell	1	100
16	151009_cancer cells_hypoxia_Rep 3	cancer cell line	Hypoxia	cultured cell	1	100
17	151009_cancer cells_hypoxia_Rep 4	cancer cell line	Hypoxia	cultured cell	1	100
18	151009_cancer cells_hypoxia_Rep 5	cancer cell line	Hypoxia	cultured cell	1	100
19	151009_cancer cells_hypoxia_Rep 6	cancer cell line	Hypoxia	cultured cell	1	100
20	151009_cancer cells_normoxia_Rep 1	cancer cell line	Normoxia	cultured cell	1	100
21	151009_cancer cells_normoxia_Rep 2	cancer cell line	Normoxia	cultured cell	1	100
22	151009_cancer cells_normoxia_Rep 3	cancer cell line	Normoxia	cultured cell	1	100
23	151009_cancer cells_normoxia_Rep 4	cancer cell line	Normoxia	cultured cell	1	100
24	151009_cancer cells_normoxia_Rep 5	cancer cell line	Normoxia	cultured cell	1	100
25	151009_cancer cells_normoxia_Rep 6	cancer cell line	Normoxia	cultured cell	1	100

Sometimes you may be interested in comparing the metabolomes of different parts of an organism under different conditions. For example, the screenshot below shows a MINIMET.TXT file for a hypothetical experiment comparing the metabolomes of plant roots and shoots under normoxic and hypoxic conditions.

Sample ID	Genotype	Treatment	Organ or Biomaterial Type	Timepoint	Sample Mass or Volume
151009_shoot_hypoxia_Rep 1	wildtype	Hypoxia	shoot	1	100
151009_shoot_hypoxia_Rep 2	wildtype	Hypoxia	shoot	1	100
151009_shoot_hypoxia_Rep 3	wildtype	Hypoxia	shoot	1	100
151009_shoot_hypoxia_Rep 4	wildtype	Hypoxia	shoot	1	100
151009_shoot_hypoxia_Rep 5	wildtype	Hypoxia	shoot	1	100
151009_shoot_hypoxia_Rep 6	wildtype	Hypoxia	shoot	1	100
151009_shoot_normoxia_Rep 1	wildtype	Normoxia	shoot	1	100
151009_shoot_normoxia_Rep 2	wildtype	Normoxia	shoot	1	100
151009_shoot_normoxia_Rep 3	wildtype	Normoxia	shoot	1	100
151009_shoot_normoxia_Rep 4	wildtype	Normoxia	shoot	1	100
151009_shoot_normoxia_Rep 5	wildtype	Normoxia	shoot	1	100
151009_shoot_normoxia_Rep 6	wildtype	Normoxia	shoot	1	100
151009_root_hypoxia_Rep 1	wildtype	Hypoxia	root	1	100
151009_root_hypoxia_Rep 2	wildtype	Hypoxia	root	1	100
151009_root_hypoxia_Rep 3	wildtype	Hypoxia	root	1	100
151009_root_hypoxia_Rep 4	wildtype	Hypoxia	root	1	100
151009_root_hypoxia_Rep 5	wildtype	Hypoxia	root	1	100
151009_root_hypoxia_Rep 6	wildtype	Hypoxia	root	1	100
151009_root_normoxia_Rep 1	wildtype	Normoxia	root	1	100
151009_root_normoxia_Rep 2	wildtype	Normoxia	root	1	100
151009_root_normoxia_Rep 3	wildtype	Normoxia	root	1	100
151009_root_normoxia_Rep 4	wildtype	Normoxia	root	1	100
151009_root_normoxia_Rep 5	wildtype	Normoxia	root	1	100
151009_root_normoxia_Rep 6	wildtype	Normoxia	root	1	100

Once you have completed the MINIMET.TXT file, save it as a tab-delimited file and upload it into the experiment folder by FTP.

Step 5 - Upload a retention index calibration file: The next data processing step after data import and peak detection is MSRI library matching. However, to perform MSRI library matching, MetabolomeExpress requires a small retention index calibration file to be present in the experiment FTP folder. The format used for this is the AMDIS *.CAL format. This is just a small tab-delimited text file providing the retention times and Kovats retention indices of a set of retention index (RI) calibration compounds (eg. alkanes) spanning the retention time range of the used GC/MS method. You can either add these compounds to your samples prior to analysis (highly recommended) or analyse them in a separate run added to the same instrument batch sequence as your actual biological samples - either way, you will need to determine the retention time of each RI calibration compound in at least one representative run from your batch. We use the MetabolomeExpress raw data viewer or the freely available AMDIS software to identify the peaks for a series of alkanes and enter their retention times into a template .CAL file using a text editor.

You can easily create .CAL files yourself in a spreadsheet (just be sure to save as a tab-delimited text file with extension .cal or .CAL) or you could modify an example file. The screenshot below shows the simple structure of the format. The file has no column labels. The first column is the retention time in minutes (to three or more decimal places). The second column is the Kovats RI. These first two columns need to

be filled for use with MetabolomeExpress. The third, fourth and fifth columns are used by AMDIS but not by MetabolomeExpress. Leaving them filled is optional for use with MetabolomeExpress.

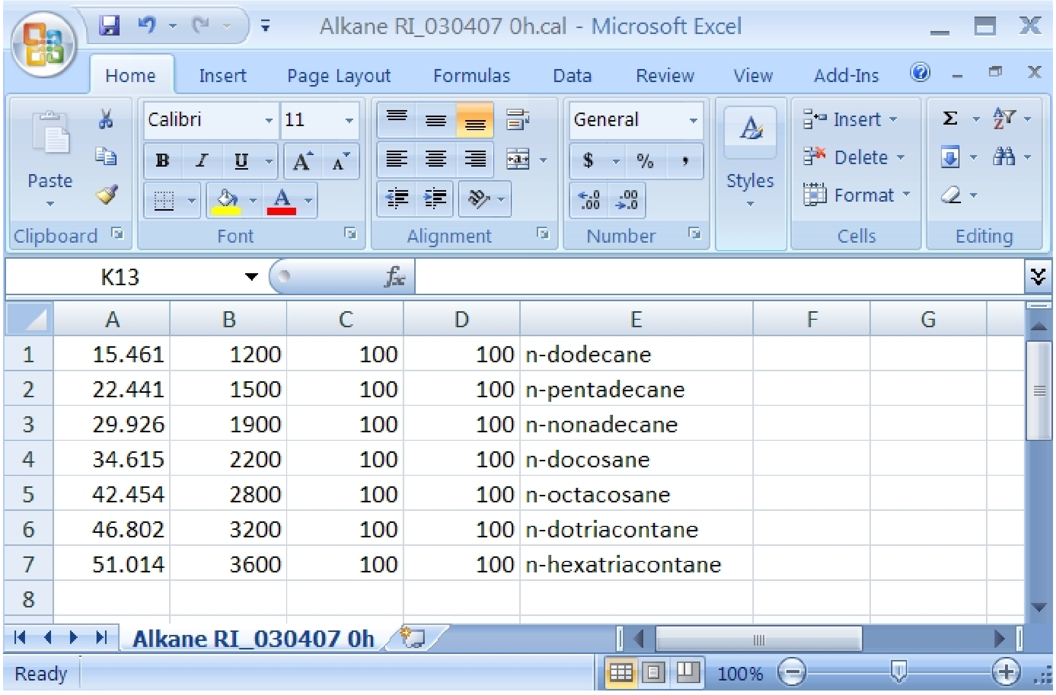

	A	B	C	D	E	F	G
1	15.461	1200	100	100	n-dodecane		
2	22.441	1500	100	100	n-pentadecane		
3	29.926	1900	100	100	n-nonadecane		
4	34.615	2200	100	100	n-docosane		
5	42.454	2800	100	100	n-octacosane		
6	46.802	3200	100	100	n-dotriacontane		
7	51.014	3600	100	100	n-hexatriacontane		
8							

Once you have created the .CAL file, upload it into the experiment folder by FTP. Assuming you have uploaded an appropriate MSRI library to one of your library folders or have used a standard GC/MS protocol that allows you to use one of the public libraries provided by MetabolomeExpress or one of its users, you will now be ready to perform MSRI library matching as soon as your data data import and peak detection is complete.

NOTE: If your GC/MS data was acquired over more than one batch sequence (for example, if 10 runs were done one week and 10 runs done the following week), there may be significant systematic differences in retention time between the different batch sets. Therefore, you will need to create one .CAL file per batch sequence to ensure calibration is always correct. If you want to make direct comparisons between different samples, it is important to run the samples to be compared in the same batch (ie. don't run your treatment samples one week and your control samples the next week, for instance).

1.4.2 Choosing a research area and importing sample information

Once you have uploaded basic sample information in the form of a completed MINIMET.TXT file, you can use the file to generate a functional template METADATA.TXT file appropriate to your research area. MetabolomeExpress provides 18 different metadata validation templates tailored to the unique metadata and ontology requirements of different research fields and different model organisms.

To do this, go to the MetabolomeExpress interface and right click on an experiment folder in the data tree in the Database Navigation panel. Then follow the Import

Sample Information from MINIMET.TXT menu and find your research area in the list of options. See the screenshot below for an example:

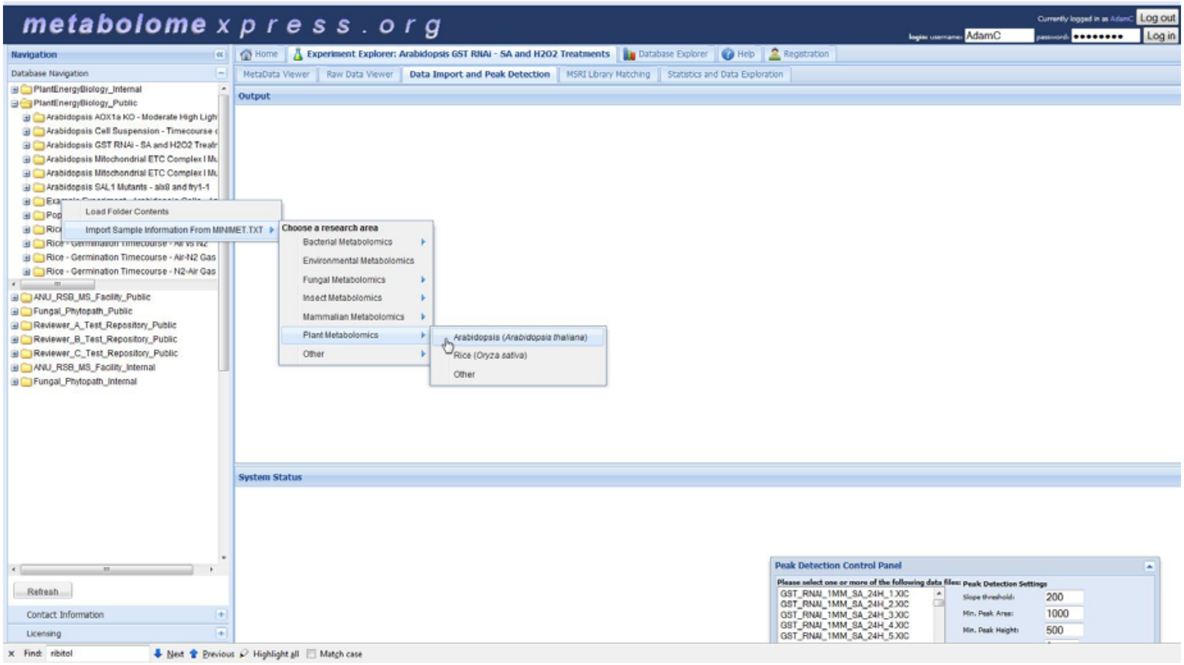

Once you select your research field, you will be asked to confirm that you want to create and new METADATA.TXT file and write over the old one. A backup of the original file (if one existed) will be made under a new file name tagged with the word ‘backup’ and a timestamp.

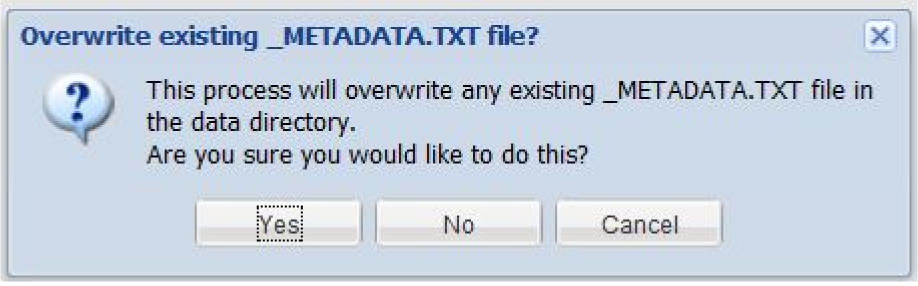

If you click OK, you will be provided with a link from which to download your new METADATA file.

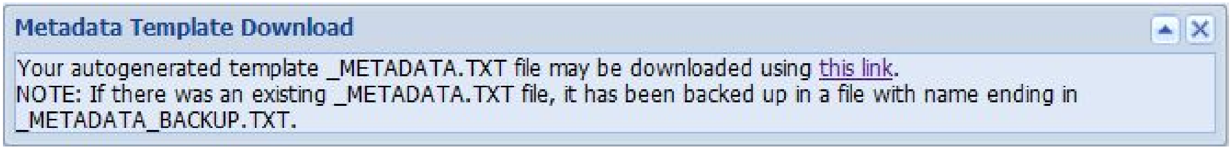

You should open it up in Excel and have a look. You may want to add some details such as Genotype x Environment Class Comparisons or Instrument Batch IDs which may be important later on.

1.5 Building and using custom Mass Spectral and Retention Index (MSRI) libraries

1.5.1 An overview of the MetabolomeExpress MSRI library format

While MetabolomeExpress provides public MSRI libraries built under standard GC/MS operating protocols, many users will want to use their own GC/MS methods and/or MSRI libraries. MetabolomeExpress uses a simple tab-delimited format for MSRI libraries (filename extension '.MSRI', explained shortly) and these may be uploaded into either the 'public' or 'internal' subfolder of the 'libraries' folder present in a user's FTP repository. Libraries added to the 'public' subfolder will be made publicly accessible to anonymous users via the MetabolomeExpress web interface. Libraries added to the 'internal' subfolder will only be accessible to users who are logged in and have permission to access that repository.

The MSRI format in its simplest form is a tab-delimited text file table which may contain any number of columns provided it has a certain minimum set of 5 columns required by MetabolomeExpress. The screenshot below shows an example of a library with the minimum set of columns (ie. 'Name', 'RI', 'Quantifier Ions', 'ID' and 'Mass Spectrum'):

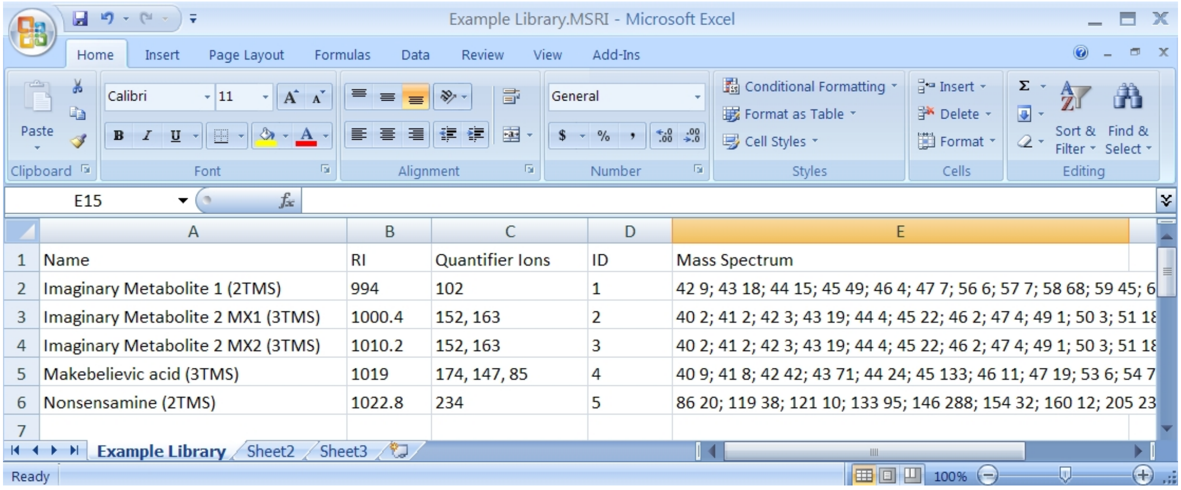

	A	B	C	D	E
1	Name	RI	Quantifier Ions	ID	Mass Spectrum
2	Imaginary Metabolite 1 (2TMS)	994	102	1	42 9; 43 18; 44 15; 45 49; 46 4; 47 7; 56 6; 57 7; 58 68; 59 45; 6
3	Imaginary Metabolite 2 MX1 (3TMS)	1000.4	152, 163	2	40 2; 41 2; 42 3; 43 19; 44 4; 45 22; 46 2; 47 4; 49 1; 50 3; 51 18
4	Imaginary Metabolite 2 MX2 (3TMS)	1010.2	152, 163	3	40 2; 41 2; 42 3; 43 19; 44 4; 45 22; 46 2; 47 4; 49 1; 50 3; 51 18
5	Makebelievic acid (3TMS)	1019	174, 147, 85	4	40 9; 41 8; 42 42; 43 71; 44 24; 45 133; 46 11; 47 19; 53 6; 54 7
6	Nonsensamine (2TMS)	1022.8	234	5	86 20; 119 38; 121 10; 133 95; 146 288; 154 32; 160 12; 205 23
7					

The order of the columns is not important, as long as the column labels are exactly as shown.

The data to put in each column is as follows:

> **Name -** This is the name of the metabolite derivative. It does not have to be unique to a particular entry, so having several entries with the name 'Alanine (2TMS)', for example, would be fine as long as the ID for each entry in the library is unique.
>
> > **NOTE:** One important issue with naming, however, is that certain naming styles allow MetabolomeExpress to derive the name of the underivatised metabolite from the derivative name. Name recognition is case insensitive. The general syntax for naming is:

Common Name of Metabolite [space] *Derivative Information*

where:

Common Name of Metabolite = any commonly used name for the metabolite. MetabolomeExpress has a database of over 100 000 different metabolite synonyms so if you use a common name, it will probably be recognised. You can check whether your metabolite entries are being recognised later using the MetabolomeExpress MSRI Library Manager.

and

Derivative Information = any combination of the following general terms in any order (where ***X*** = any integer or is omitted altogether):

methoxime

methoxyamine

MX***X***

X TMS

XTMS

(***X*** TMS)

(***X***TMS)

X TBS

XTBS

(***X*** TBS)

(***X***TBS)

Peak

EZ Peak ***X***

Peak***X***

Peak ***X***

major

minor

BP

{BP}

{ BP}

derivative

unknown derivative

, unknown derivative

The table below shows a few examples:

Library Entry	Matched Metabolite
alpha-ketoglutarate (2TMS) methoxime	alpha-ketoglutarate
D-Glucose MX1 (5 TMS)	D-Glucose
Alanine (2TMS)	Alanine
Glucose methoxime (5TMS) EZ Peak 1	Glucose

RI - The Kovats retention index of the analyte. Please use only true Kovats RI values.

Quantifier Ions - One or more nominal mass quantifier ions to use, separated by commas.

ID - A unique ID number or string for the library entry. It is recommended that you keep these as short as possible because they will be included in library match annotations and displayed onscreen.

Mass Spectrum - The mass spectrum of the analyte, encoded as a series of m/z:intensity pairs, where each m/z:intensity pair is given as the m/z followed by a space and then the intensity followed by a semicolon and then (optionally) a space.

1.5.2 Creating MSRI libraries from AMDIS .MSL files

How you build your library table is a matter of personal preference. However, we use the freely available deconvolution tool, AMDIS, to build libraries from reference chromatograms and then use the MetabolomeExpress MSRI Library Manager to convert AMDIS .MSL format libraries to MetabolomeExpress .MSRI format libraries

and then use a spreadsheet to fill in the quantifier ion column. It is also possible to convert .MSRI format libraries back into AMDIS .MSL format libraries.

To convert an AMDIS .MSL format library into an .MSRI format library, upload the .MSL file into your 'internal' libraries folder and open the MetabolomeExpress *MSRI Library Manager* in your browser by navigating to *Database Explorer > MSRI Library Manager* in the MetabolomeExpress web interface. You will need to be logged in to see the libraries in your internal libraries folder. Expand your *In-house MSRI Libraries* Folder in the control panel > right click on your .MSL library > left click 'Generate MetabolomeExpress .MSRI Format'.

The screenshot below shows an .MSL format version of the 'Q_MSRI' library (named 'mpimp.msl') from the Max Planck Institute for Molecular Plant Physiology (MPIMP) Golm Metabolome Database being selected for conversion to .MSRI format.

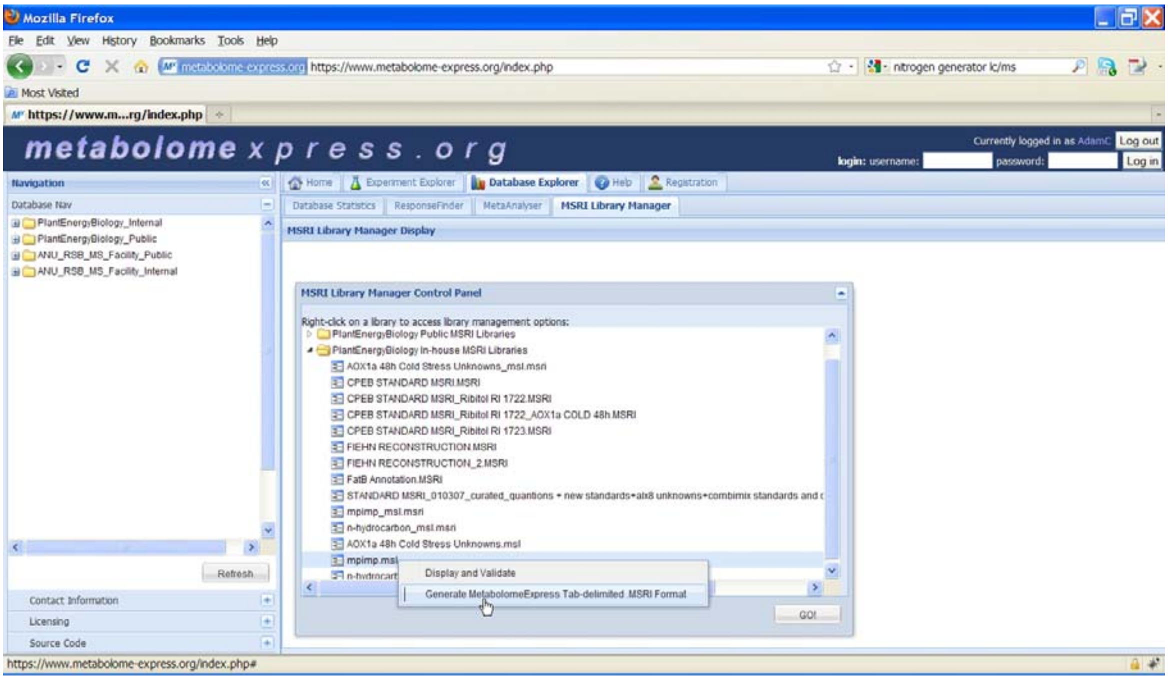

Once a library is selected for conversion, wait a few moments while the library is converted, checked and displayed in the main window of the *MSRI Library Manager* as shown below.

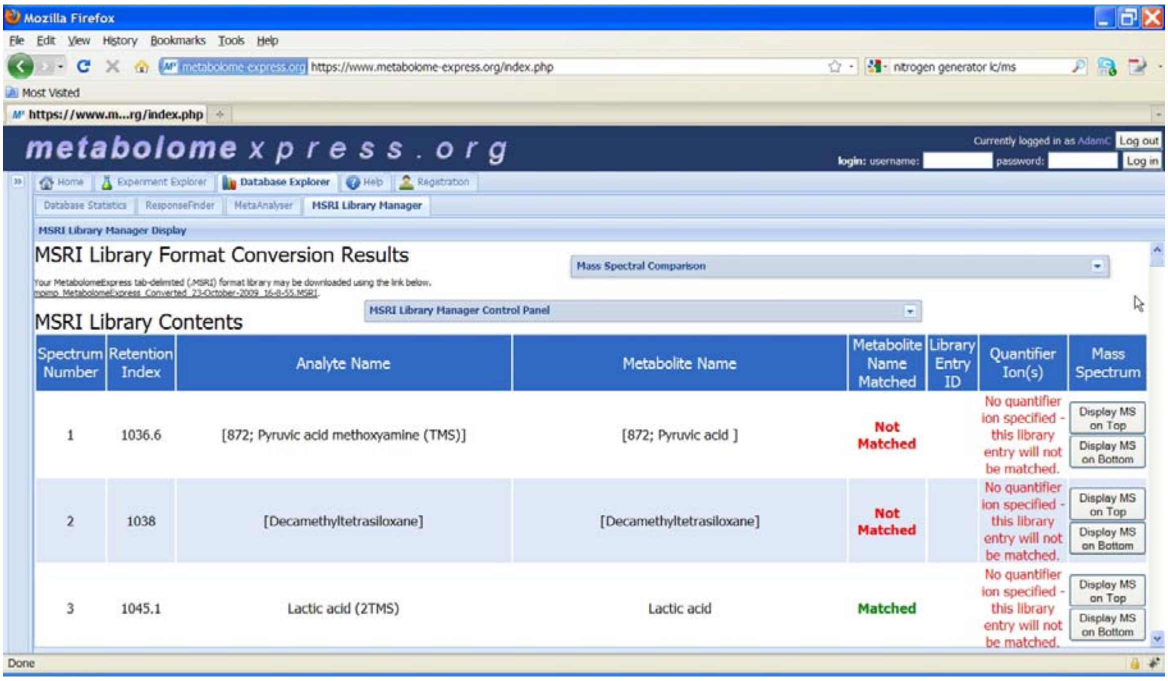

As shown in the screenshot above, the 'Analyte Name' column shows the name of the library entry. The 'Metabolite Name' column shows the metabolite name that the string processing algorithm has derived from the Analyte Name after removing from it all the strings recognised as derivative information. The 'Metabolite Name Matched' column indicates whether the derived metabolite name was found in the MetabolomeExpress Metabolite Name:InChI adapter database. To ensure that library entries for unknown metabolites are never identified as known metabolites, it's a good idea to enclose their names in square brackets as done by the clever people at MPIMP in their library. The 'Library Entry ID' column shows the IDs of the entries, which, in the case of this freshly imported library, have not been set. The 'Quantifier Ion(s)' column shows the quantifier ions specified for each entry. Again, these need to be carefully selected and entered using a spreadsheet. The final column provides buttons that load the mass spectrum of each library entry into either the top or bottom MS display window. You may need to expand the MS Comparison window if you have already collapsed it. This is useful for examining and comparing library spectra. Below, you can see a comparison between the spectra of Lactic acid (2TMS) and Alanine (2TMS) being displayed.

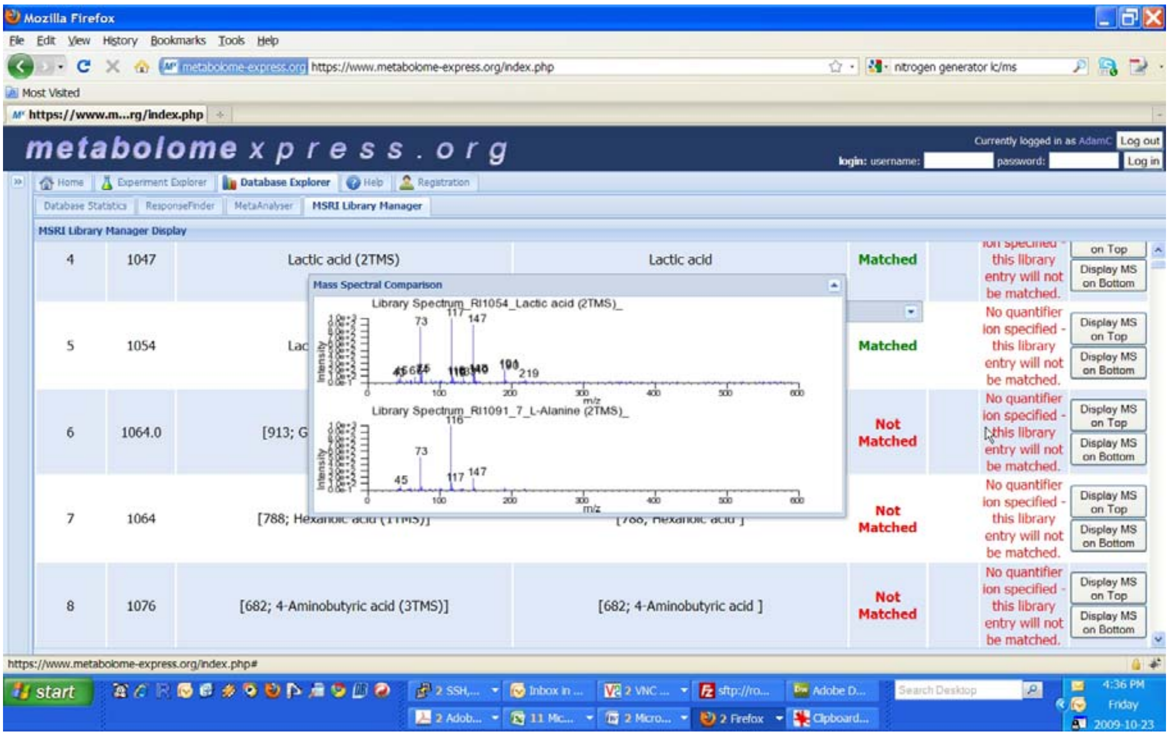

1.5.3 Adding metadata to MSRI libraries

It is possible to extend the basic MSRI library format in two ways. One way is to add additional columns of information to the table. The other way is to add a metadata section to the top of the library file. Adding metadata about how the library was made is essential if it is to be publicy disemminated. To do this, add the following line to the top of the file:

```
///MSRI Library Attributes
```

You may then add three metadata sections to the file (ie. [ADMINISTRATION], [INSTRUMENTAL PARAMETERS] and [DATA PROCESSING PARAMETERS]). The following screenshot shows how these sections are added to the file. Essential lines and cells are highlighted with a light blue background and bold text (this is for illustration purposes only - no formatting is stored in the tab-delimited file). The fields shown below are recommended but the actual field names and their values are totally flexible and you may add as many fields to each section as you like.

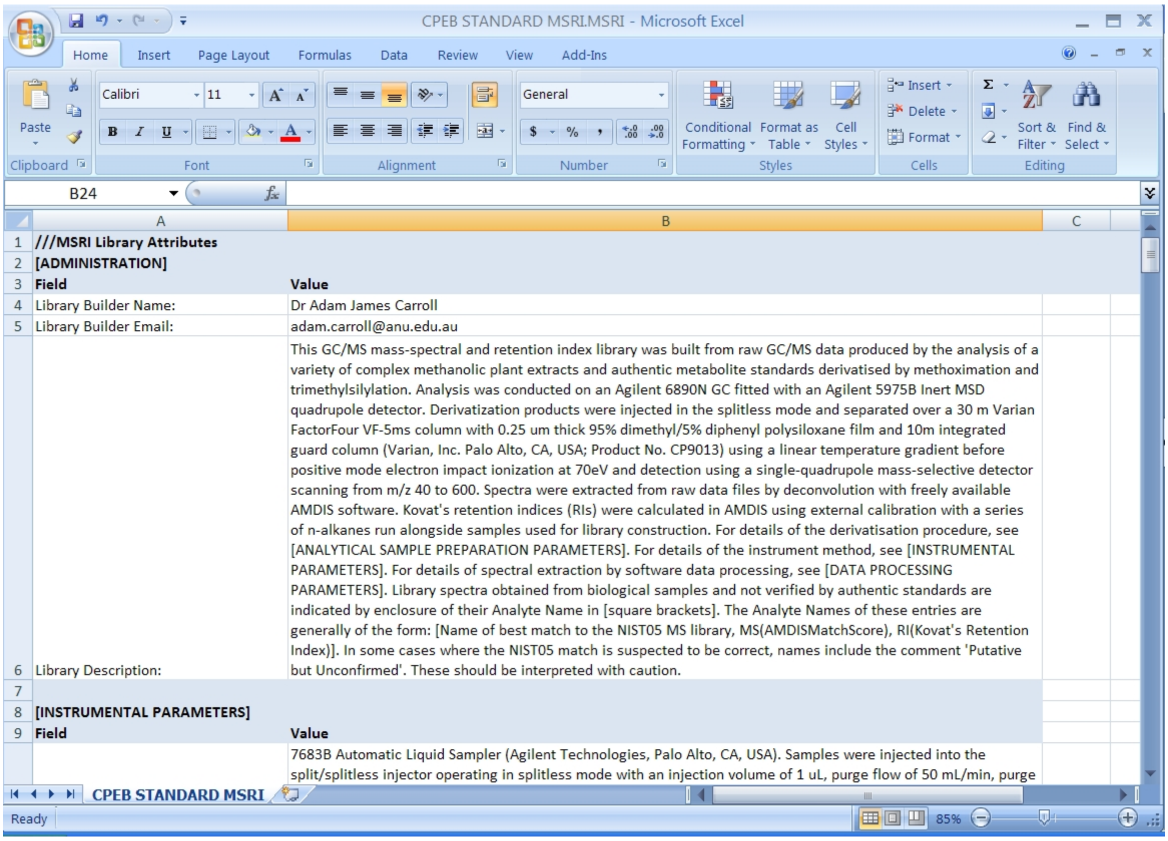

	A	B
1	**///MSRI Library Attributes**	
2	**[ADMINISTRATION]**	
3	**Field**	**Value**
4	Library Builder Name:	Dr Adam James Carroll
5	Library Builder Email:	adam.carroll@anu.edu.au
6	Library Description:	This GC/MS mass-spectral and retention index library was built from raw GC/MS data produced by the analysis of a variety of complex methanolic plant extracts and authentic metabolite standards derivatised by methoximation and trimethylsilylation. Analysis was conducted on an Agilent 6890N GC fitted with an Agilent 5975B Inert MSD quadrupole detector. Derivatization products were injected in the splitless mode and separated over a 30 m Varian FactorFour VF-5ms column with 0.25 um thick 95% dimethyl/5% diphenyl polysiloxane film and 10m integrated guard column (Varian, Inc. Palo Alto, CA, USA; Product No. CP9013) using a linear temperature gradient before positive mode electron impact ionization at 70eV and detection using a single-quadrupole mass-selective detector scanning from m/z 40 to 600. Spectra were extracted from raw data files by deconvolution with freely available AMDIS software. Kovat's retention indices (RIs) were calculated in AMDIS using external calibration with a series of n-alkanes run alongside samples used for library construction. For details of the derivatisation procedure, see [ANALYTICAL SAMPLE PREPARATION PARAMETERS]. For details of the instrument method, see [INSTRUMENTAL PARAMETERS]. For details of spectral extraction by software data processing, see [DATA PROCESSING PARAMETERS]. Library spectra obtained from biological samples and not verified by authentic standards are indicated by enclosure of their Analyte Name in [square brackets]. The Analyte Names of these entries are generally of the form: [Name of best match to the NIST05 MS library, MS(AMDISMatchScore), RI(Kovat's Retention Index)]. In some cases where the NIST05 match is suspected to be correct, names include the comment 'Putative but Unconfirmed'. These should be interpreted with caution.
7		
8	**[INSTRUMENTAL PARAMETERS]**	
9	**Field**	**Value**
		7683B Automatic Liquid Sampler (Agilent Technologies, Palo Alto, CA, USA). Samples were injected into the split/splitless injector operating in splitless mode with an injection volume of 1 uL, purge flow of 50 mL/min, purge

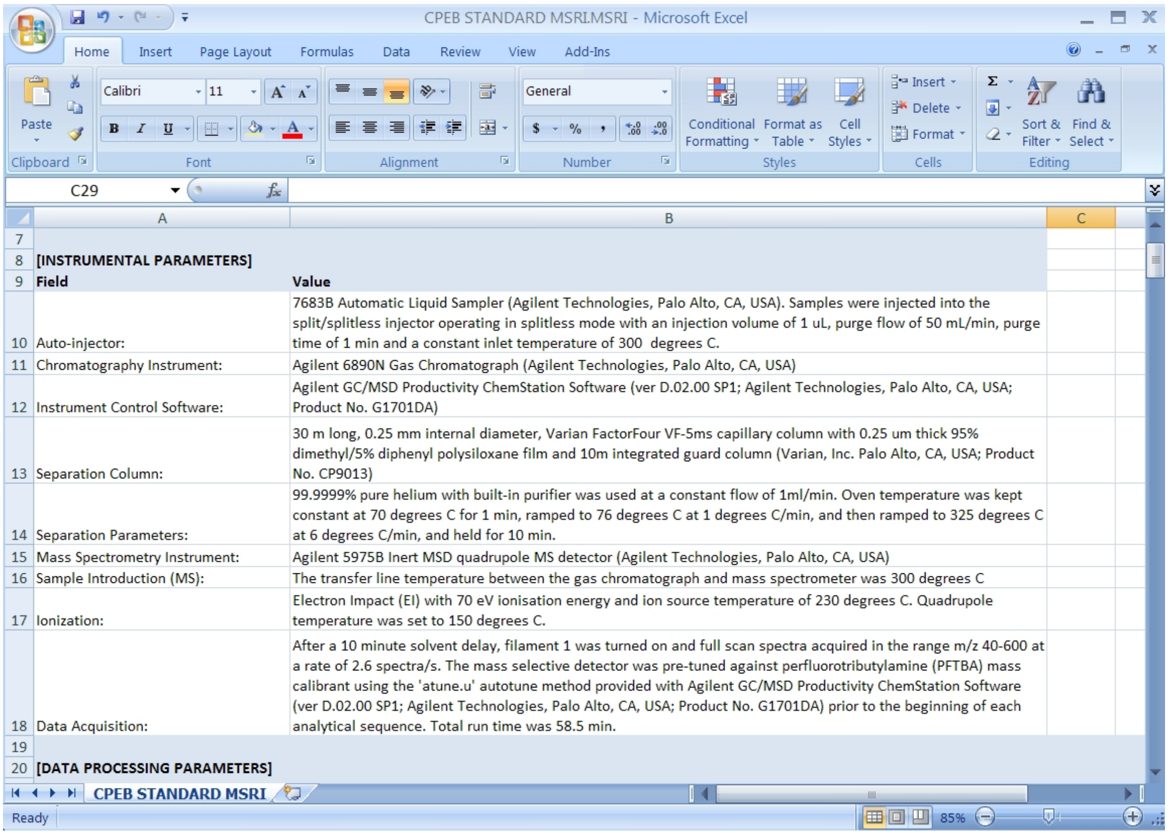

	A	B
7		
8	**[INSTRUMENTAL PARAMETERS]**	
9	**Field**	**Value**
10	Auto-injector:	7683B Automatic Liquid Sampler (Agilent Technologies, Palo Alto, CA, USA). Samples were injected into the split/splitless injector operating in splitless mode with an injection volume of 1 uL, purge flow of 50 mL/min, purge time of 1 min and a constant inlet temperature of 300 degrees C.
11	Chromatography Instrument:	Agilent 6890N Gas Chromatograph (Agilent Technologies, Palo Alto, CA, USA)
12	Instrument Control Software:	Agilent GC/MSD Productivity ChemStation Software (ver D.02.00 SP1; Agilent Technologies, Palo Alto, CA, USA; Product No. G1701DA)
13	Separation Column:	30 m long, 0.25 mm internal diameter, Varian FactorFour VF-5ms capillary column with 0.25 um thick 95% dimethyl/5% diphenyl polysiloxane film and 10m integrated guard column (Varian, Inc. Palo Alto, CA, USA; Product No. CP9013)
14	Separation Parameters:	99.9999% pure helium with built-in purifier was used at a constant flow of 1ml/min. Oven temperature was kept constant at 70 degrees C for 1 min, ramped to 76 degrees C at 1 degrees C/min, and then ramped to 325 degrees C at 6 degrees C/min, and held for 10 min.
15	Mass Spectrometry Instrument:	Agilent 5975B Inert MSD quadrupole MS detector (Agilent Technologies, Palo Alto, CA, USA)
16	Sample Introduction (MS):	The transfer line temperature between the gas chromatograph and mass spectrometer was 300 degrees C
17	Ionization:	Electron Impact (EI) with 70 eV ionisation energy and ion source temperature of 230 degrees C. Quadrupole temperature was set to 150 degrees C.
18	Data Acquisition:	After a 10 minute solvent delay, filament 1 was turned on and full scan spectra acquired in the range m/z 40-600 at a rate of 2.6 spectra/s. The mass selective detector was pre-tuned against perfluorotributylamine (PFTBA) mass calibrant using the 'atune.u' autotune method provided with Agilent GC/MSD Productivity ChemStation Software (ver D.02.00 SP1; Agilent Technologies, Palo Alto, CA, USA; Product No. G1701DA) prior to the beginning of each analytical sequence. Total run time was 58.5 min.
19		
20	**[DATA PROCESSING PARAMETERS]**	

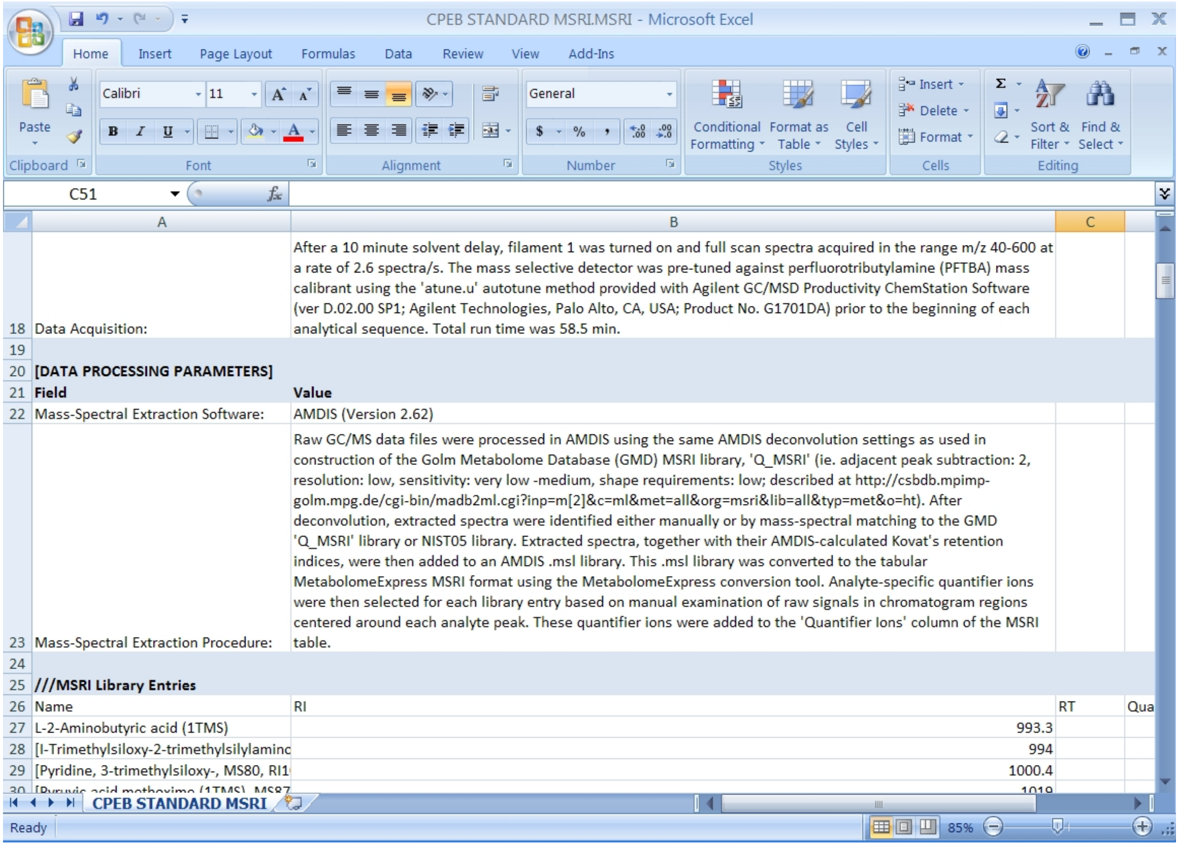

Note above how there is a blank line and then the line '`///MSRI Library Entries`' before the main table starts.

1.5.4 Displaying and Validating MSRI Libraries with MSRI Library Manager

The contents of an existing MSRI library may be viewed at any time by right-clicking on the library in the *MSRI Library Manager* and selecting 'Display and Validate'. This will display any metadata in the library and generate a validation and review table as shown in the earlier screenshot. It will also provide you with a hyperlink to the MSRI library file and automatically generate a template analyte annotations table which annotates each library entry with its matched standard underivatised metabolite name, its InChI structure code and its chemical class. These tables also provide four boolean [ie. TRUE (1) or FALSE (0)] columns that allow you to specify whether each library entry is: 1. to be used as a quantifier peak for its corresponding metabolite; 2. of unknown structure; 3. an internal standard; or 4. an artefact analyte of non-biological origin. These tables are important for proper filtering and display of data for statistical analysis. Instructions for their use are given below.

1.5.5 Using Analyte Annotations Tables to customise data filtering

If using custom MSRI libraries, most of the tools in the Statistics and Data Exploration module of Experiment Explorer require that an analyte annotations table file containing annotation information for the entries in those MSRI libraries be present either in the 'libraries' FTP folder of the repository containing the dataset of interest or in the actual folder of the individual experiment. To generate a template analyte annotations table file from an MSRI library, 'Display and Validate' that MSRI library in the MSRI Library

Manager as described above. You may then scroll down and use the hyperlink to download the automatically generated analyte annotations file already containing the following automatically assigned annotations for each library entry:

Metabolite Name: The common name of the underivatised metabolite matched to the Analyte Name of the MSRI Library entry [most of these common names are the same as those in the Human Metabolome Database]

InChI Identifier: This is the unambiguous structural identifier string of the underivatised metabolite corresponding to the library entry (if it is known)

Chemical Class: This is the chemical class of the underivatised metabolite (eg. Amino Acids) [most of these classes are as defined in the HMDB].

is_unknown_structure: This is a boolean with value of either 1 (TRUE) or 0 (FALSE). Set to 1 if the analyte is of unknown structure or has not been verified with authentic standards. Library entries not automatically matched to known metabolites will be automatically set to 1.

is_quant_peak: This is a boolean with value of either 1 (TRUE) or 0 (FALSE). Set to 1 if you wish peak areas for this library entry to be considered as representative of levels of its corresponding metabolite. Set to 0 if you want to exclude values for this peak from quantitative analyses. This is useful for preventing highly variable or unreliable metabolite derivatives from influencing results.

is_artefact: This is a boolean with value of either 1 (TRUE) or 0 (FALSE). Set to 1 if the library entry represents an analytical artefact analyte of substantially non-biological origin. Otherwise, set to 0. Quantitative data thus annotated as corresponding to artefacts will be automatically removed from data matrices prior to multivariate analysis.

is_internal_standard: This is a boolean with value of either 1 (TRUE) or 0 (FALSE). Set to 1 if the library entry represents an internal standard analyte of non-biological origin (eg. n-alkanes, FAMES, ribitol). Otherwise, set to 0. Quantitative data thus annotated as corresponding to internal standards will be automatically removed from data matrices prior to multivariate analysis.

The screenshot below shows some example rows for different types of analytes.

Analyte Name	Metabolite Name	InChI Identifier	Chemical Class	is_unknown_structure	is_quant_peak	is_artefact	is_internal_std
3,4-Dihydroxycinnamic acid (3TMS)	3,4-dihydroxycinnamic acid	InChI=1/C9H8O4/c10-7-3-1-6(5-8(7)11)2-4-9(12)13/h1-5,10-11H,(H,12,13)/f/h12H	Aromatic Acids	0	1	0	0
4-Aminobutyric acid (3TMS)	Gamma-Aminobutyric acid	InChI=1/C4H9NO2/c5-3-1-2-4(6)7/h1-3,5H2,(H,6,7)	Amino Acids	0	1	0	0
6-Phosphogluconic acid (7TMS)	6-Phosphogluconic acid	InChI=1/C6H13O10P/c7-2(1-16-17(13,14)15)3(8)4(9)5(10)6(11)12/h2-5,7-10H,1H2,(H,11,12)(H2,13,14,15)/t2-,3-,4+,5-/m1/s1	Sugar Phosphates	0	1	0	0
Adenine (2TMS)	Adenine	InChI=1/C5H5N5/c6-4-3-5(9-1-7-3)10-2-8-4/h1-2H,(H3,6,7,8,9,10)	Purines and Purine Derivatives	0	1	0	0
n-docosane	n-docosane	InChI=1/C22H46/c1-3-5-7-9-11-13-15-17-19-21-22-20-18-16-14-12-10-8-6-4-2/h3-22H2,1-2H3	Alkanes	0	1	0	1
Ribitol (5TMS)	Ribitol	InChI=1/C5H12O5/c6-1-3(8)5(10)4(9)2-7/h3-10H,1-2H2/t3-,4+,5-	Alcohols and Polyols	0	1	0	1
[Pentasiloxane, dodecamethyl-, MS92, RI1152.7]	Unknown	Unknown	Unknown	1	1	1	0
[Unknown Probable Disaccharide, RI2748.9]	Unknown	Unknown	Unknown	1	1	0	0

Once you have appropriately edited your Analyte Annotations Table, save it as a tab-delimited text file and place it either in your 'libraries' FTP folder or in the folder of the experiment you wish to apply the annotations to. If you put it in the 'libraries' folder, it will be applied to all experiments in your repository except where locally overridden by an analyte annotations table in an experiment folder. A file is recognised as an analyte annotations table when its file name starts with the string 'analyte_annotations_table_'. If more than one analyte annotations table is detected in a folder, then the one with the highest alphanumeric ranking is used (eg. a file named 'analyte_anotations_table_2010.txt' would be used in the presence of another file named 'analyte_anotations_table_2009.txt').

1.6 Supported Data Formats (including example files)

1.6.1 The main MetabolomeExpress metadata exchange format (*_METADATA.TXT)

For public dissemination, it is essential that metabolomics datasets include sufficient metadata to allow other researchers to understand the biological and technical origins of the data in enough detail to be able to reproduce essentially the same results. The Metabolomics Standards Initiative (MSI) has outlined minimal reporting standards and guidelines for metabolomics metadata reporting and these guided the design of a simple metadata exchange format for use in MetabolomeExpress. The MetabolomeExpress metadata exchange format is tab-delimited and designed to be readable by both humans and computers whilst retaining the flexibility and extensibility required in the ever-changing world of data-reporting standards. The file is divided into seven main subsections as indicated by the figure below:

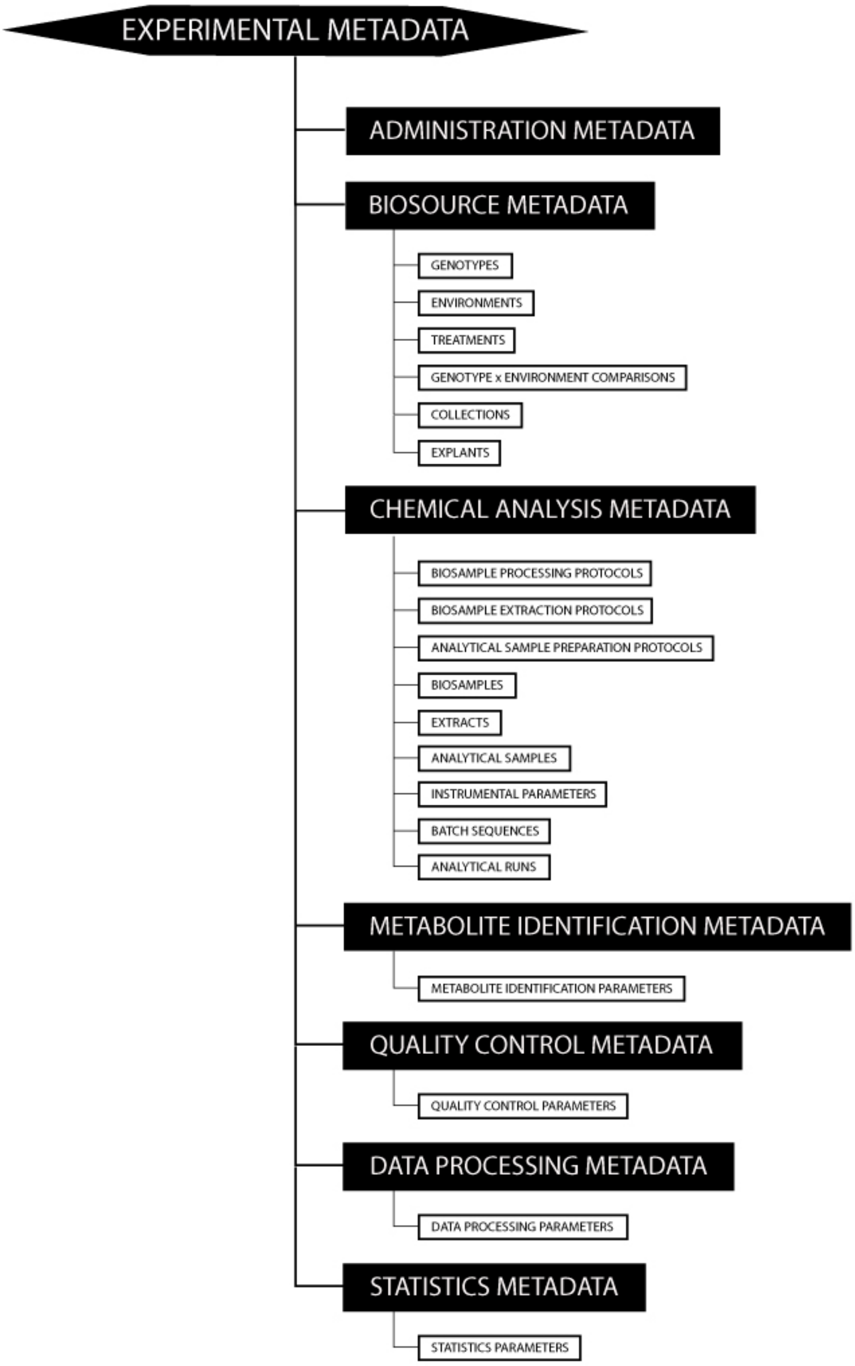

The Administration, Biosource, and Chemical Analysis metadata sections are the only ones essential for MetabolomeExpress processing. Their structures and content are shown below:

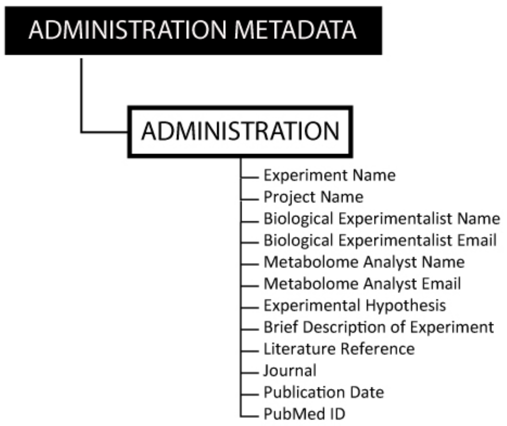
ADMINISTRATION METADATA
ADMINISTRATION
Experiment Name
Project Name
Biological Experimentalist Name
Biological Experimentalist Email
Metabolome Analyst Name
Metabolome Analyst Email
Experimental Hypothesis
Brief Description of Experiment
Literature Reference
Journal
Publication Date
PubMed ID

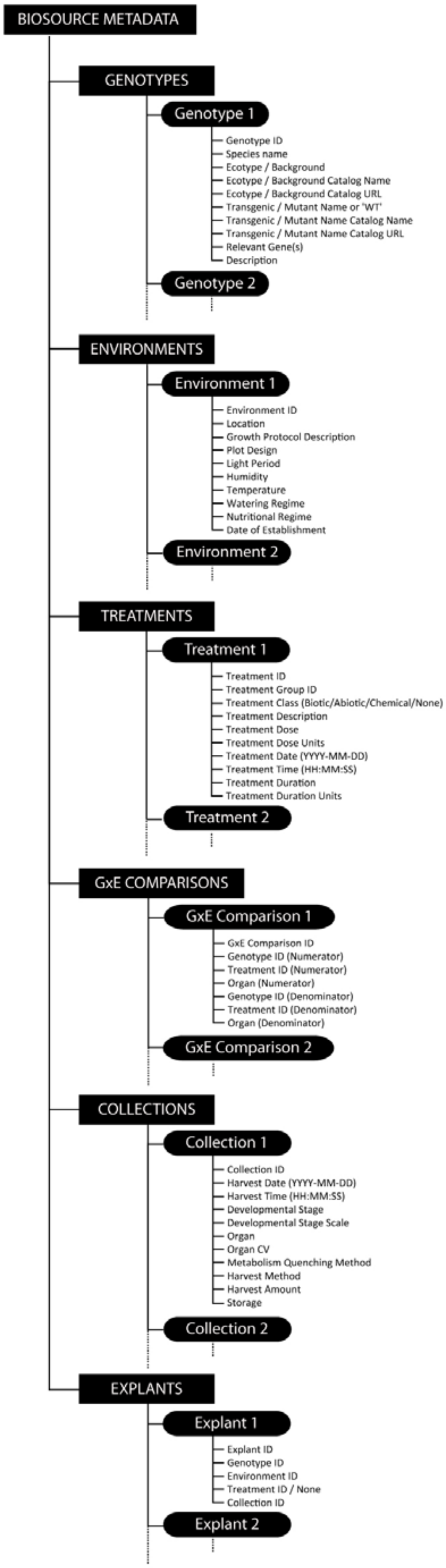
BIOSOURCE METADATA
GENOTYPES
Genotype 1
Genotype ID
Species name
Ecotype / Background
Ecotype / Background Catalog Name
Ecotype / Background Catalog URL
Transgenic / Mutant Name or 'WT'
Transgenic / Mutant Name Catalog Name
Transgenic / Mutant Name Catalog URL
Relevant Gene(s)
Description
Genotype 2
ENVIRONMENTS
Environment 1
Environment ID
Location
Growth Protocol Description
Plot Design
Light Period
Humidity
Temperature
Watering Regime
Nutritional Regime
Date of Establishment
Environment 2
TREATMENTS
Treatment 1
Treatment ID
Treatment Group ID
Treatment Class (Biotic/Abiotic/Chemical/None)
Treatment Description
Treatment Dose
Treatment Dose Units
Treatment Date (YYYY-MM-DD)
Treatment Time (HH:MM:SS)
Treatment Duration
Treatment Duration Units
Treatment 2
GxE COMPARISONS
GxE Comparison 1
GxE Comparison ID
Genotype ID (Numerator)
Treatment ID (Numerator)
Organ (Numerator)
Genotype ID (Denominator)
Treatment ID (Denominator)
Organ (Denominator)
GxE Comparison 2
COLLECTIONS
Collection 1
Collection ID
Harvest Date (YYYY-MM-DD)
Harvest Time (HH:MM:SS)
Developmental Stage
Developmental Stage Scale
Organ
Organ CV
Metabolism Quenching Method
Harvest Method
Harvest Amount
Storage
Collection 2
EXPLANTS
Explant 1
Explant ID
Genotype ID
Environment ID
Treatment ID / None
Collection ID
Explant 2

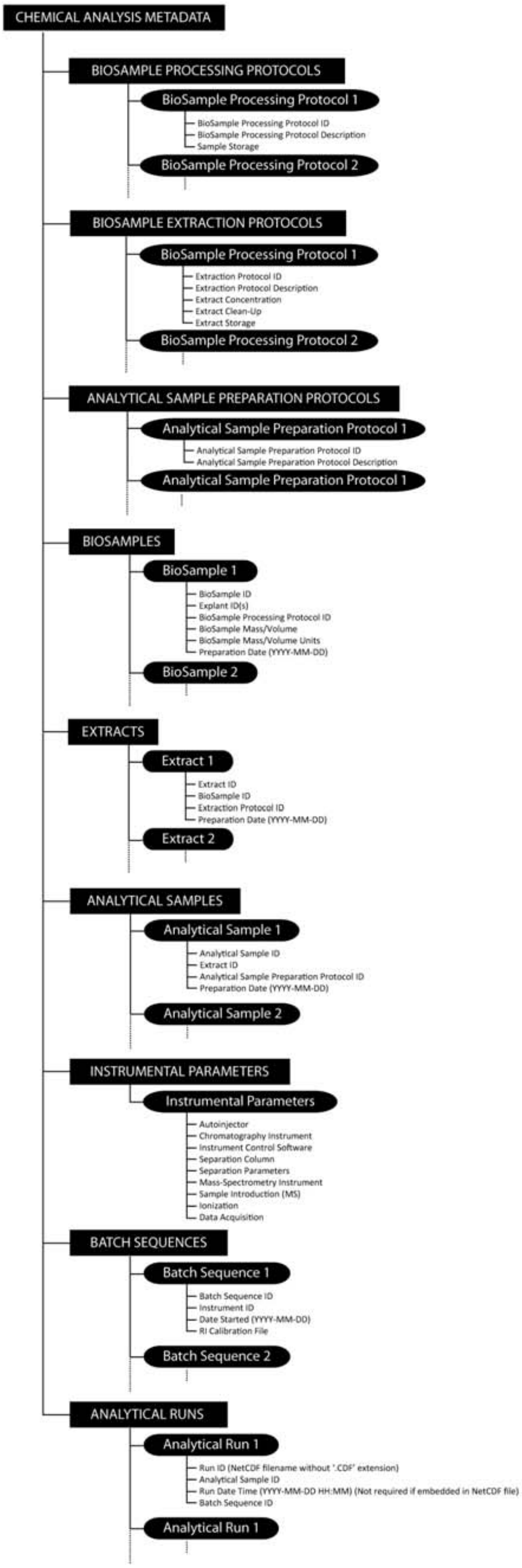
CHEMICAL ANALYSIS METADATA
BIOSAMPLE PROCESSING PROTOCOLS
BioSample Processing Protocol 1
BioSample Processing Protocol ID
BioSample Processing Protocol Description
Sample Storage
BioSample Processing Protocol 2
BIOSAMPLE EXTRACTION PROTOCOLS
BioSample Processing Protocol 1
Extraction Protocol ID
Extraction Protocol Description
Extract Concentration
Extract Clean-Up
Extract Storage
BioSample Processing Protocol 2
ANALYTICAL SAMPLE PREPARATION PROTOCOLS
Analytical Sample Preparation Protocol 1
Analytical Sample Preparation Protocol ID
Analytical Sample Preparation Protocol Description
Analytical Sample Preparation Protocol 1
BIOSAMPLES
BioSample 1
BioSample ID
Explant ID(s)
BioSample Processing Protocol ID
BioSample Mass/Volume
BioSample Mass/Volume Units
Preparation Date (YYYY-MM-DD)
BioSample 2
EXTRACTS
Extract 1
Extract ID
BioSample ID
Extraction Protocol ID
Preparation Date (YYYY-MM-DD)
Extract 2
ANALYTICAL SAMPLES
Analytical Sample 1
Analytical Sample ID
Extract ID
Analytical Sample Preparation Protocol ID
Preparation Date (YYYY-MM-DD)
Analytical Sample 2
INSTRUMENTAL PARAMETERS
Instrumental Parameters
Autoinjector
Chromatography Instrument
Instrument Control Software
Separation Column
Separation Parameters
Mass-Spectrometry Instrument
Sample Introduction (MS)
Ionization
Data Acquisition
BATCH SEQUENCES
Batch Sequence 1
Batch Sequence ID
Instrument ID
Date Started (YYYY-MM-DD)
RI Calibration File
Batch Sequence 2
ANALYTICAL RUNS
Analytical Run 1
Run ID (NetCDF filename without '.CDF' extension)
Analytical Sample ID
Run Date Time (YYYY-MM-DD HH:MM) (Not required if embedded in NetCDF file)
Batch Sequence ID
Analytical Run 1

NOTE: The structure shown above was designed for plant metabolomics. MetabolomeExpress support customised field sets and validation schemas for 18 different research areas. To make sure your metadata file passes its corresponding validation test, you will need to have read the section in this manual on interpreting validation templates. You can download the latest validation templates from the Database Explorer module.

The core format structure of the file allows a data file (ie. an 'Analytical Run') to be traced back through the sample preparation workflow to the original biological tissue collection. There are fields to describe the genotype of the harvested organism as well as the growth environment and experimental treatment applied to that organism. In addition, there are fields to describe sample preparation and analytical protocols. New fields may be added to the file without interfering with its use by MetabolomeExpress, which only uses certain core fields for data processing. Probably the best way to understand the format is to read the section in this manual on interpreting validation templates. You could also download and examine the _METADATA.txt files from public experiments in the repository (using the Database Navigation panel on the left of the MetabolomeExpress interface).

1.6.2 Raw GC/MS data: NetCDF and the MetabolomeExpress eXtracted Ion Chromatogram (.XIC) format

The primary raw GC/MS data format used by MetabolomeExpress is the open standard NetCDF/AIA/ANDI format (*.CDF). These may be exported from most instrument manufacturer’s data processing software. We have successfully tested MetabolomeExpress with GC/Quadrupole MS NetCDF files exported from Agilent's ChemStation software and GC/TOF MS files exported from LECO's ChromaTOF software. Slight differences do exist between the structures of CDF files exported from different types of instruments. Please contact us if you have any problems with your files and we will fix them.

Before you can work with your raw data in MetabolomeExpress, you must import your CDF files through the generation, for each CDF file, a corresponding file in the custom MetabolomeExpress eXtracted Ion Chromatogram (*.XIC) binary format. Unlike NetCDF files which are indexed by scan number, XIC files are indexed by m/z channel. Therefore, MetabolomeExpress rapidly retrieves scans of interest from NetCDF files and rapidly retrieves chromatograms of interest from XIC files. Details on how to import raw data files are given in the section in this manual called **Data import and peak detection** (registered users only).

1.6.3 Peak list tables (.PEAKLIST)

A peak list table (file extension ".PEAKLIST") contains information about all the extracted ion chromatogram (EIC) peaks (the signal peaks in each nominal mass m/z channel) in a given GC/MS chromatogram. Peak lists are simply tab-delimited tables with the following columns:

m/z: the integer m/z value of the EIC peak

Apex Time: The retention time of the peak apex in minutes.

Integration Start Time: The retention time of the start of the peak (ie. the point at which the rising signal first breaks threshold) in minutes

Integration End Time: The retention time at which the end of the peak is reached (in minutes).

Total Peak Area: The total area under the peak from start to finish (in arbitrary peak area units).

Peak Height: The height of the signal at the peak apex (in arbitrary peak area units).

Peak Start Intensity: The height of the signal at the peak start retention time (in arbitrary peak area units).

Peak End Intensity: The height of the signal at the peak end retention time (in arbitrary peak area units).

Peak Purity Factor: The ratio of the total peak area to the area lying under the lowest integration point.

Peak Base Area: The total area lying under the lowest integration point.

Number of Scans: The total number of scans between the peak start retention time and the peak end retention time.

Peak Start Scan Number: The scan number at the peak start retention time.

Peak End Scan Number: The scan number at the peak end retention time.

If you include these headers, exactly as written, at the top of the columns in your peaklist file, MetabolomeExpress will recognise them and you may have the columns in any order. If you omit the headers, MetabolomeExpress will assume you have used the default column ordering (ie. the list order of the columns listed above). The MetabolomeExpress PeakFinder algorithm automatically outputs peak lists with these headers. For a peak list to be linked to a chromatogram, it MUST be named with the same filename as the NetCDF file from which it was derived (eg. the peak list file corresponding to the NetCDF file called '20091510_Wildtype_1.CDF' must be named '20091510_Wildtype_1.PEAKLIST').

1.6.4 MSRI library matching report tables (.MATCHREPORT)

MetabolomeExpress library match report tables are used to annotate chromatograms in the Raw Data Viewer and also to construct data matrices using the ‘Match Report to Data Matrix’ tool of the Statistics and Data Exploration component of the Experiment Explorer. The only requirement for the naming of library match reports is that they end with the file extension '.MATCHREPORT'. This enables them to be recognised as library match reports by MetabolomeExpress. The file format for library match reports is a tab-delimited table with the following columns (each row representing a single MSRI library match):

Datafile: the name of the peak list file that was processed to generate this library match (without any path information).

Library Hit Name: The name of the library hit. The MetabolomeExpress MSRI Library Matching algorithm outputs the library hit name in the following syntax (variables displayed in italics, constants displayed in bold): *Name of Library Entry*_**ID***ID of Library Entry*_**RI***RI of Library Entry*_**MZ***m/z of Quantifier Ion*. **NOTE:** This syntax is important, so be sure to use it if building your own library match reports using third party software.

Intensity: The total peak area of the quantifier ion in the matched peak (in arbitrary peak area units).

RT (Apex): The retention time at the quantifier ion peak apex (in minutes).

RT (Start): The retention time at the integration start point of the quantifier ion peak (in minutes).

RT (End): The retention time at the integration end point of the quantifier ion peak (in minutes).

RI (Apex): The *Kovats* retention index at the apex of the peak.

Delta RI: The retention index error of the quantifier ion peak (ie. Observed RI - Expected RI).

Coverage: The percentage of ion signals present in the library spectrum that are present in the extracted MST

Match Details: A string providing details of the library match such as score/quality etc. The MetabolomeExpress MSRI Library Matching alorithm provides information about the number of ion signals in the extracted MST that show the expected intensity ratio with respect to the quantifier ion (within the given % tolerance) as well as the average deviation of all ion intensities from their expected ratios.

1.6.5 Data matrices (.mzrtMATRIX)

The main data matrix format used by MetabolomeExpress has the file extension '.mzrtMATRIX'. It is a tab-delimited format arranged with metabolite signals in rows and runs/samples in columns. There are a number of column header rows at the top of the table containing useful metadata about runs/samples and a number of row header columns containing information about metabolite signals.

Column header rows include:

Data File: The name of the .PEAKLIST file (without path information) representing the sample in that column

Tissue Mass / Volume: The mass or volume of tissue/fluid that was extracted to produce the analytical sample

Genotype ID: The Genotype ID of the organism that was analysed (as given in the _METADATA.TXT file for the experiment).

Organ: The standard name of the organ/tissue/biomaterial type that was taken from the organism and processed to produce the sample.

Treatment ID: The Treatment ID of the experimental treatment applied to organism that was analysed.

Treatment Duration: The treatment duration applied to the organism that was analysed.

Treatment Dosage: The treatment dosage applied to the organism that was analysed.

Replicate: The replicate number of the sample. For a given genotype/treatment/organ combination, replicates are numbered 1-*x* where *x* is the number of replicates.

Row header columns include:

Analyte Signal ID: The library match annotation as given in the Library Hit Name column of the MSRI Library Match Report. It must be given in the following syntax (variables displayed in italics, constants displayed in bold): *Name of Library Entry*_**ID***ID of Library Entry*_**RI***RI of Library Entry*_**MZ***m/z of Quantifier Ion*.

Average Retention Time (min): The average retention time of the matched quantifier ion signal across the entire row of the table.

Average Retention Index (Kovats): The average Kovats retention index of the matched quantifier ion signal across the entire row of the table.

m/z: The m/z of the quantifier ion used to quantify the analyte represented by this row.

The layout of the mzrtMATRIX format is shown in the screenshot below:

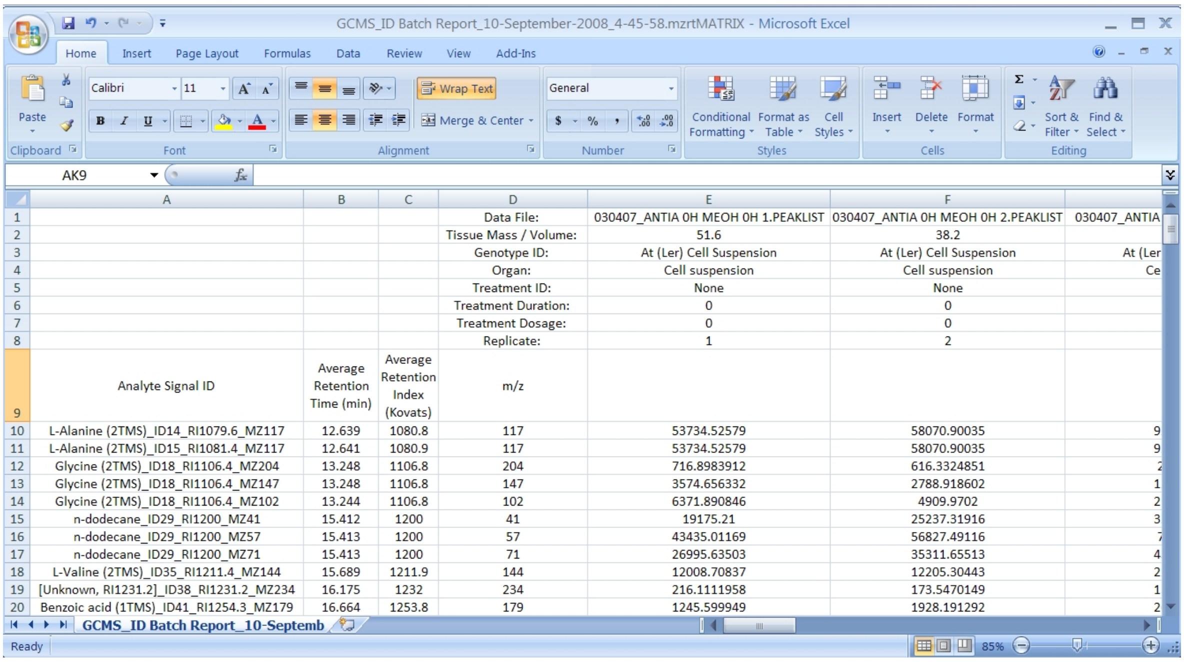

GCMS_ID Batch Report_10-September-2008_4-45-58.mzrtMATRIX - Microsoft Excel

	A	B	C	D	E	F	
1				Data File:	030407_ANTIA 0H MEOH 0H 1.PEAKLIST	030407_ANTIA 0H MEOH 0H 2.PEAKLIST	030407_ANTIA
2				Tissue Mass / Volume:	51.6	38.2	
3				Genotype ID:	At (Ler) Cell Suspension	At (Ler) Cell Suspension	At (Ler
4				Organ:	Cell suspension	Cell suspension	Ce
5				Treatment ID:	None	None	
6				Treatment Duration:	0	0	
7				Treatment Dosage:	0	0	
8				Replicate:	1	2	
9	Analyte Signal ID	Average Retention Time (min)	Average Retention Index (Kovats)	m/z			
10	L-Alanine (2TMS)_ID14_RI1079.6_MZ117	12.639	1080.8	117	53734.52579	58070.90035	9
11	L-Alanine (2TMS)_ID15_RI1081.4_MZ117	12.641	1080.9	117	53734.52579	58070.90035	9
12	Glycine (2TMS)_ID18_RI1106.4_MZ204	13.248	1106.8	204	716.8983912	616.3324851	2
13	Glycine (2TMS)_ID18_RI1106.4_MZ147	13.248	1106.8	147	3574.656332	2788.918602	1
14	Glycine (2TMS)_ID18_RI1106.4_MZ102	13.244	1106.8	102	6371.890846	4909.9702	2
15	n-dodecane_ID29_RI1200_MZ41	15.412	1200	41	19175.21	25237.31916	3
16	n-dodecane_ID29_RI1200_MZ57	15.413	1200	57	43435.01169	56827.49116	7
17	n-dodecane_ID29_RI1200_MZ71	15.413	1200	71	26995.63503	35311.65513	4
18	L-Valine (2TMS)_ID35_RI1211.4_MZ144	15.689	1211.9	144	12008.70837	12205.30443	2
19	[Unknown, RI1231.2]_ID38_RI1231.2_MZ234	16.175	1232	234	216.1111958	173.5470149	1
20	Benzoic acid (1TMS)_ID41_RI1254.3_MZ179	16.664	1253.8	179	1245.599949	1928.191292	2

2 Re-analysis of publicly disseminated data using *Database Explorer*

The *Database Explorer* module provides tools to interact with data in the MetabolomeExpress database of metabolite response statistics. It currently contains four sub-modules: *Database Statistics*, *ResponseFinder*, *MetaAnalyser* and *MSRI Library Manager*. The latter has been described above in the section 'Building and using custom MSRI libraries'. The other three are described below.

2.1 Getting an overview of the database with the *Database Statistics* panel

The *Database Statistics* panel provides a summary of the current contents of the MetabolomeExpress database of metabolite response statistics. It currently displays two tables: one summarising the total amount of data in the database and one giving some breakdown information on each of the experiments associated with each of the publications represented in the database. Each publication may be associated with a number of experiments investigating different hypotheses. You can link out to articles in PubMed by clicking on the PubMed hyperlinks. You may also load the dataset into the *Experiment Explorer* module by clicking on the little green flask icon ( ) next to the experiment name.

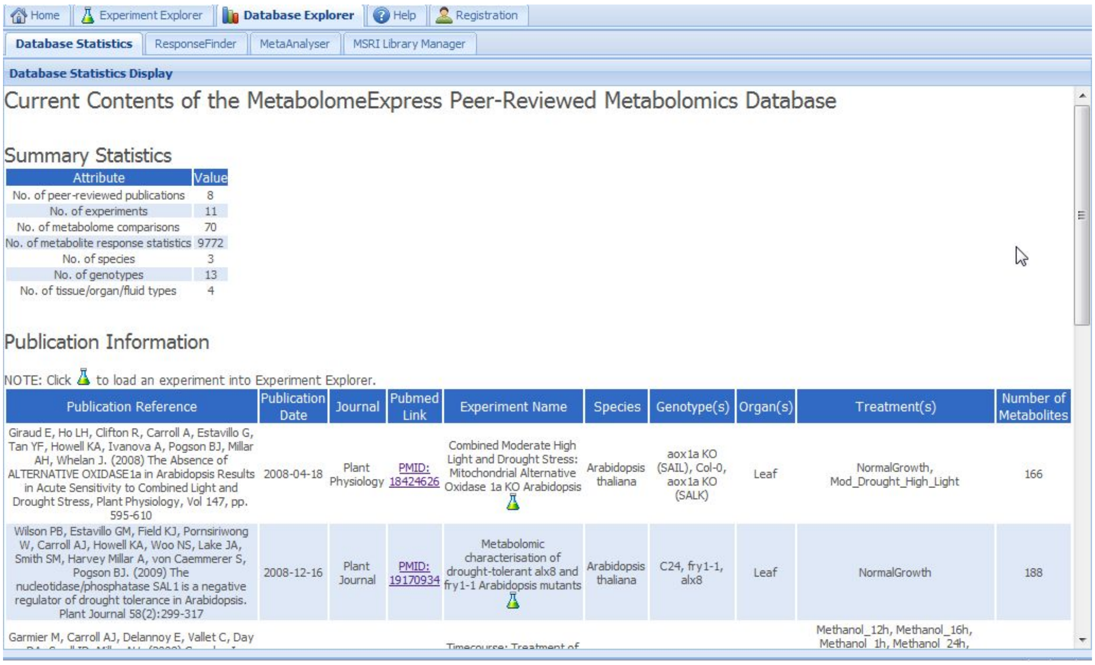

2.2 Finding experiments of interest using *ResponseFinder*

The *ResponseFinder* module allows you to search the MetabolomeExpress database for metabolite responses of interest based on metabolite name, minimum fold-change, maximum p-value, metabolite response directionality, species and organ. The screenshot below shows the *ResponseFinder* control panel set up to find any results where 2-Oxoglutarate was observed to be increased or decreased by at least 2-fold (and a p-value of 0.05 or less) in any organ of *Arabidopsis thaliana*.

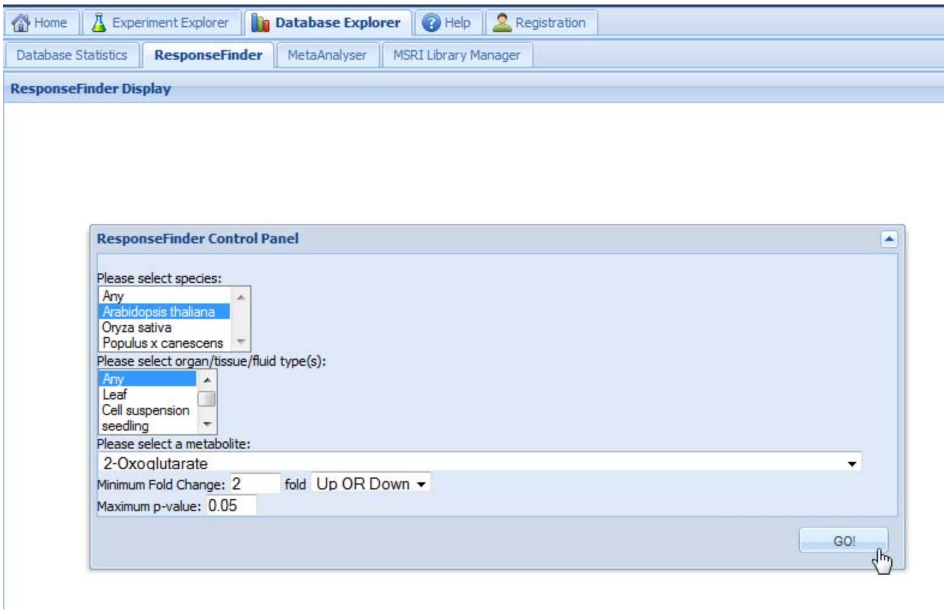

Clicking "GO!" retrieves the following results:

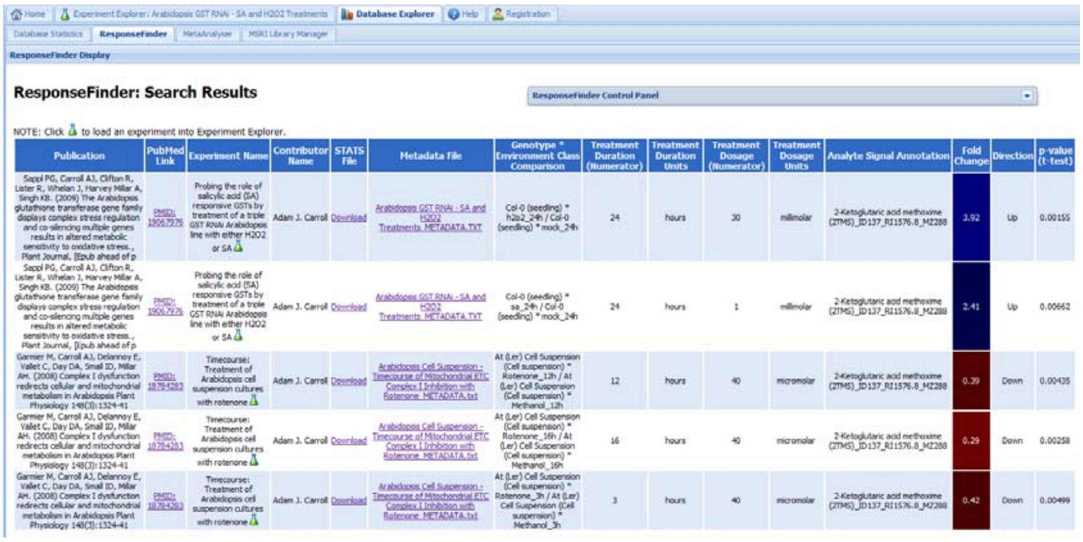

Publication	PubMed Link	Experiment Name	Contributor Name	STATS File	Metadata File	Genotype * Environment Class Comparison	Treatment Duration (Numerator)	Treatment Duration Units	Treatment Dosage (Numerator)	Treatment Dosage Units	Analyte Signal Annotation	Fold Change	Direction	p-value (t-test)
Sappl PG, Carroll AJ, Clifton R, Lister R, Whelan J, Harvey Millar A, Singh KB. (2009) The Arabidopsis glutathione transferase gene family displays complex stress regulation and co-silencing multiple genes results in altered metabolic sensitivity to oxidative stress., Plant Journal, [Epub ahead of p	PMID: 19067976	Probing the role of salicylic acid (SA) responsive GSTs by treatment of a triple GST RNAi Arabidopsis line with either H2O2 or SA	Adam J. Carroll	Download	Arabidopsis GST RNAi - SA and H2O2 Treatments METADATA.TXT	Col-0 (seedling) * h2o2_24h / Col-0 (seedling) * mock_24h	24	hours	30	millimolar	2-Ketoglutaric acid methoxime (2TMS)_ID137_RI1576.8_MZ288	3.92	Up	0.00155
Sappl PG, Carroll AJ, Clifton R, Lister R, Whelan J, Harvey Millar A, Singh KB. (2009) The Arabidopsis glutathione transferase gene family displays complex stress regulation and co-silencing multiple genes results in altered metabolic sensitivity to oxidative stress., Plant Journal, [Epub ahead of p	PMID: 19067976	Probing the role of salicylic acid (SA) responsive GSTs by treatment of a triple GST RNAi Arabidopsis line with either H2O2 or SA	Adam J. Carroll	Download	Arabidopsis GST RNAi - SA and H2O2 Treatments METADATA.TXT	Col-0 (seedling) * sa_24h / Col-0 (seedling) * mock_24h	24	hours	1	millimolar	2-Ketoglutaric acid methoxime (2TMS)_ID137_RI1576.8_MZ288	2.41	Up	0.00662
Garmier M, Carroll AJ, Delannoy E, Vallet C, Day DA, Small ID, Millar AH. (2008) Complex I dysfunction redirects cellular and mitochondrial metabolism in Arabidopsis Plant Physiology 148(3):1324-41	PMID: 18784283	Timecourse: Treatment of Arabidopsis cell suspension cultures with rotenone	Adam J. Carroll	Download	Arabidopsis Cell Suspension - Timecourse of Mitochondrial ETC Complex I Inhibition with Rotenone METADATA.txt	At (Ler) Cell Suspension (Cell suspension) * Rotenone_12h / At (Ler) Cell Suspension (Cell suspension) * Methanol_12h	12	hours	40	micromolar	2-Ketoglutaric acid methoxime (2TMS)_ID137_RI1576.8_MZ288	0.39	Down	0.00435
Garmier M, Carroll AJ, Delannoy E, Vallet C, Day DA, Small ID, Millar AH. (2008) Complex I dysfunction redirects cellular and mitochondrial metabolism in Arabidopsis Plant Physiology 148(3):1324-41	PMID: 18784283	Timecourse: Treatment of Arabidopsis cell suspension cultures with rotenone	Adam J. Carroll	Download	Arabidopsis Cell Suspension - Timecourse of Mitochondrial ETC Complex I Inhibition with Rotenone METADATA.txt	At (Ler) Cell Suspension (Cell suspension) * Rotenone_16h / At (Ler) Cell Suspension (Cell suspension) * Methanol_16h	16	hours	40	micromolar	2-Ketoglutaric acid methoxime (2TMS)_ID137_RI1576.8_MZ288	0.29	Down	0.00258
Garmier M, Carroll AJ, Delannoy E, Vallet C, Day DA, Small ID, Millar AH. (2008) Complex I dysfunction redirects cellular and mitochondrial metabolism in Arabidopsis Plant Physiology 148(3):1324-41	PMID: 18784283	Timecourse: Treatment of Arabidopsis cell suspension cultures with rotenone	Adam J. Carroll	Download	Arabidopsis Cell Suspension - Timecourse of Mitochondrial ETC Complex I Inhibition with Rotenone METADATA.txt	At (Ler) Cell Suspension (Cell suspension) * Rotenone_3h / At (Ler) Cell Suspension (Cell suspension) * Methanol_3h	3	hours	40	micromolar	2-Ketoglutaric acid methoxime (2TMS)_ID137_RI1576.8_MZ288	0.42	Down	0.00499

Most of the information here is self-explanatory but it is worth pointing out a few features of this table. Firstly, you can sort the table according to any column by clicking on its header. Secondly, you can load any of the retrieved experiments into the *Experiment Explorer* module by clicking on the little green flask icon ( ) next to the experiment name. Thirdly, double-clicking on any colour-coded fold-change value will load the underlying raw GC/MS signal regions into the *Raw Data Viewer* of the *Experiment Explorer* so that you can manually verify the automatic signal processing results. The screenshot below shows the result of double-clicking the top result in the result set shown above. The chromatographic overlay shows that the m/z 288 quantifier ion of 2-Ketoglutaric acid methoxime (2TMS) is clearly more intense in the 30 mM H_2O_2-treated samples compared to the mock-treated samples and the visually determined intensity ratio agrees quite well with the automatically-determined fold-change of 3.92 listed in the database.

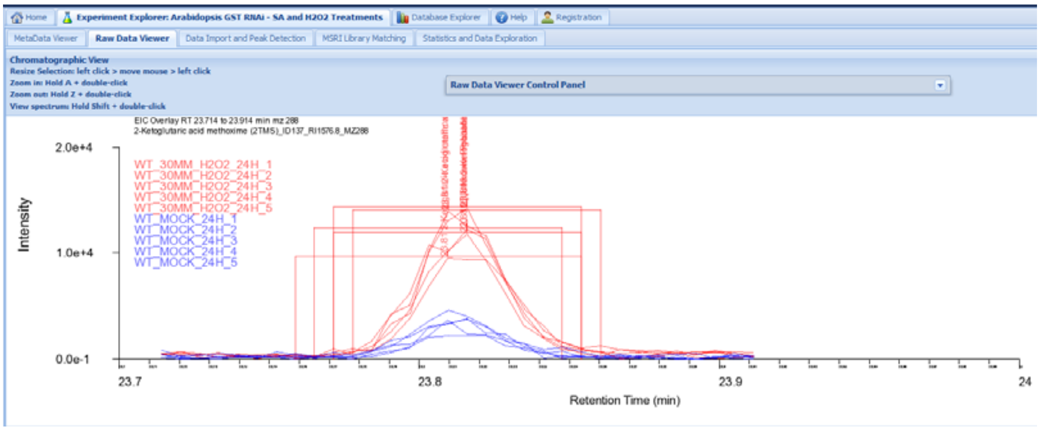

2.3 Comparing metabolite response patterns across multiple publications using *MetaAnalyser*

The *MetaAnalyser* module allows you compare metabolite response patterns across different experiments and different publications. *MetaAnalyser* assembles the metabolite response profiles from selected experiments, assembles them into a data matrix and carries out a 2-way hierarchical clustering before returning the organised

results in the form of an interactive DHTML heatmap and a PDF clustergram. *MetaAnalyser* also scores metabolites according to their 'responsiveness' (ie. how much variation they show across the selected dataset).

Using *MetaAnalyser* is very simple. You simply select which experimental class comparisons you wish to include in the analysis, specify whether to include metabolites of unknown structure and click “GO!”. The screenshot below shows the *MetaAnalyser* control panel set up to compare the metabolite response patterns of 4 experimental class comparisons (one for rice seedling anoxia at a 48 h timepoint and three for poplar root flooding at 5, 24 and 168 h timepoints.

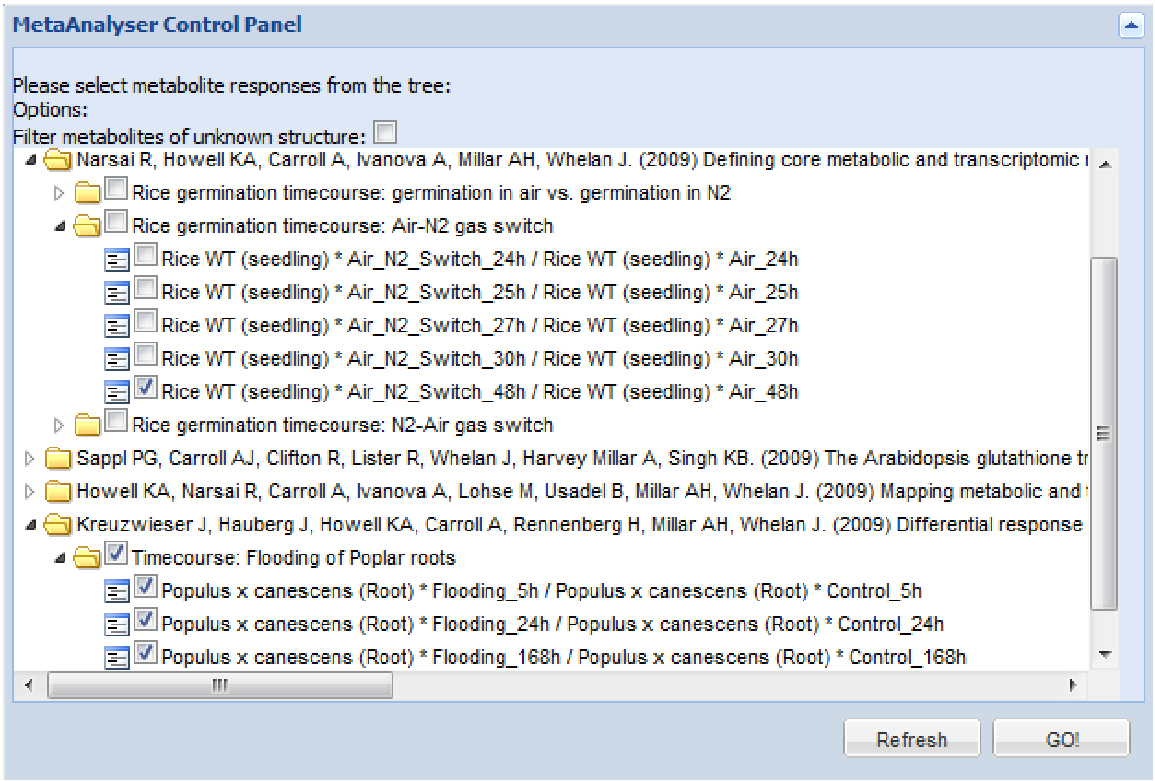

Clicking “GO!” generates the following result in the *MetaAnalyser* display (only the top few results are visible):

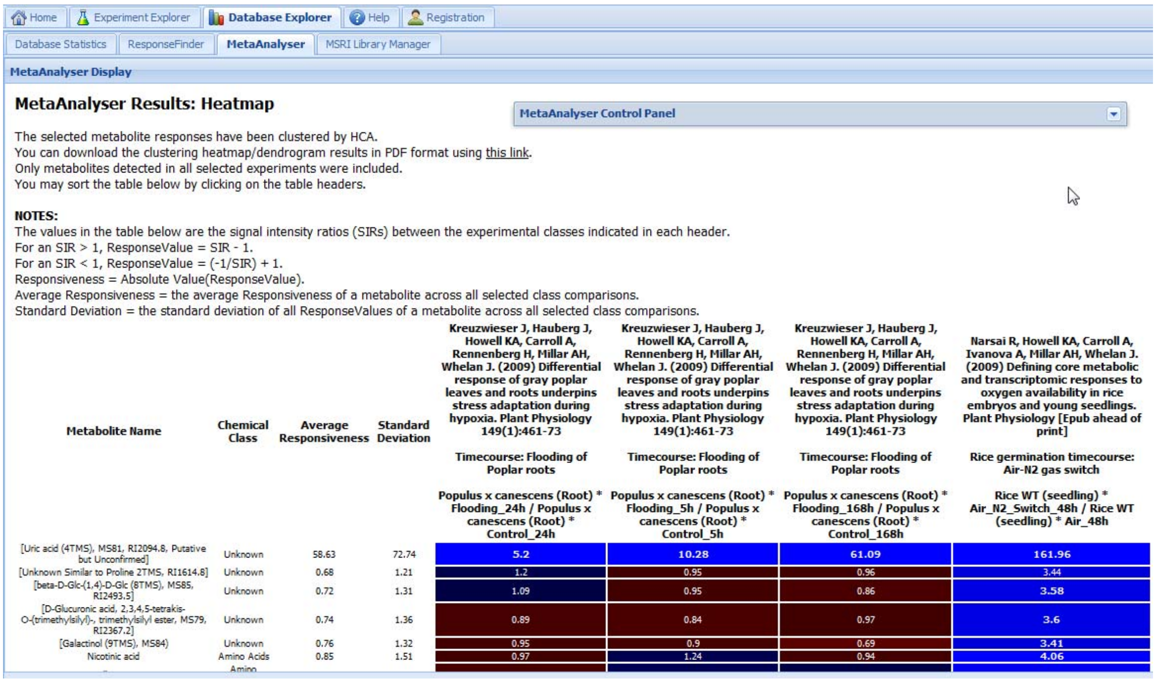

If you see an interesting result (like the 162-fold increase in the unknown metabolite with MS similarity to uric acid!), you can double-click the cell containing its signal intensity ratio and be taken to the raw GC/MS signals in the raw data viewer...

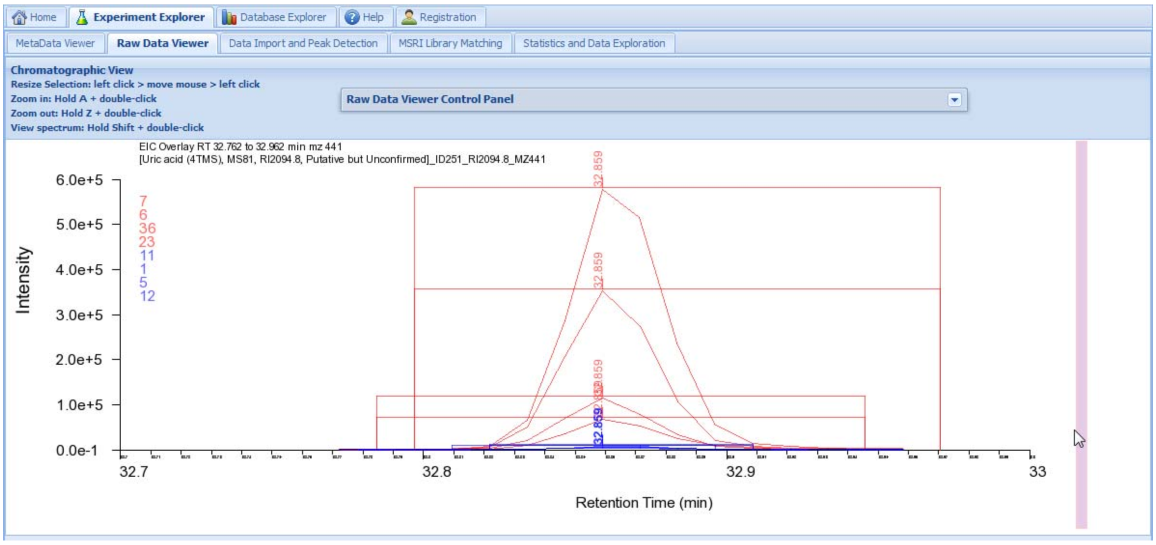

You can copy and paste the results into Excel for offline analysis if you like. You can also download a [2D HCA clustergram in PDF format](#) using the provided hyperlink. Here is a thumbnail image of the clustergram from this example analysis.

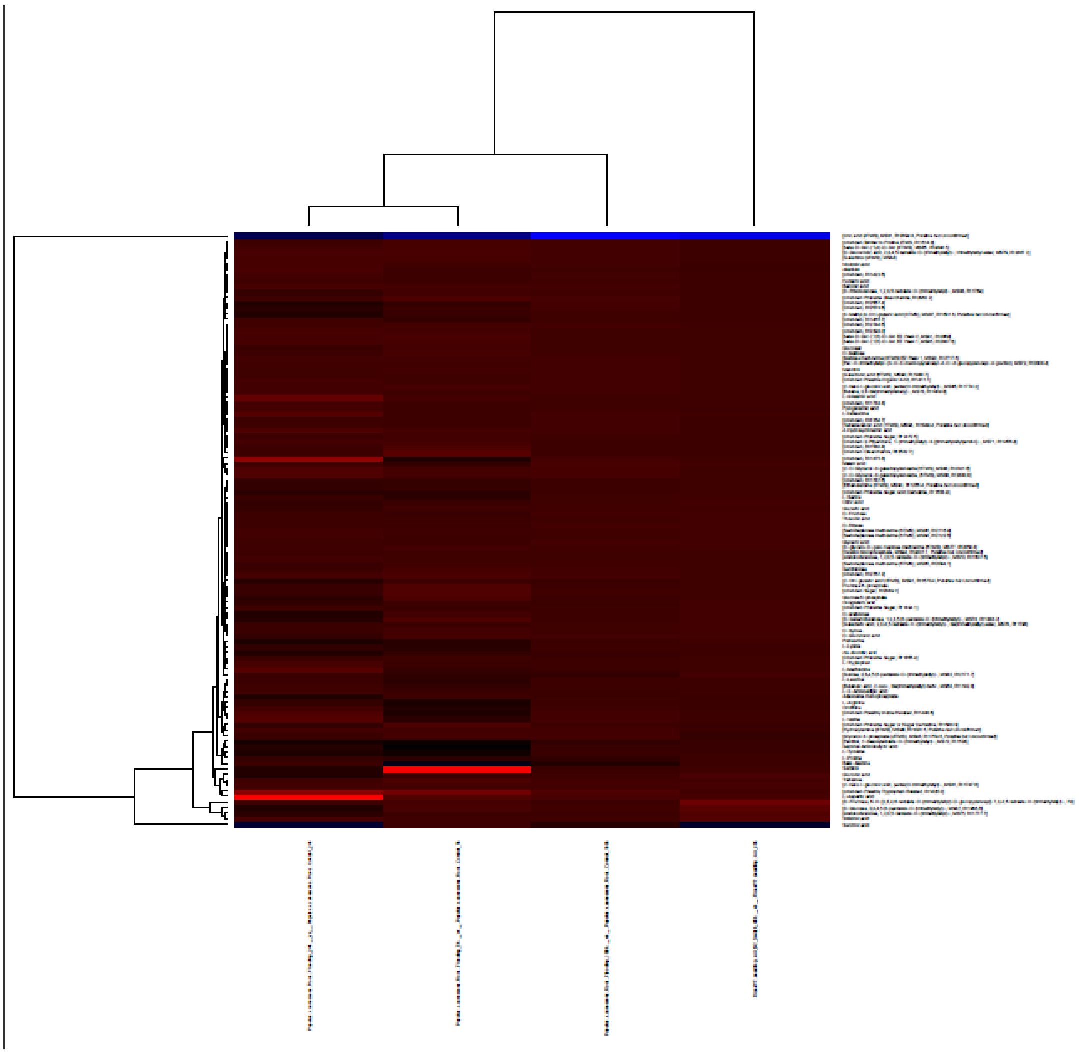

2.4 Identifying phenocopies using PhenoMeter (in development)

Most biologists are familiar with BLAST search algorithms which allow you to submit a DNA, RNA or protein sequence as bait and retrieve sets of homologous sequences, scored and ranked by similarity, from a large database. The PhenoMeter is an analogous tool that lets you use a metabolite response (ie. a set of metabolite fold changes and p-values for a particular class comparison) as bait and retrieve sets of other responses from the MetabolomeExpress database that are ranked (and scored) according to their similarity.

The interface for the PhenoMeter tool is currently exactly the same as the MetaAnalyser. Metabolite responses in the database are represented in a tree structure which begins at the publication level and branches down into the experiment level and then the class comparison level. You can make selections at any level using the check boxes provided. However, **you are advised only to select one or two class comparisons per search** in order to avoid excessively long query times.

The PhenoMeter control panel is shown below:

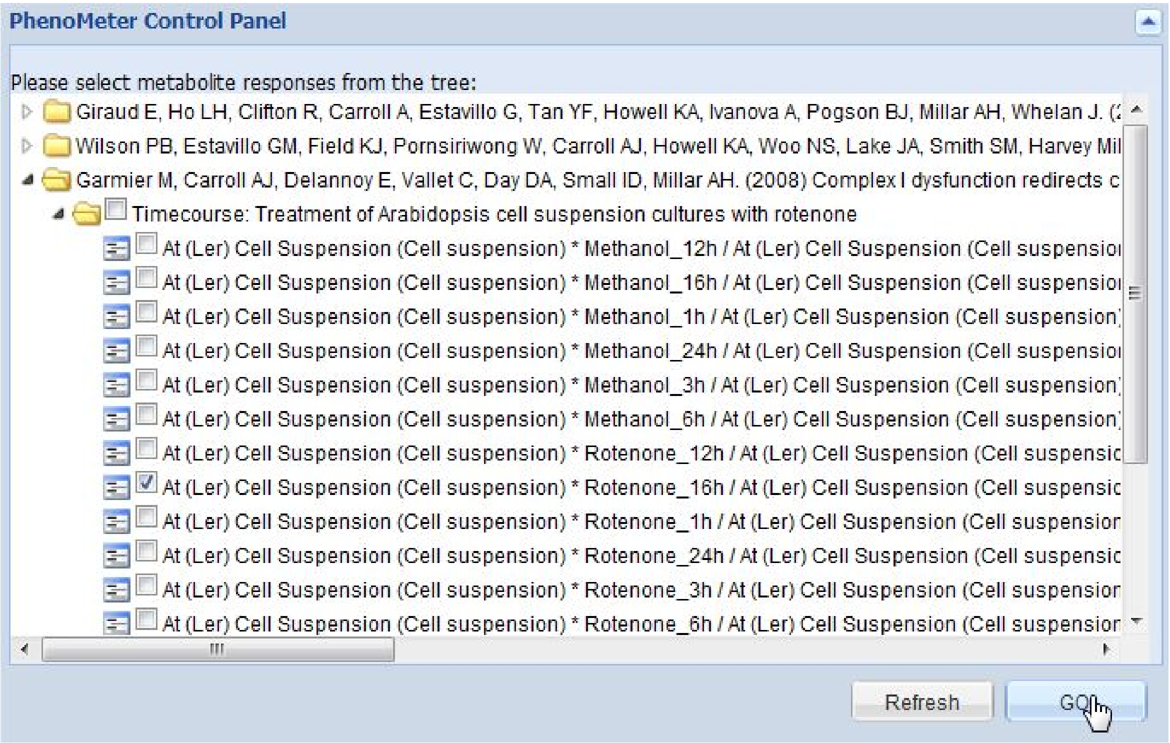

In the above screenshot, the response of suspension-cultured Arabidopsis cells to 16 hours of rotenone treatment (inhibition of mitochondrial respiratory chain complex I) has been selected as bait. Clicking the ‘GO!’ button yields the following results:

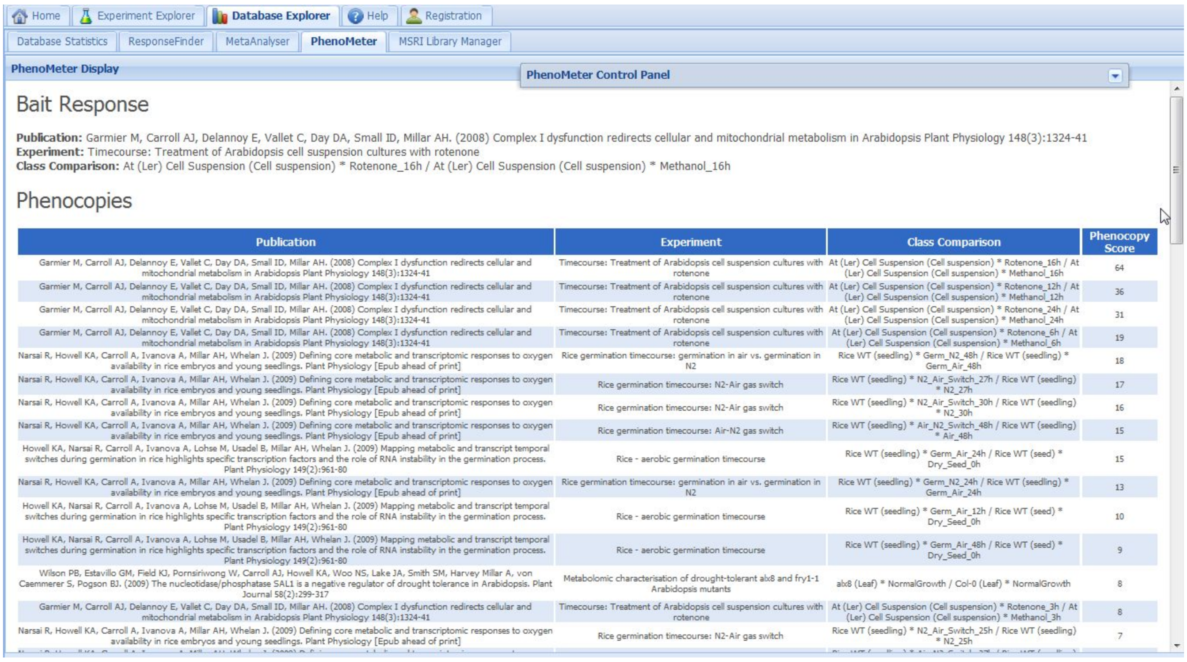

Retrieved metabolite responses receive 1 point towards their Phenocopy Score for each metabolite that responds significantly ($p<0.05$) in the same direction in both bait and tested response. For example, if alanine is significantly increased in the bait and it is also increased in a response in the database, then that response will receive 1 point. The more metabolites that respond significantly in the same direction as in the bait response, the more points a retrieved response will have. High Phenocopy Scores

may indicate that the bait and retrieved responses share a common underlying mechanism. This notion is supported by the fact that the top scoring hits here are the responses to rotenone at different time points and a response of rice seedlings to anaerobic germination compared to aerobic germination.

3 Processing and analysis of experimental GC/MS datasets with *Experiment Explorer*

3.1 Example dataset: Timecourse metabolomic analysis of plant cells treated with Antimycin A

The easiest way to learn is by example. So here we will provide a step-by-step guide that will show you how to start exploring the example GC/MS metabolomics dataset featured in the MetabolomeExpress publication: a 24-hour timecourse analysis of Arabidopsis thaliana plant cells responding to pharmacological inhibition of the mitochondrial electron transport chain with the classic respiratory inhibitor, Antimycin A.

A brief description of the example experiment:

At the beginning of the experiment, a number of 120 ml cell suspension cultures were sampled and immediately treated with either 25 µM Antimycin A (final concentration; supplied as a 100 µl dose suspended in methanol), methanol (100 µl) or water (100 µl). The cultures were then re-sampled after 1, 3, 6, 12, 16 and 24 hours of treatment. Hence, there are a total of 21 different treatments in the dataset (3 treatment groups, each including 7 different treatment durations). Given that each treatment was replicated 5 times, that works out to about 100 individual GC/MS runs or about 5-6 GB of raw GC/MS data.

Now that's enough background, let's load the data...

3.2 Loading a dataset

3.2.1 Loading a dataset from the Navigation panel

On the left side of the MetabolomeExpress interface, you should see a panel called 'Navigation' with a directory tree in it. This is where you browse through and load available datasets. First, expand the root node so you can see the experiment folders (indicated by little green flasks,) including the folder corresponding to the example dataset. Then, load the example dataset by left or right clicking on it to access the context menu and then left clicking on "Load Folder Contents".

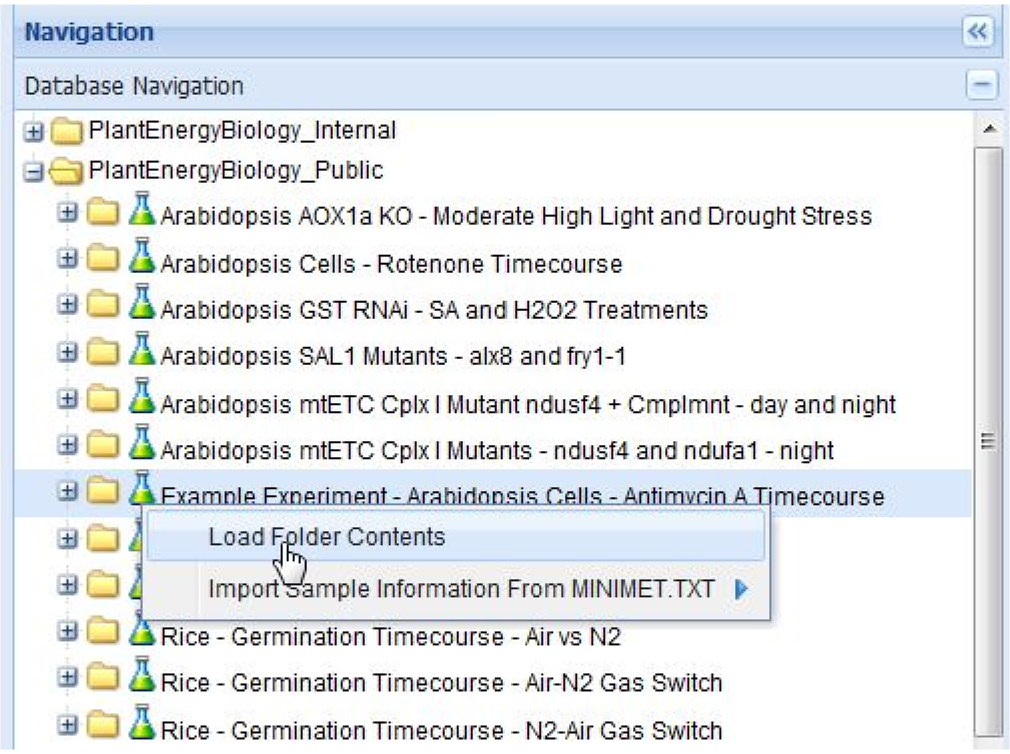

Wait a few moments while the information about the experiment is retrieved from the server and loaded into the interface. When everything is ready, the main tab panel should switch to the *MetaData Viewer* and all the standards-compliant metadata associated with the experiment should be visible, something like this:

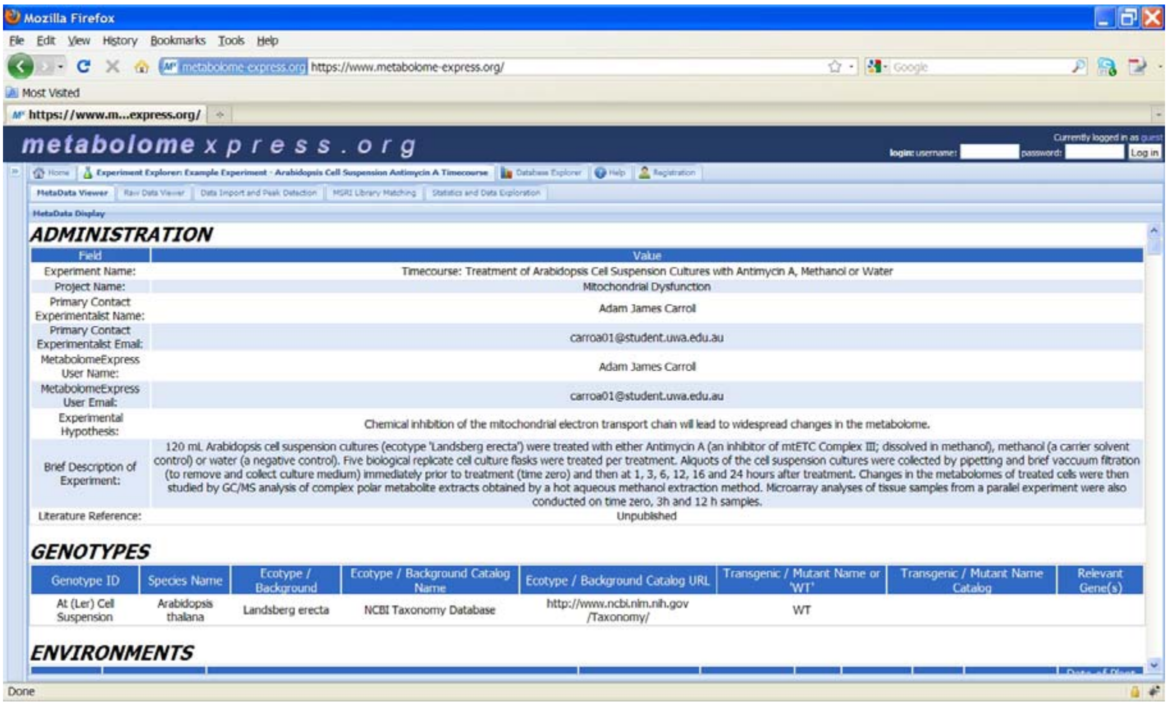

3.3 Using the Raw Data Viewer

Now, if you click on the Raw Data Viewer tab...

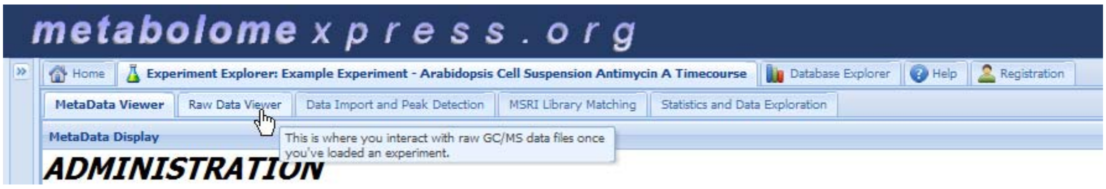

You will see a screen something like this:

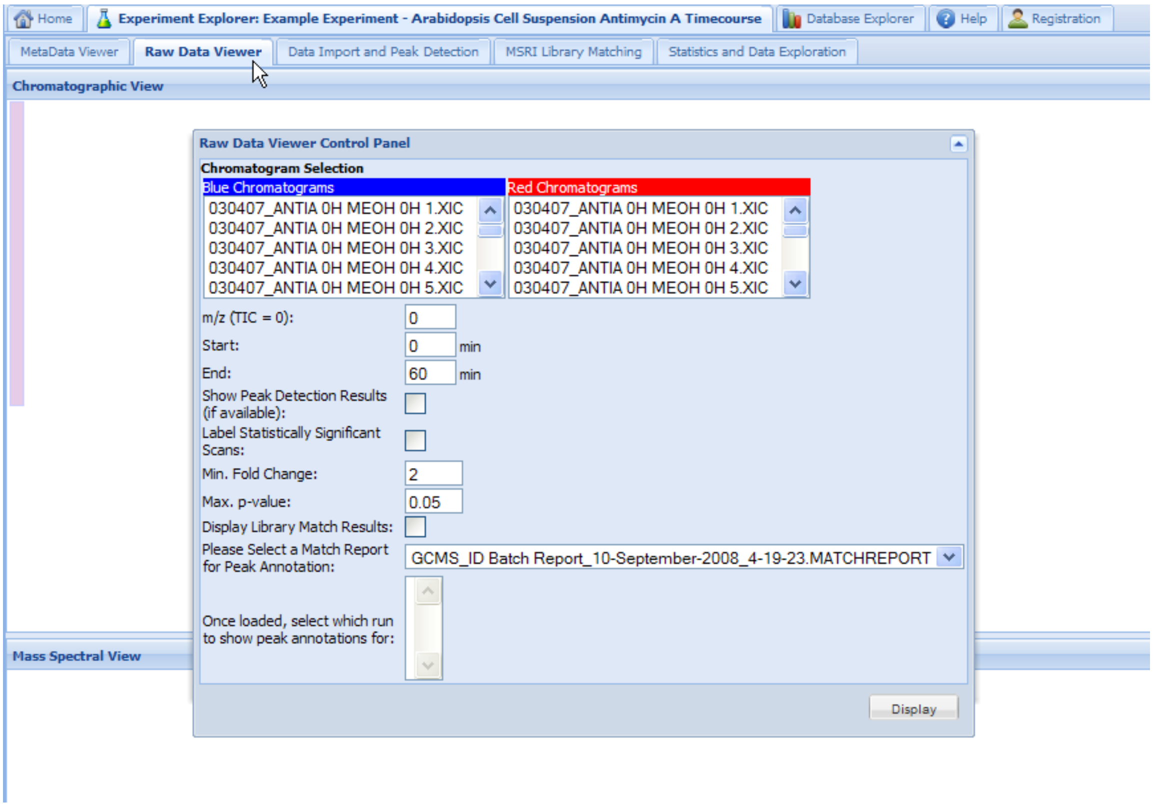

3.4 The raw data viewer control panel

To load some raw GC/MS data into the viewer, you must first select some file(s) using the *Raw Data Viewer Control Panel*. This is probably a good time to explain how the control panel works. Here's a close-up:

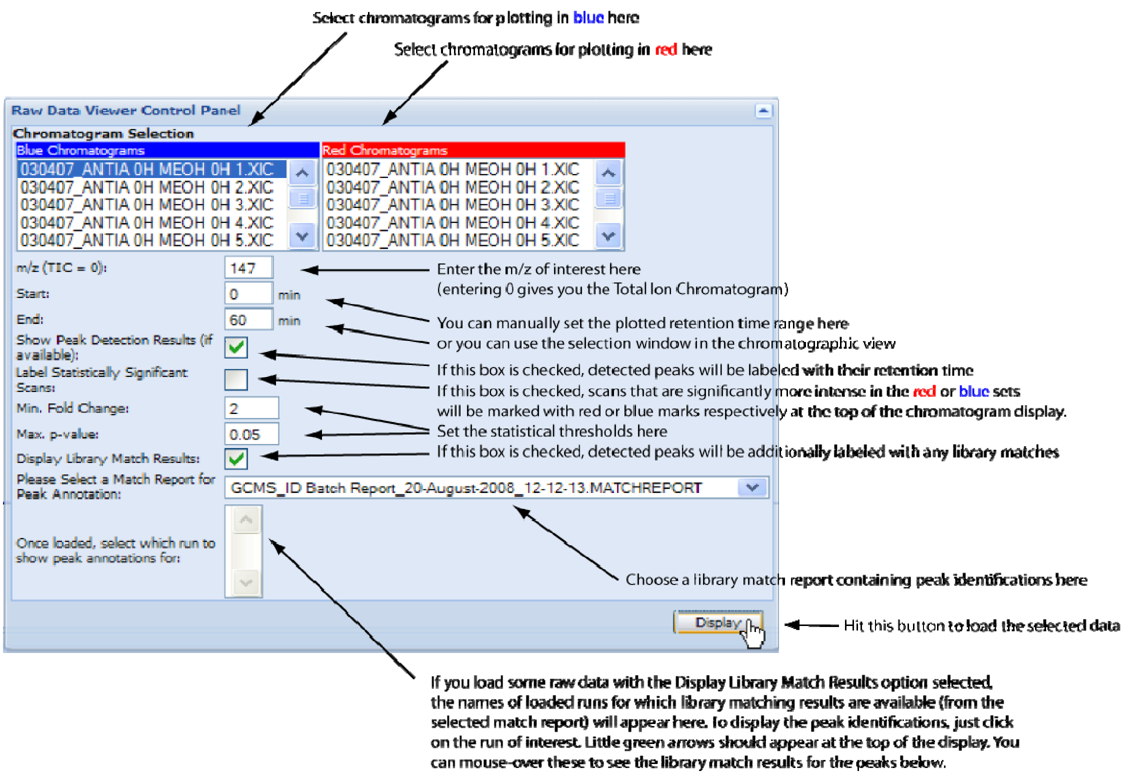

Now, set the *Raw Data Viewer Control Panel* up as shown in the image above by selecting the first chromatogram in the "Blue Chromatograms" selection box, setting the "m/z" to 147 and checking the "Show Peak Detection Results" and "Display Library Match Results" checkboxes. Then hit the "Display" button. Wait a moment, and the selected chromatogram should appear in the viewer like this (you may wish to collapse or drag the control panel out of the way at this stage):

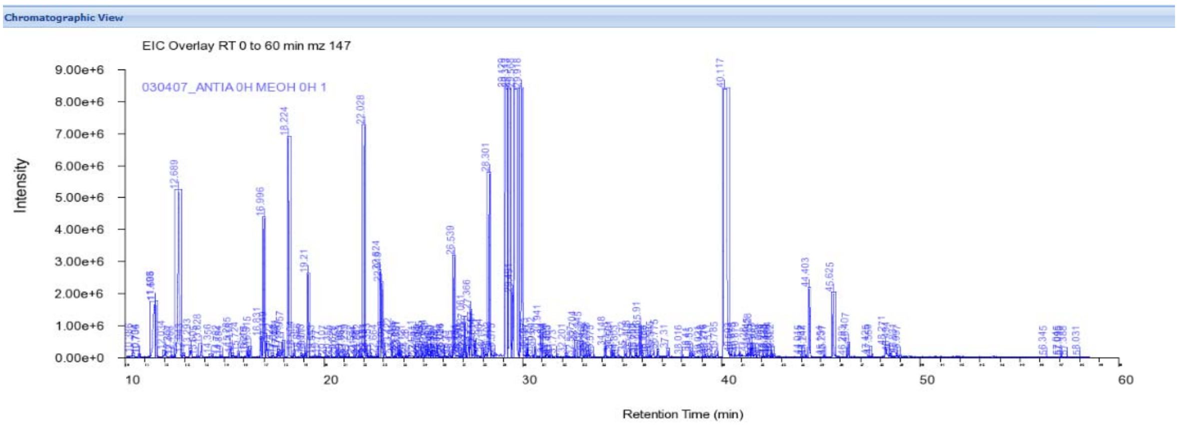

3.5 Zooming in and out

Now, in this case, there are a lot of peaks, making it hard to see what's going on. To zoom in, you must first select the region you wish to zoom in to by moving the pink selection window over it, move the selection window by moving the mouse cursor over the chromatogram. To resize the selection window, click once, resize to the desired width and then click again. Then, once you have the selection window covering the region of interest, hold down the 'A' key on your keyboard and double-click. Below is

the result of zooming in to the region of alpha-ketoglutarate methoxime (2TMS) which elutes at 23.86 minutes. Remember, to see the green peak annotation markers, you must select the run for display of peak annotations by clicking on its name in the bottom part of the control panel. Holding the mouse over green marker over the peak at 23.86 min shows that it was matched to alpha-ketoglutarate methoxime (2TMS). To zoom out again, hold the 'Z' key and double-click. The smaller your selection window, the further you will zoom out.

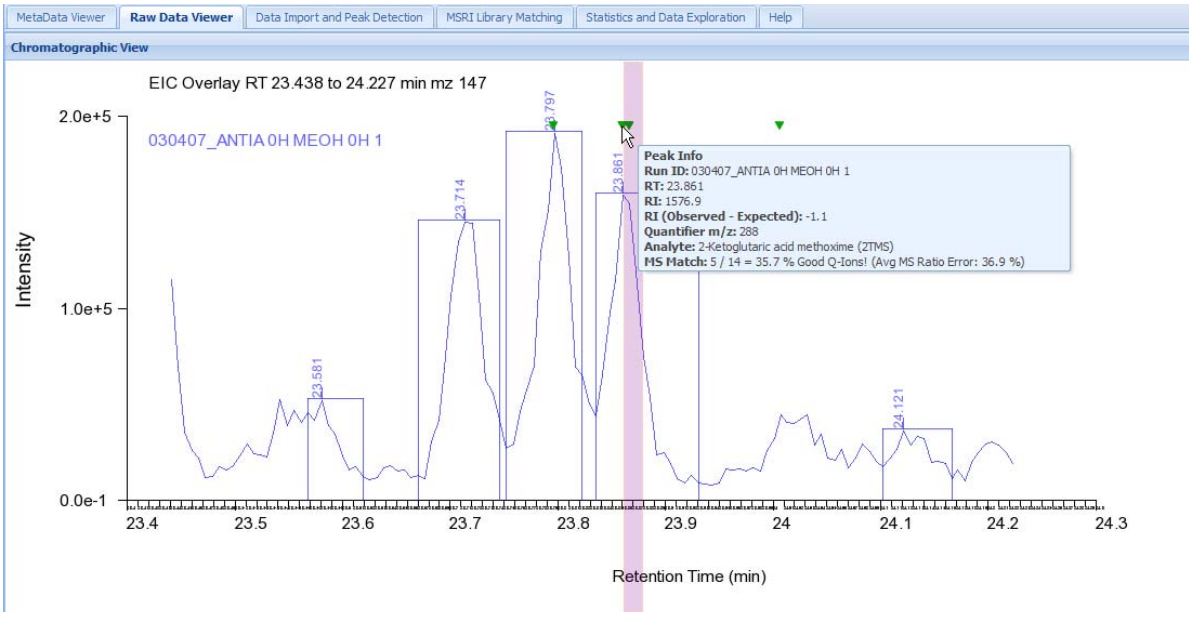

3.6 Viewing mass spectral scans

If you find an interesting peak, you can check out the mass spectral signal captured at that retention time by moving the left-hand edge of the selection window over that retention time, holding down the SHIFT key and double clicking. The mass spectral scan will then be displayed in the "Mass Spectral View" below the "Chromatographic View". Below is the spectrum observed at the apex retention time of the peak matched to alpha-ketoglutarate methoxime (2TMS).

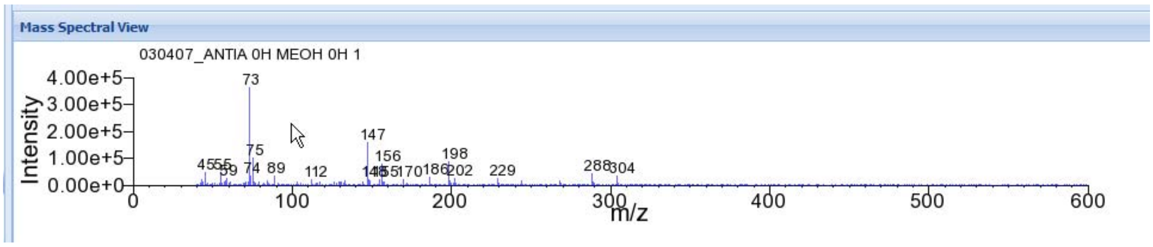

3.7 Finding differentially expressed peaks using the chromatographic statistical tool

To quickly find biologically interesting peaks, you can use the chromatographic statistical comparison tool. To see an example, set the *Raw Data Viewer Control Panel* up like this (it doesn't matter which runs are selected in the bottom part) and hit "Display":

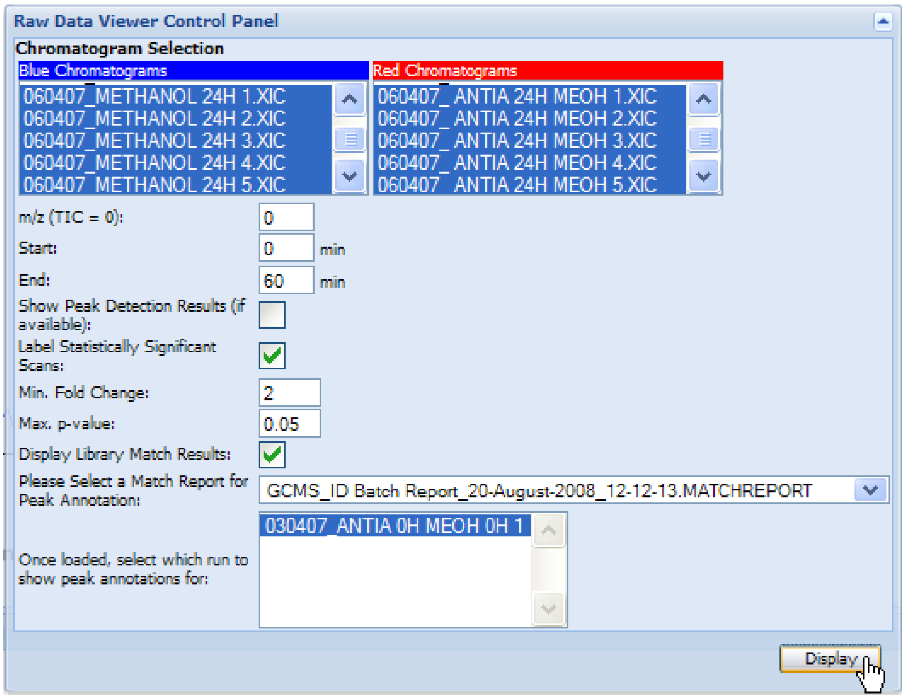

This is what the view should look like after clicking the 'Display' button and selecting one of the runs for display of peak annotations:

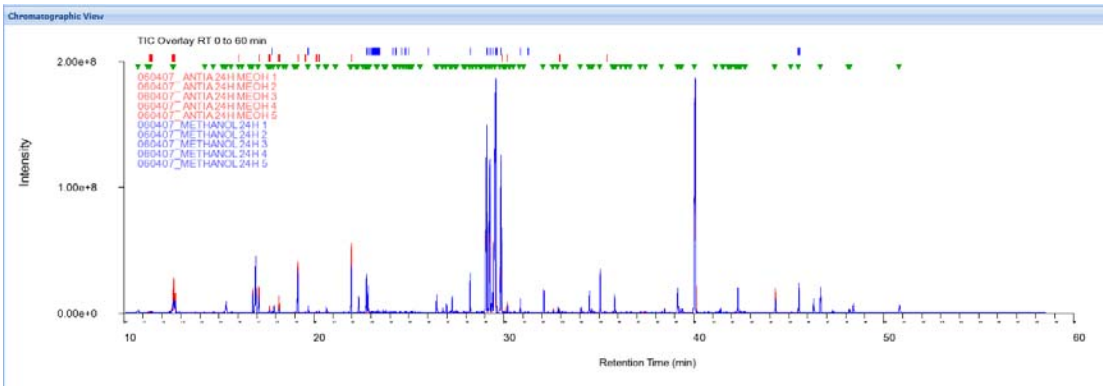

Notice the little red and blue markers at the top of the chromatogram. These indicate scans that are statistically significantly higher in the red or blue chromatograms respectively. If you zoom in on those peaks you will see that they are usually biologically responsive analytes. Hint: If you are only interested in really strongly responsive metabolites, you can increase the minimum fold change or decrease the maximum p-value settings in the control panel.

That's about all you need to know for the *Raw Data Viewer*. Now let's move on...

4 Data import and peak detection (registered users only)

You will notice that to the right of the *Raw Data Viewer* tab the next tab is called *Data Import and Peak Detection*. If you click on that tab you will see a screen like this:

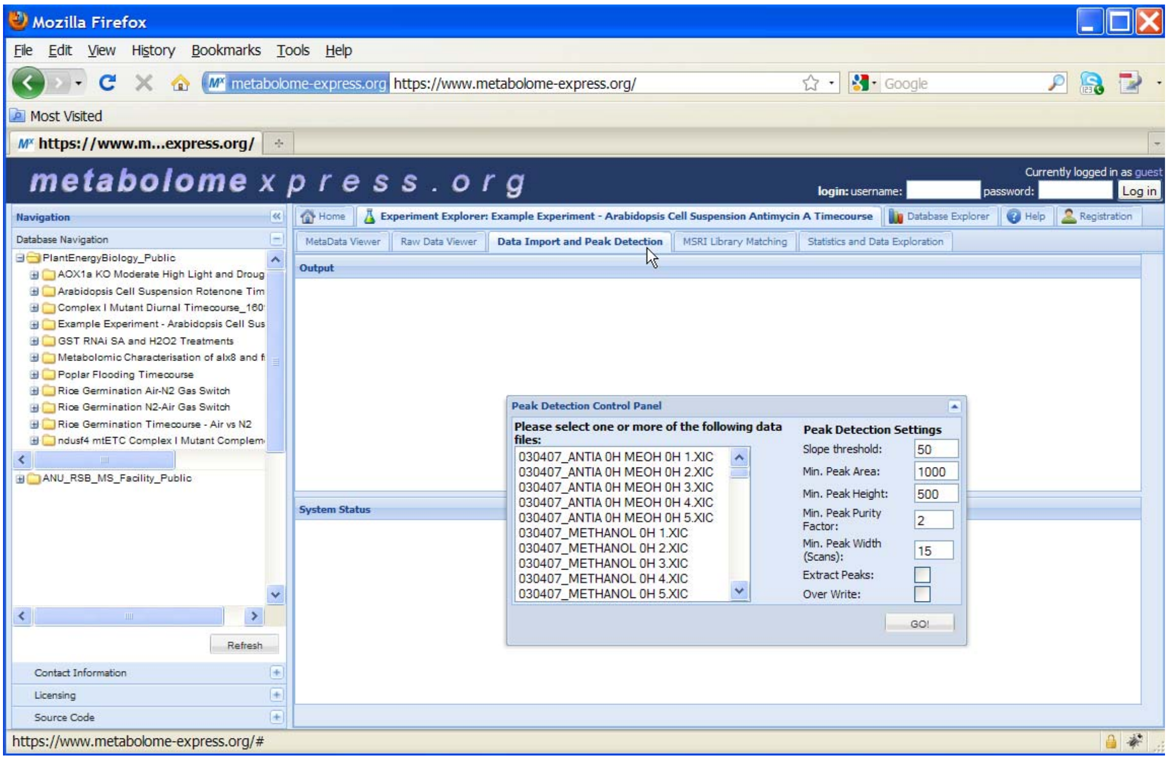

4.1 A Guide to the MetabolomeExpress PeakFinder Algorithm

The MetabolomeExpress *PeakFinder* algorithm is responsible for the detection and measurement of chromatographic peaks in extracted ion chromatograms (EICs). When a raw data file is sent for peak detection, the *PeakFinder* algorithm is passed each nominal mass (integer mass) EIC in the data file (as two equal-length vectors: signal intensity and retention time), one by one, until peaks have been detected in all EICs. The end result is a tab-delimited PEAKLIST table with columns for m/z, retention time, peak area, peak height, peak width, integration start time and end time, the scan numbers of the integration start and end points, the intensity of the signal at the integration start and end points and peak purity factor (defined as the proportion of the total integrated signal that lies above the lowest integration point).

The algorithm works in two phases. In the first phase, the algorithm moves from the start of the EIC to the end, recording sections of the signal that resemble chromatographic peaks. In the second phase, the algorithm checks each of the recorded sections to see if it meets the user-specified criteria for being a real chromatographic peak (min. peak area, min. peak width, min. peak height and min. peak purity factor). These user-specified parameters should be optimised whenever data from a new instrument type or brand is processed. Once peaks have been detected, you can review the peak detection results by visualising the raw data in the raw data viewer with 'Display Peak Detection Results' turned on.

The first phase begins by starting at the beginning of the EIC, taking a 3-point moving average of the signal intensity centered around the second scan point (ie. the average of the signal intensities at the first, second and third scan points), taking a three-point moving average of the signal intensity centered around the second scan point (the average of the signal intensities at the second, third and fourth scan points) and subtracting the first average from the second average. This value will be referred to as

the 'slope' of the signal, in this case between the second and third scan points. The algorithm then steps forward through the EIC, scan by scan, calculating the slope at each point until it encounters a slope value that exceeds the critical slope threshold specified by the user. When this happens, the algorithm is alerted to the fact that it could be running into rising section at the start of a chromatographic peak and starts recording retention time and intensity information (for later integration) until it encounters slope events that indicate that the end of the peak (or the start of a new peak) has been reached. If the algorithm is in the rise of a peak, it waits for the slope to become negative, indicating that the apex of the peak has been reached and the algorithm is now entering the falling part of the peak. As the algorithm moves down the falling part of the peak, it keeps recording the peak until the absolute slope value exceeds the critical threshold again. When this happens, the algorithm checks whether the slope is negative - which tells the algorithm that it is well past the top of the peak (where small, subcritical, but transient negative slopes might be encountered) and should start looking for the end of the peak, or positive - which tells the algorithm that it has encountered the rising part of a new peak that starts part of the way down the first peak. If the rising part of a second peak is detected in the down-slope of a current peak, the intersection point is given as the integration end time of the first peak and integration start time of the second peak. However, in most cases, there is no second peak rising out of the down-slope of the current peak and the algorithm continues recording the peak until the absolute value of the negative slope falls once again below the critical slope threshold. This event tells the algorithm that the end of the peak has been reached and it stops recording the peak, and keeps moving along waiting for the slope to rise above the critical slope threshold again. This process is continued all the way to the end of the EIC until all signals resembling peaks have been recorded.

In the second phase, each signal section recorded in the first phase is first examined in a number of ways:

- The retention times, scan numbers and intensities of the signals at the start and end points of the recording are recorded.

- The retention time (peak apex retention time) and intensity of the scan having the maximum signal intensity (the peak height) are recorded

- The sum of all the recorded intensities (the peak area) is calculated

- The proportion of total signal lying above the lowest integration point (the integration point with the lowest signal) is calculated (the peak purity factor)

The algorithm then compares the values calculated as described above with the user specified thresholds and if the peak meets all of the criteria, then it will be added to the PEAKLIST file along with all of its recorded characteristics. If it fails to meet any one of the thresholds, it is probably not a real peak and will be discarded.

4.2 The Peak Detection Control Panel

The figure below explains what all the different parts of the control panel are for:

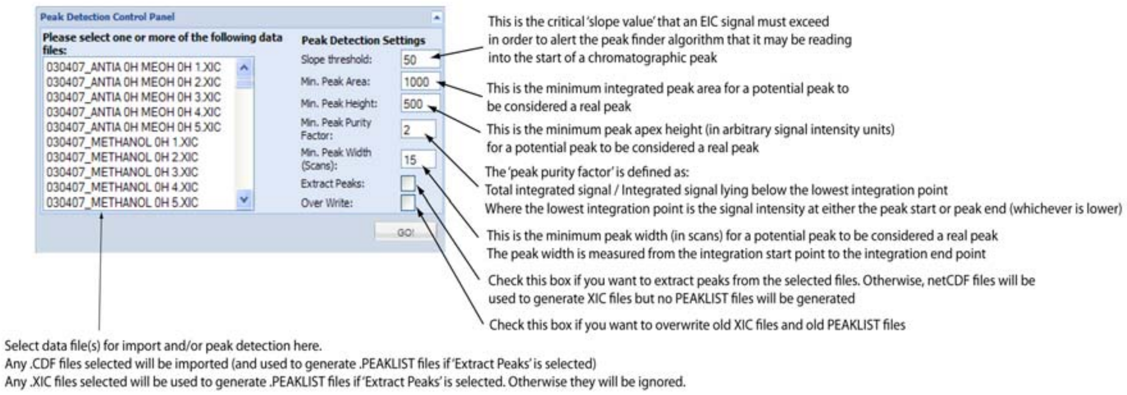

5 MSRI library matching

5.1 How to conduct an MSRI library matching process

MSRI library matching is defined as the identification of mass spectral signals corresponding to target analytes (in our case, metabolite derivatives) in a GC/MS data set by matching detected signals to entries in library of mass-spectral and retention index information for those target analytes (an MSRI library). If you click on the tab entitled *MSRI Library Matching*, you will be presented with a screen something like this:

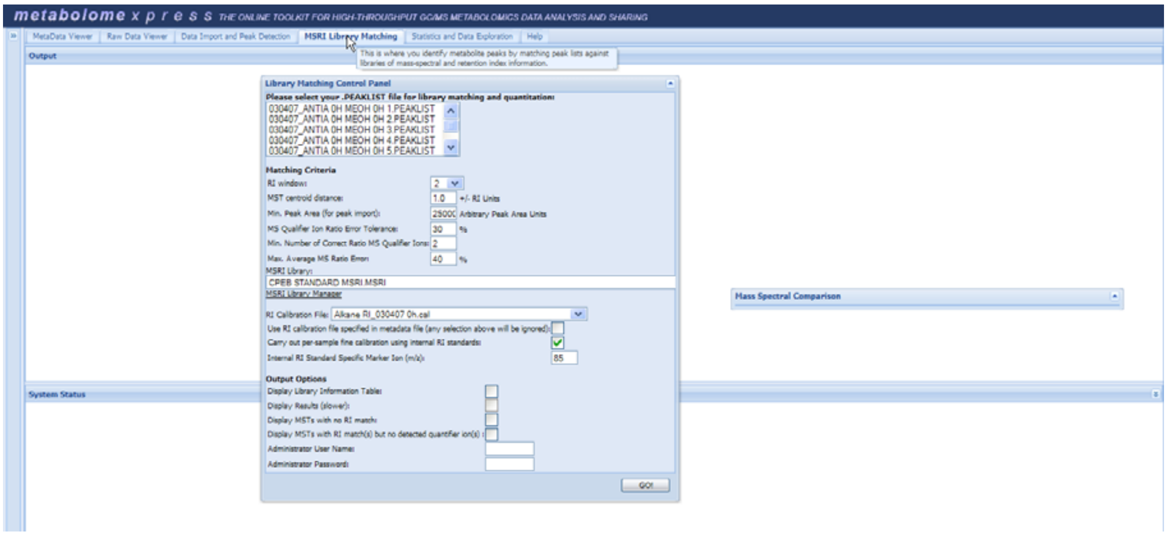

To initiate a library matching process, you use the control panel to select one or more .PEAKLIST files for library matching, set library matching criteria, choose an appropriate MSRI library and RI calibration file, specify whether to carry out fine per-sample RI calibration by the finding of internal RI standard peaks, specify an appropriately characteristic ion of the internal RI standards (m/z = 85 is good for commonly used n-alkanes), specify a number of output options and hit the 'GO!' button. You will need to be logged in and have write permissions on the relevant repository if you wish to process more than one sample and generate a .MATCHREPORT file in the experiment folder. If you aren't logged in or don't have write permission, you can still carry out library matching but only for a single file (the first selected file in the list), and your results won't be stored as a .MATCHREPORT

file in the experimental folder on the server - you will just be able to review the library matching results in the Output window.

The process used by the MSRI library matching algorithm is best described using the following decision tree.

Mass-Spectral Tag (MST) reconstruction and the MSRI library matching process

- Retention indices of all EIC peaks calculated relative to internal or external RI calibrant peaks (by linear interpolation)
- All EIC peaks assigned to 0.1 RI unit bins (Mass Spectral Tags)
- MSRI library matching procedure:

Step 1: Is Mass Spectral Tag (MST) within RI tolerance window of an MSRI entry?
IF YES: Go to Step 2
IF NO: Move on to next MST and begin at Step 1

Step 2: Does the MST contain any of the MSRI library-specified quantifier ions?
IF YES: Gather the m/z:intensity pairs from all MSTs within
the user-specified MST centroid distance (+/- 1.0 RI Units by default)
and merge these temporarily with the current MST.
Count the number of other ions in the merged MST that are within
a set percentage of the expected intensity based on the
intensity of the quantifier ion and the full mass spectrum
in the MSRI library. Calculate the average % deviation of all
ions from their expected intensities. Move on to Step 3.
Repeat for each detected quantifier ion.
IF NO: Repeat Step 2 for any remaining RI matches in the MSRI
library. If no more RI matches remain, move on to next MST
and begin at Step 1.

Step 3: Based on results of Step 2, does the temporarily merged MST contain at least the minimum
number of expected-ratio qualifier ions AND have an average ion intensity
deviation below the specified threshold?
IF YES: Add match details to tab-delimited .MATCHREPORT file
using the integrated peak area of the quantifier ion as
the reported signal intensity for the matched analyte.
IF NO: Discard MST and continue.

5.2 Interacting with MSRI library matching results

When an authorised user submits one or more .PEAKLIST files for library matching, each .PEAKLIST file gets searched for peaks matching library entries and positive matches across the entire set of .PEAKLISTs are reported in a tab-delimited .MATCHREPORT file which appears in the experimental folder (remember to reload the experiment or hit the 'Refresh' button on the Statistics and Data Exploration control panel in order to see the report in the relevant control panels). If the 'Display Results' option is selected, you will be presented with a screen like this (for this example we

have selected the .PEAKLIST file called '030407_ANTIA_0H_MEOH_0H_1.PEAKLIST' which corresponds to the first biological replicate cell culture flask sampled just prior to being treated with Antimycin A (the time-zero time-point). To try the example set the library matching control panel up as shown below and hit the 'GO!' button:

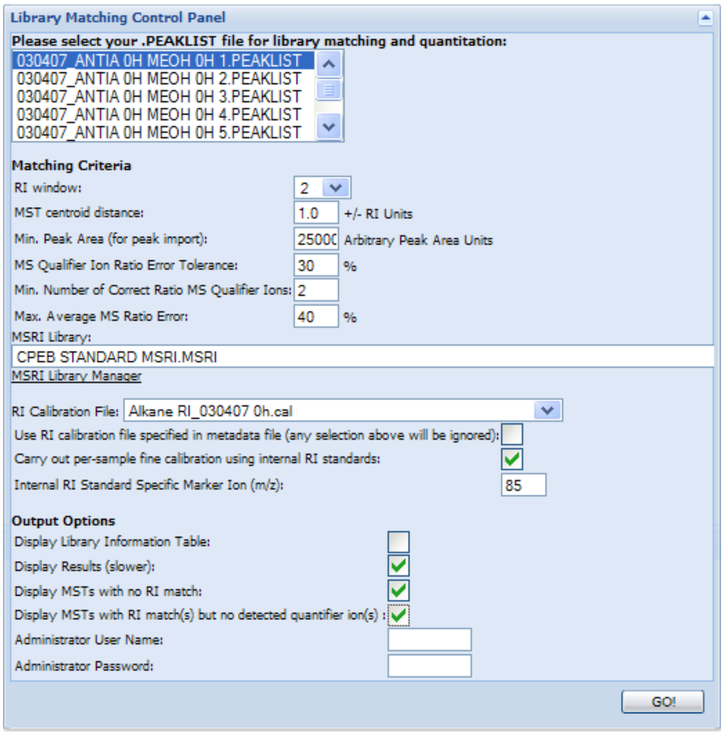

A progress bar should appear in the lower part of the screen. Wait until progress is complete and in a few moments, you should be presented with a large interactive report table in the output window, like shown below (you may wish to collapse the control panel at this point, to get it out of the way):

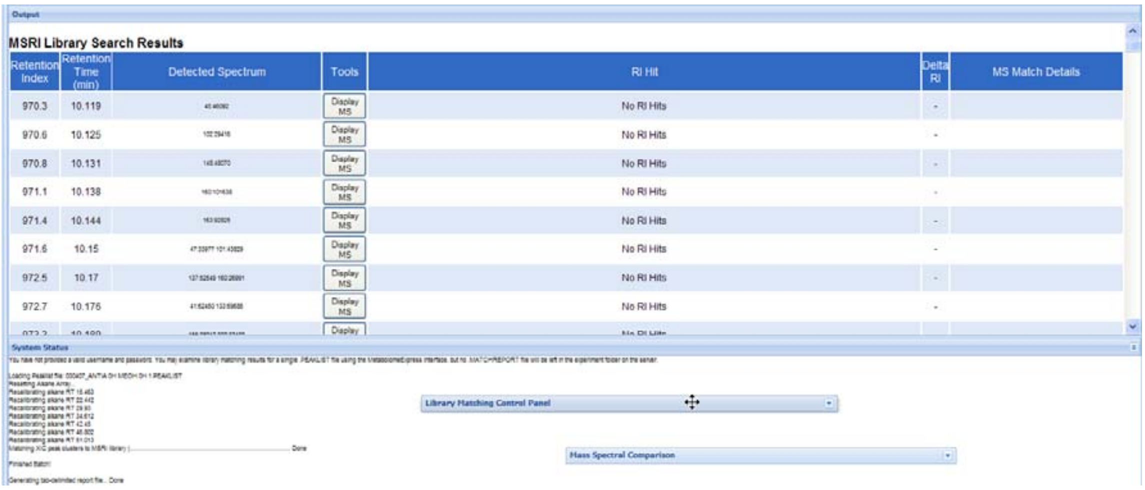

You can probably tell by looking at the table that each row corresponds to another 0.1 RI unit bin of EIC peaks (an MST, by our definition). None of the MSTs you can see in the screenshot above had an RI match in the searched RI library - they are all weak-intensity MSTs made up of a few tiny EIC peaks. However, if you scroll down you will

begin to see MSTs with something in the 'RI Hits' column. If the name of the RI Hit is black, it indicates that the MST RI falls within the RI Tolerance Window (default = +/- 2 RI Units) of the corresponding library entry, but the identification wasn't supported by either the presence of a library specified quantifier ion or by similarity between the ion ratios of the MST and ion ratios of the library spectrum. If the name of the RI hit in the RI Hits column is blue (rather than black), it has been positively matched to a library entry because one or more of the library-specified quantifier ions was found (indicated in red text) and the MST ion ratios agreed with those in the library spectrum, within the user-specified tolerance parameters.

Below is an example of a nice, clear positive match to Glycine (2TMS):

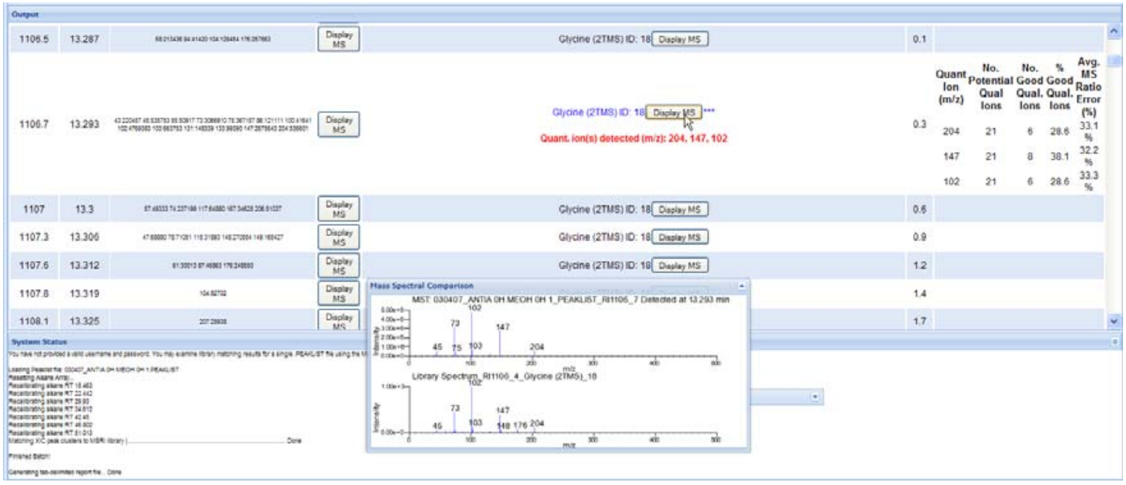

You can see the MST containing the library-specified quantifier ions (labelled with "**Quant. ion(s) detected (m/z): 204, 147, 102**"). If you click the 'Display MS' buttons next to the list of the MSTs mass:intensity data and the name of the RI Hit, you can display the MST spectrum and library spectrum, respectively, in the 'Mass Spectral Comparison' window as shown in the screenshot above.

6 Statistics and data exploration

Click on the tab entitled 'Statistics and Data Exploration' and we can go through the various statistical tools. Remember, to use these tools, you must have already loaded a dataset as described earlier.

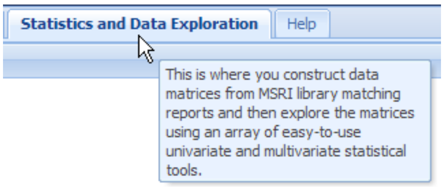

You will then see a screen like this:

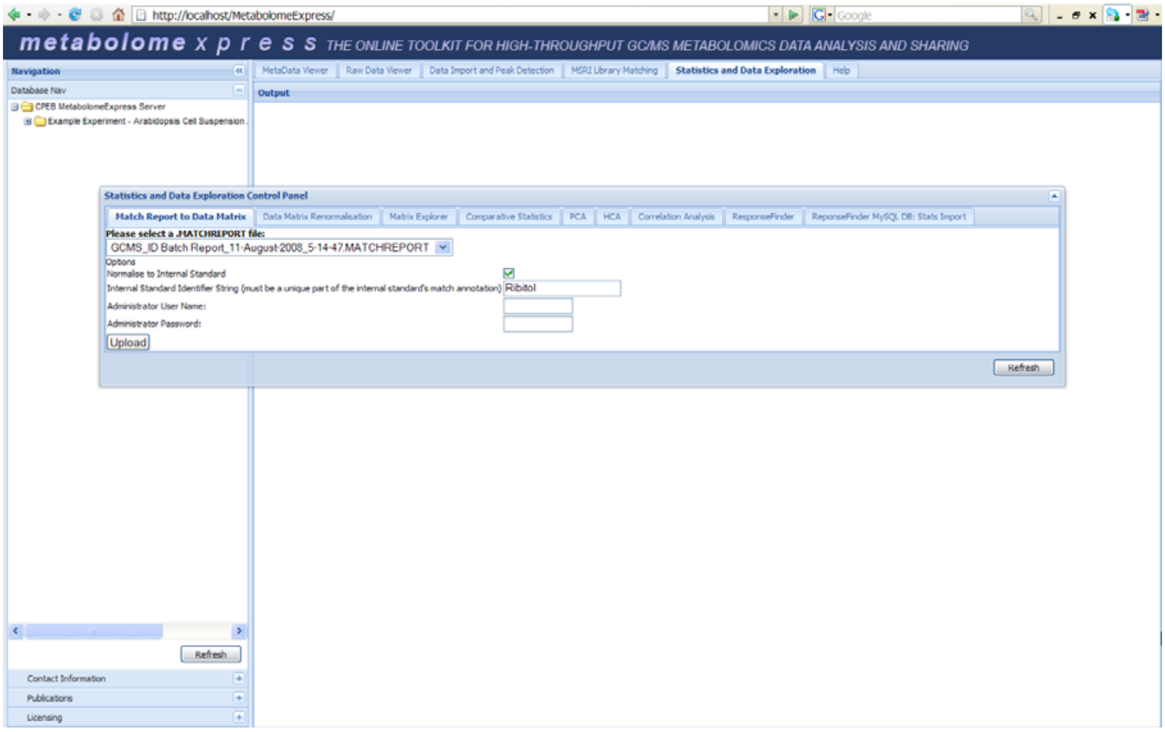

The "Statistics and Data Exploration Control Panel" is where all statistical analysis procedures are initiated. The results are displayed in the "Output" window.

6.1 Construction of data matrices from MSRI library-matching reports

Once MSRI library matching has been carried out, the next step is to assemble a data matrix from the MSRI library matching report. This process arranges all the results in the match report into a table where instrument runs are represented as columns and the various detected signals are represented as rows.

You will require write permission on the repository containing the dataset to do this. If you are analysing someone else's public dataset for which you don't have write permission, they will most likely have already created a data matrix for you.

6.1.1 Some notes on normalisation and quality control

To control for pipetting errors and variations in starting sample mass/volume, data matrix construction usually involves normalisation to some internal standard peak area and also to tissue mass/volume. This is achieved by dividing the peak area values in each column (ie. each sample) by the internal standard peak area measured in that sample and then dividing that result by the mass or volume of biological sample that was extracted to produce the sample. The MetabolomeExpress *Match Report to Data Matrix* tool currently provides two different internal standard normalisation options. One approach is to 'normalise to a single internal standard' that is added to each extract at some known, constant concentration. You can specify which signal in the data represents the internal standard by entering a unique identifier string that is present in the name of internal standard signal in your library matching results. For example, there is only one library entry called 'Ribitol' in the CPEB STANDARD

MSRI.MSRI library used to process the example dataset, so using the identifier string 'Ribitol' will only match the 'Ribitol' internal standard peak.

Normally, pipetting errors are relatively small and the internal standard peak area in each sample should be pretty much the same. A large deviation of an internal standard peak area from the median internal standard peak area is therefore a good indication that something more sinister than small pipetting errors has gone wrong and the data should therefore not be trusted. Similarly, if the internal standard peak is unusually small or cannot be found at all, alarm bells should ring! The MetabolomeExpress *Match Report to Data Matrix* tool includes options

6.1.2 Raw data-assisted missing value replacement

When a data matrix is constructed, there are almost invariably cases where a particular metabolite has been detected in some samples but not in others. This gives rise to 'missing values'. As many popular multivariate analysis techniques such as PCA and HCA cannot deal with missing values, it is necessary to fill these with some kind of proxy value. Many tools that carry out data matrix construction will either leave the missing values blank or will try to mathematically impute the real value. Alternatively, some will set all the missing values in a matrix to some low number based on the assumption that the value was missing because the compound's signal was below the baseline. Sometimes this is a valid assumption but with most mass-spectral library matching-based approaches, it is not.

Quite often, missing values arise because perfectly valid and clear signals did not, for some reason, quite get past a stringent library matching filter. Therefore, an increasingly popular alternative to all the previously mentioned approaches is to use chromatographic information from cases where the library matching led to positive matches to locate the missed signals in the raw data for which library matching failed. This is the approach used by the MetabolomeExpress *Match Report to Data Matrix* tool. When this tool encounters a missing value, it determines from the signal annotation which m/z channel was used as the quantifier ion in that row of the matrix and uses the integration start times and end times from positive matches in the library match report to determine the average peak start and peak end retention times of the signal (using only retention times from runs acquired in the same instrument batch sequence according to the Batch Sequence ID values for the 'Analytical Runs' in the _METADATA.TXT file). It then reads the raw data file showing the missing value and integrates the appropriate m/z signal from the average start time to the average end time and places this value in place of the missing value. Values obtained in this way are flagged with an asterisk (*) in the resulting tab-delimited .mzrtMATRIX file. You can always check the peaks later with the raw data viewer to convince yourself that the numbers make sense based on manual interpretation of the raw data.

IMPORTANT NOTE: When an .mzrtMATRIX file is submitted for comparative statistical analysis, class comparisons for which at least one class had more than 50% missing values will be excluded from the result set and replaced with an 'X'.

6.1.3 How to build a data matrix using the *Match Report to Data Matrix* tool

Matrix construction is initiated using the "Match Report to Data Matrix" tab.

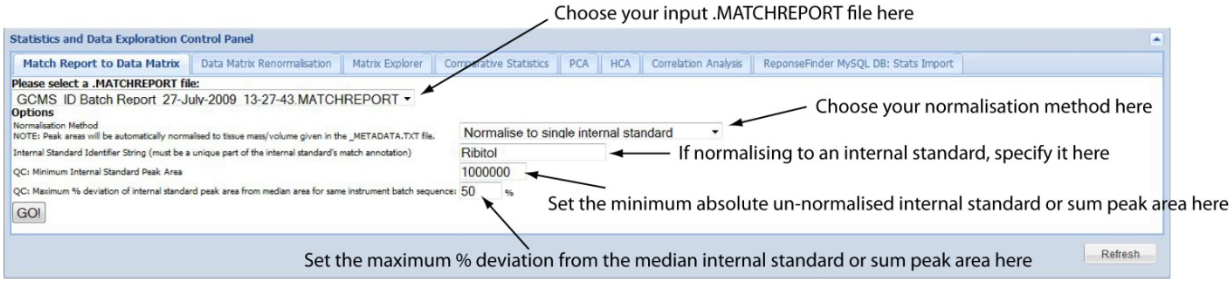

To build a matrix, select your .MATCHREPORT file; set your normalisation and QC parameters and click the ‘GO!’ button. You will then be given an opportunity to select which runs you would like to include in the matrix:

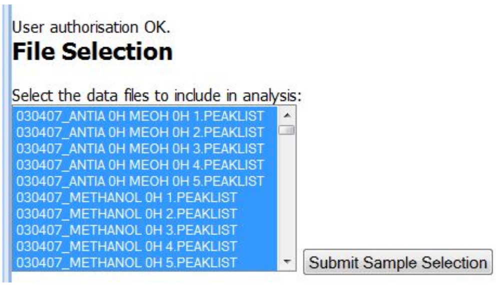

Make your selection and click the ‘Submit Sample Selection’ button.

A progress bar will then appear in the Output window while the matrix is assembled. This process can take a little while if there are lots of files.

NOTE: If you want your peak areas to be normalised to sample mass/volume and properly annotated with sample class information, you will need to provide this information by having a functional *_METADATA.TXT file in the experiment folder. The easiest way to create one of these is to import a basic set of sample information from a MINIMET.TXT file (see Step 4 in Section 1.4 - Uploading and managing your datasets via FTP). If no metadata is provided, samples will be assumed to have equal mass/volumes and class attributes in the matrix column headers will be set to “Unknown” (as in the screenshot below):

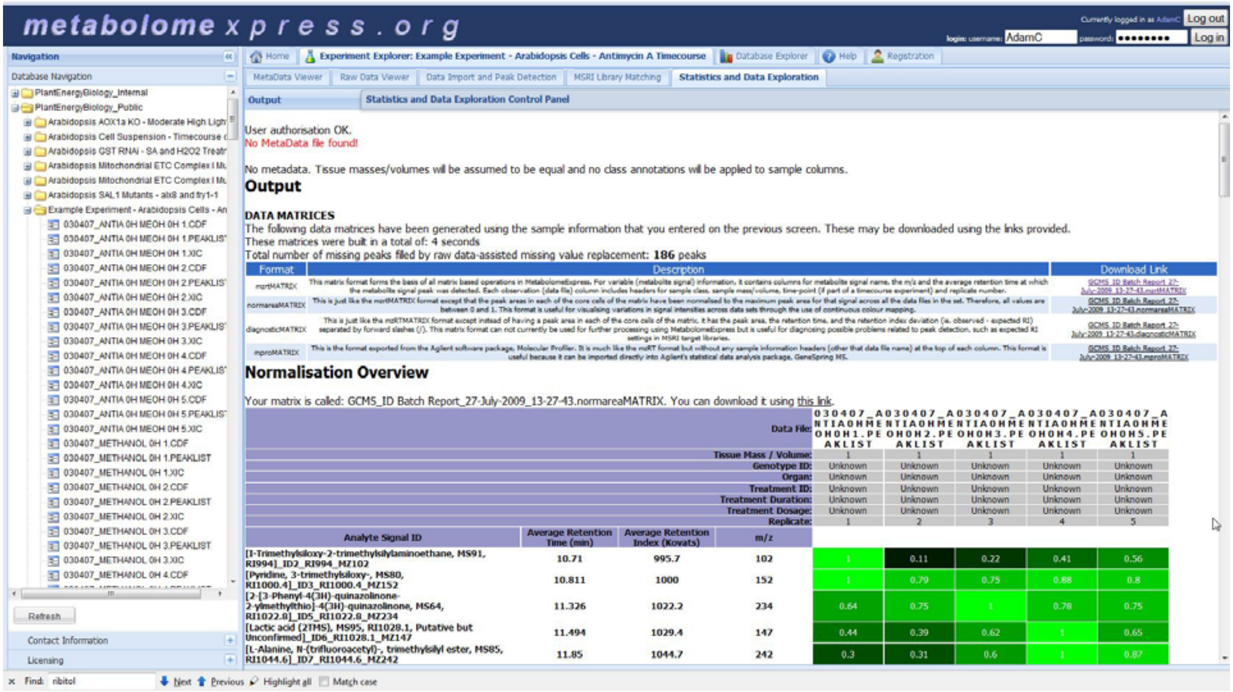

If you have a valid metadata file in the experiment folder, the result will look more like this:

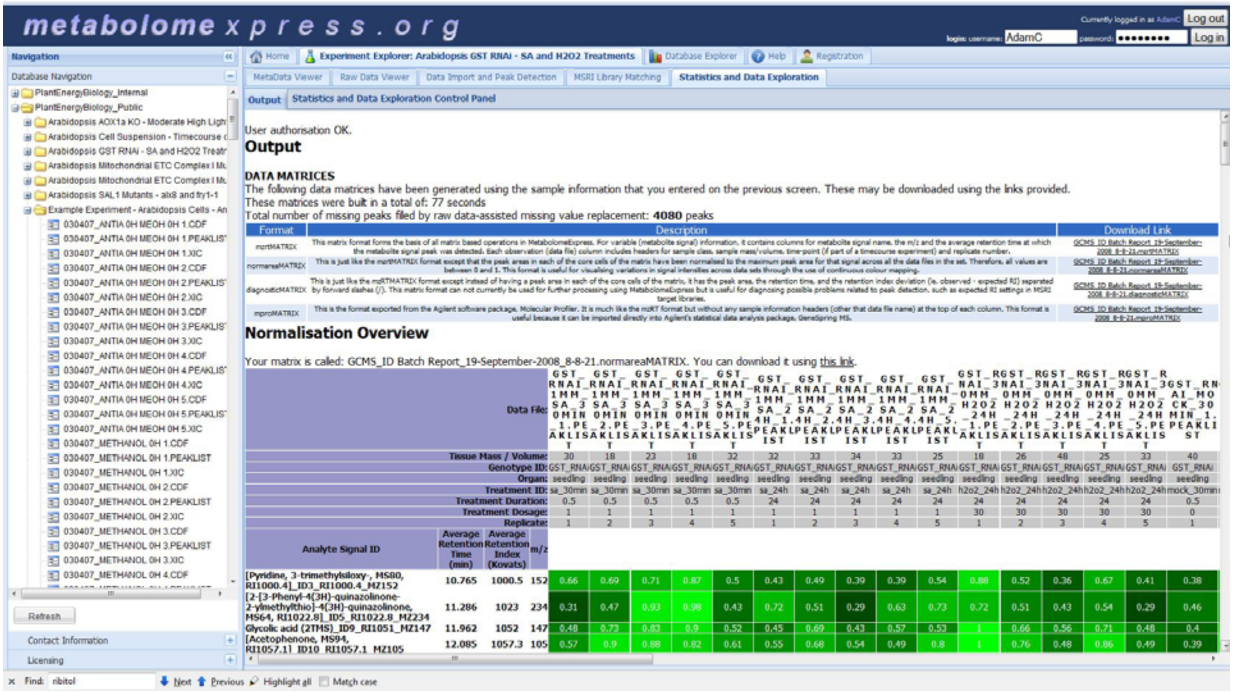

Once you have generated a matrix in this way, you can either apply another metadata-based normalisation or move straight on to some multivariate analysis, statistics or correlation analysis.

6.2 Matrix renormalisation

By default, all .mzrtMATRIX format data matrices are already normalised to internal standard peak intensity and an appropriate biomass/volume correction factor. The data matrix renormalisation tool let's you automatically renormalise .mzrtMATRIX format data matrices using metadata stored in the tab-delimited metadata file kept in the experiment folder with all the other raw and processed experimental data. Currently, there is only one type of renormalisation available, but more will be added in the future. The currently available method is to normalise each metabolite abundance value to the mean of its experimental control values. Appropriate controls are defined in the metadata file and cannot be changed except by editing that file. If you want to see which sample classes are normalised to which other sample classes, take a look at the table under the heading "GxE COMPARISONS". When you renormalise a matrix, any value belonging to a sample class defined by the numerator parameters (genotype, organ and treatment) will be divided by the mean of values from samples of the class defined by the associated denominator parameters (genotype, organ and treatment). If you wish to experiment with your own methods of renormalising raw .mzrtMATRIX data matrices using local software, you can load a matrix with the Matrix Explorer and download it as a spreadsheet-readable tab-delimited text file using the provided hyperlink. Anyway, set the "Data Matrix Renormalisation" control panel up as shown below and hit the "GO!" button.

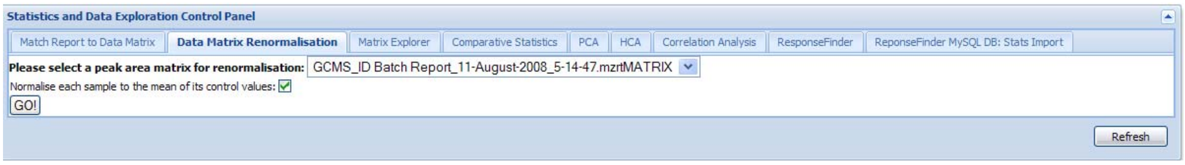

Wait a few moments, and you will be presented with the renormalised matrix displayed as an interactive heatmap, like this:

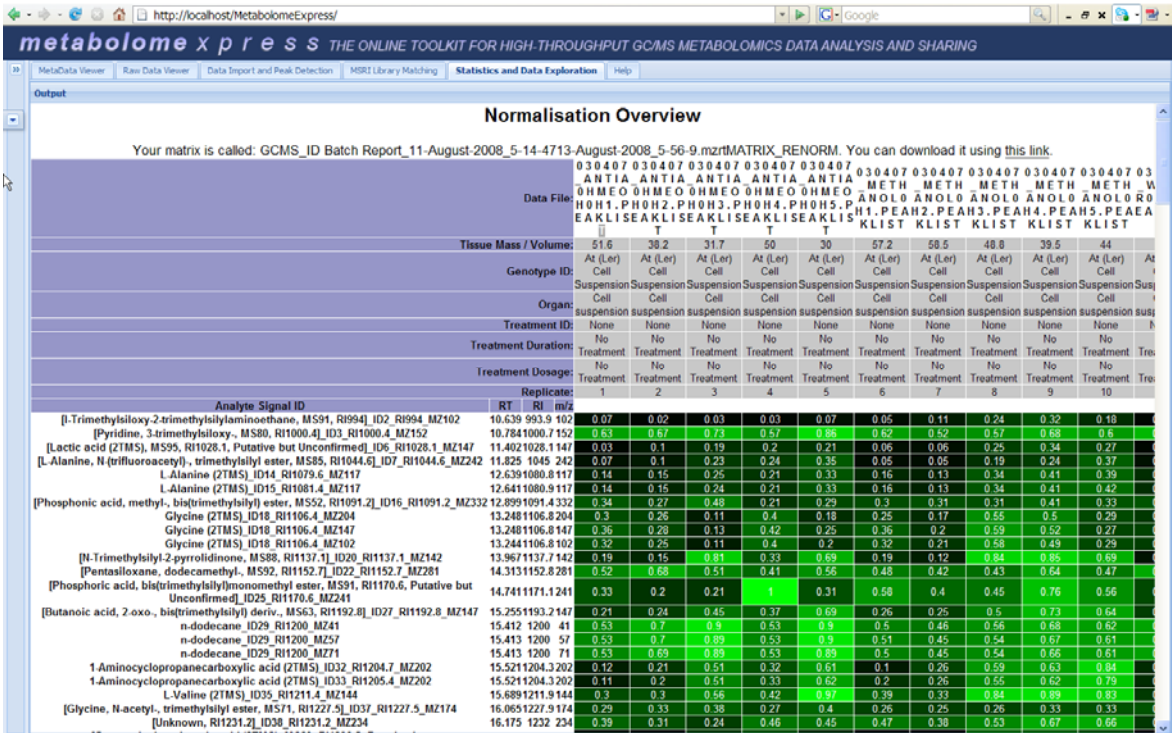

You can download the resulting matrix using the hyperlink at the top, or you can make a note of the name of the matrix, hit the "Refresh" button on the Statistics and Data Exploration Control Panel and it will appear in the selection boxes of all the relevant tools.

6.3 Using the interactive matrix explorer

Before we move on to the other tools, there is a feature of the matrix display that you should be aware of. If you click on the file name headers at the top of the matrix, you can make their background color cycle through blue, red and back to white. This allows you to select interesting samples out of the matrix so that if you double click on an analyte signal name (the left-most column), you can be taken straight to a chromatographic overlay of that analyte's quantifier peak in the selected files. Whether a column header is set to either blue or red in the matrix view will determine whether the corresponding GC/MS signal is plotted in either blue or red in the Raw Data Viewer. Columns with white headers will not be plotted. Below is an example:

Select the headers so they look like this... here we have set the 3 hour Antimycin A treated samples to red and the 3 hour methanol treated control samples to blue.

050407_ANTIA3HMEOH3H1.PEAKLIST	050407_ANTIA3HMEOH3H2.PEAKLIST	050407_ANTIA3HMEOH3H3.PEAKLIST	050407_ANTIA3HMEOH3H4.PEAKLIST	050407_ANTIA3HMEOH3H5.PEAKLIST	050407_METHANOL3H1.PEAKLIST	050407_METHANOL3H2.PEAKLIST	050407_METHANOL3H3.PEAKLIST	050407_METHANOL3H5.PEAKLIST
48.1	50.5	38.8	34.8	34.8	55	54.7	49	42.6
At (Ler) Cell Suspension	At (Ler) Cell Suspension	At (Ler) Cell Suspension	At (Ler) Cell Suspension	At (Ler) Cell Suspension	At (Ler) Cell Suspension	At (Ler) Cell Suspension	At (Ler) Cell Suspension	At (Ler) Cell Suspension
Cell suspension	Cell suspension	Cell suspension	Cell suspension	Cell suspension	Cell suspension	Cell suspension	Cell suspension	Cell suspension
AntimycinA_3h	AntimycinA_3h	AntimycinA_3h	AntimycinA_3h	AntimycinA_3h	Methanol_3h	Methanol_3h	Methanol_3h	Methanol_3h
3h	3h	3h	3h	3h	3h	3h	3h	3h
1	1	1	1	1	1	1	1	1
1	2	3	4	5	1	2	3	4
0.01	0.3	0.09	0.06	0.01	0.1	0.09	0.03	0.07
0.83	0.68	0.85	0.92	1	0.63	0.59	0.65	0.72
0.16	0.19	0.29	0.44	0.12	0.05	0.11	0.05	0.28
0.19	0.12	0.17	0.33	0.4	0.09	0.11	0.08	0.5
0.29	0.42	0.24	0.27	0.17	0.17	0.23	0.1	0.27
0.29	0.42	0.24	0.27	0.17	0.17	0.23	0.1	0.26
0.43	0.54	0.67	0.67	1	0.2	0.14	0.13	0.36
0.37	0.83	0.22	0.56	0.38	0.19	0.15	0.11	0.29
0.6	0.65	0.48	0.46	0.68	0.32	0.18	0.14	0.37
0.62	0.6	0.45	0.42	0.65	0.26	0.15	0.12	0.34
0.29	0.25	0.15	0.17	0.28	0.53	0.56	0.3	0.48
0.68	0.52	0.69	0.75	0.67	0.47	0.47	0.51	0.56
0.82	0.67	0.2	0.21	0.29	0.49	0.42	0.17	0.25
0.59	0.53	0.42	0.63	0.64	0.31	0.38	0.26	0.54
0.73	0.67	0.89	0.99	0.92	0.55	0.51	0.63	0.69
0.72	0.65	0.89	0.99	0.92	0.55	0.51	0.63	0.69
0.71	0.65	0.89	0.99	0.91	0.54	0.51	0.63	0.69
0.31	0.56	0.42	0.37	0.19	0.23	0.54	0.35	0.5
0.31	0.55	0.42	0.37	0.19	0.23	0.54	0.35	0.5
0.72	0.79	0.44	0.62	0.71	0.46	0.72	0.37	0.63
0.45	0.41	0.61	0.67	0.67	0.23	0.24	0.3	0.42
0.27	0.41	0.2	0.16	0.23	0.45	0.5	0.4	0.55

Now scroll back to the analyte signal name column and double click one of the signal names.

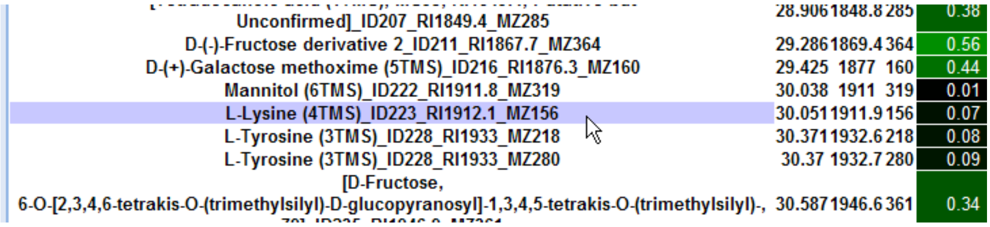

Now wait a moment and you will be automatically taken to the relevant chromatographic overlay in the *Raw Data Viewer* like this:

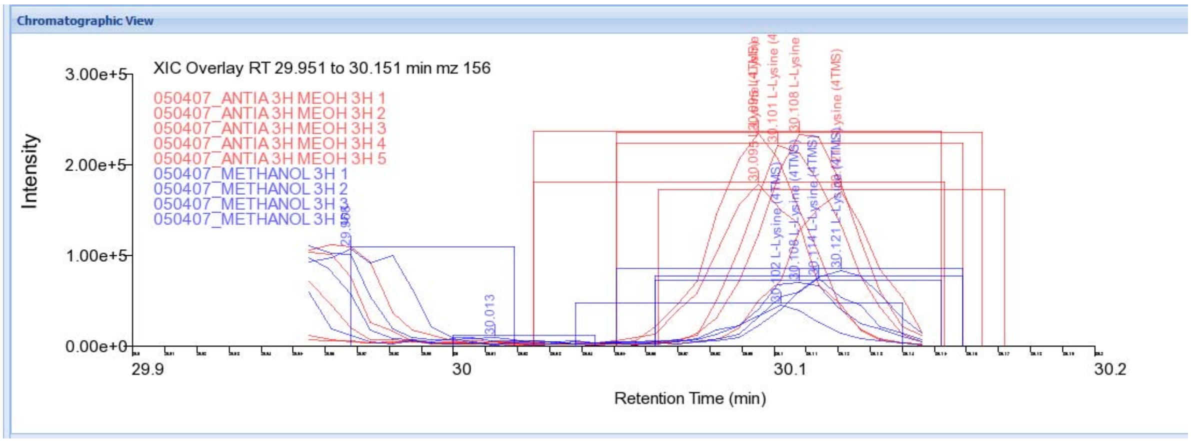

This makes it really easy to compare heatmap values with the raw signals and peak detections that underlie them.

The "Matrix Explorer" loads any data matrix into an interactive heatmap in the same way as illustrated above without carrying out any renormalisation, so now, let's move on to the "Comparative Statistics" tool.

6.4 Comparative statistics

This tool provides you with the ability to select a data matrix and then select some sample class comparisons to make (by Welch's t-test) before being presented with an interactive heatmap of fold-differences and p-values. The heatmap is linked to the raw data viewer in that if you double-click on a cell containing a fold-difference, you will be automatically taken to the Raw Data Viewer and presented with the underlying GC/MS signals. This is great for building up confidence in particular results!

Here is an example:

Go to the "Comparative Statistics" tool, select a data matrix and hit the "Upload" button.

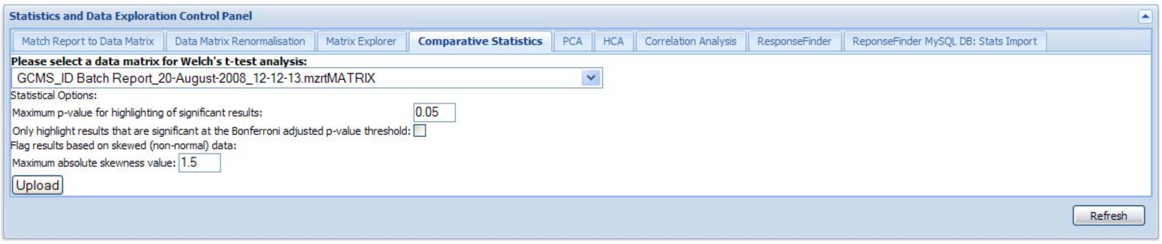

Wait a moment and you will be presented with a selection box asking you to choose your sample class comparisons of interest, like this:

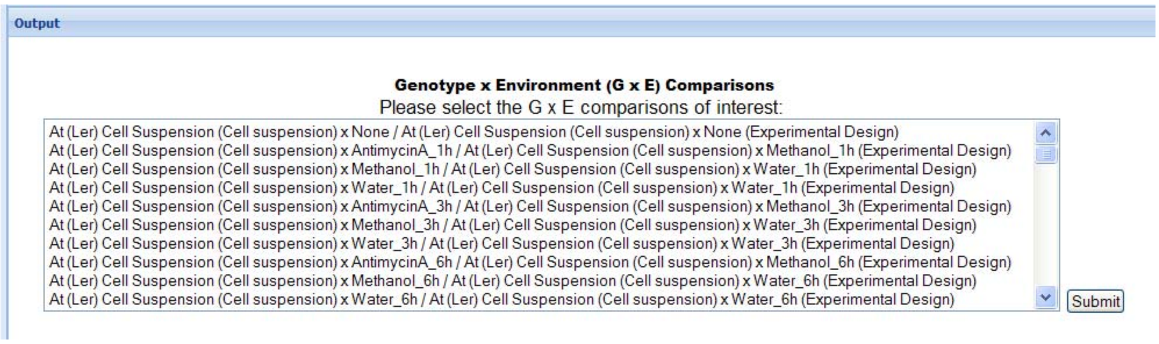

You will notice that a number of the possible options at the top of the list are marked with the label "(Experimental Design)" on the end. These are the class comparisons that reflect the original design of the experiment as intended by the authors (ie. treatment vs. control). If you choose from these, you know you are looking at results that are directly related to the original hypothesis behind the experiment.

Now, select the Experimental Design comparisons with Antimycin A sample classes as the numerator and hit the "Submit" button. Wait a moment and you will be presented with a heatmap statistical table like the one shown below. You can sort the rows of the table according to the values in any column by clicking on the corresponding column header. The image below shows the table after being sorted first by the signal intensity ratio of Antimycin A treated cells vs Methanol treated cells at 12 h and then by 'Chemical Class'.

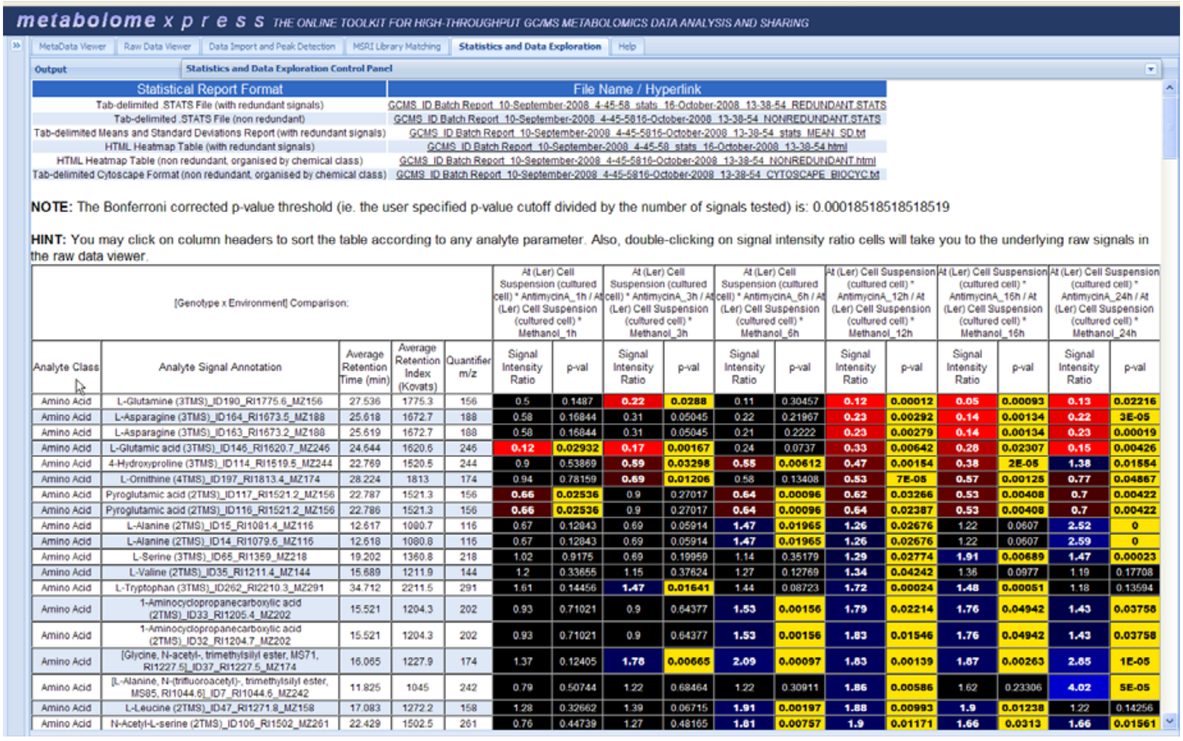

Statistical Report Format	File Name / Hyperlink
Tab-delimited .STATS File (with redundant signals)	GCMS_ID Batch Report_10-September-2008_4-45-58_stats_16-October-2008_13-38-54_REDUNDANT.STATS
Tab-delimited .STATS File (non redundant)	GCMS_ID Batch Report_10-September-2008_4-45-5816-October-2008_13-38-54_NONREDUNDANT.STATS
Tab-delimited Means and Standard Deviations Report (with redundant signals)	GCMS_ID Batch Report_10-September-2008_4-45-5816-October-2008_13-38-54_stats_MEAN_SD.txt
HTML Heatmap Table (with redundant signals)	GCMS_ID Batch Report_10-September-2008_4-45-58_stats_16-October-2008_13-38-54.html
HTML Heatmap Table (non redundant, organised by chemical class)	GCMS_ID Batch Report_10-September-2008_4-45-5816-October-2008_13-38-54_NONREDUNDANT.html
Tab-delimited Cytoscape Format (non redundant, organised by chemical class)	GCMS_ID Batch Report_10-September-2008_4-45-5816-October-2008_13-38-54_CYTOSCAPE_BIOCYC.txt

NOTE: The Bonferroni corrected p-value threshold (ie. the user specified p-value cutoff divided by the number of signals tested) is: 0.00018518518518519

HINT: You may click on column headers to sort the table according to any analyte parameter. Also, double-clicking on signal intensity ratio cells will take you to the underlying raw signals in the raw data viewer.

[Genotype x Environment] Comparison:					At (Ler) Cell Suspension (cultured cell) * AntimycinA_1h / At (Ler) Cell Suspension (cultured cell) * Methanol_1h		At (Ler) Cell Suspension (cultured cell) * AntimycinA_3h / At (Ler) Cell Suspension (cultured cell) * Methanol_3h		At (Ler) Cell Suspension (cultured cell) * AntimycinA_6h / At (Ler) Cell Suspension (cultured cell) * Methanol_6h		At (Ler) Cell Suspension (cultured cell) * AntimycinA_12h / At (Ler) Cell Suspension (cultured cell) * Methanol_12h		At (Ler) Cell Suspension (cultured cell) * AntimycinA_16h / At (Ler) Cell Suspension (cultured cell) * Methanol_16h		At (Ler) Cell Suspension (cultured cell) * AntimycinA_24h / At (Ler) Cell Suspension (cultured cell) * Methanol_24h	
Analyte Class	Analyte Signal Annotation	Average Retention Time (min)	Average Retention Index (Kovats)	Quantifier m/z	Signal Intensity Ratio	p-val	Signal Intensity Ratio	p-val	Signal Intensity Ratio	p-val	Signal Intensity Ratio	p-val	Signal Intensity Ratio	p-val	Signal Intensity Ratio	p-val
Amino Acid	L-Glutamine (3TMS)_ID190_RI1775.6_MZ156	27.536	1775.3	156	0.5	0.1487	0.22	0.0288	0.11	0.30457	0.12	0.00012	0.05	0.00093	0.13	0.02216
Amino Acid	L-Asparagine (3TMS)_ID164_RI1673.5_MZ188	25.618	1672.7	188	0.58	0.16844	0.31	0.05045	0.22	0.21967	0.23	0.00292	0.14	0.00134	0.22	3E-05
Amino Acid	L-Asparagine (3TMS)_ID163_RI1673.2_MZ188	25.619	1672.7	188	0.58	0.16844	0.31	0.05045	0.21	0.2222	0.23	0.00279	0.14	0.00134	0.23	0.00019
Amino Acid	L-Glutamic acid (3TMS)_ID146_RI1620.7_MZ246	24.644	1620.6	246	0.12	0.02932	0.17	0.00167	0.24	0.0737	0.33	0.00642	0.28	0.02307	0.15	0.00426
Amino Acid	4-Hydroxyproline (3TMS)_ID114_RI1519.5_MZ244	22.769	1520.5	244	0.9	0.53869	0.59	0.03298	0.55	0.00612	0.47	0.00154	0.38	2E-05	1.38	0.01554
Amino Acid	L-Ornithine (4TMS)_ID197_RI1813.4_MZ174	28.224	1813	174	0.94	0.78159	0.69	0.01206	0.58	0.13408	0.53	7E-05	0.57	0.00125	0.77	0.04867
Amino Acid	Pyroglutamic acid (2TMS)_ID117_RI1521.2_MZ156	22.787	1521.3	156	0.66	0.02536	0.9	0.27017	0.64	0.00096	0.62	0.03266	0.53	0.00408	0.7	0.00422
Amino Acid	Pyroglutamic acid (2TMS)_ID116_RI1521.2_MZ156	22.786	1521.3	156	0.66	0.02536	0.9	0.27017	0.64	0.00096	0.64	0.02387	0.53	0.00408	0.7	0.00422
Amino Acid	L-Alanine (2TMS)_ID15_RI1081.4_MZ116	12.617	1080.7	116	0.67	0.12843	0.69	0.05914	1.47	0.01965	1.26	0.02676	1.22	0.0607	2.52	0
Amino Acid	L-Alanine (2TMS)_ID14_RI1079.6_MZ116	12.618	1080.8	116	0.67	0.12843	0.69	0.05914	1.47	0.01965	1.26	0.02676	1.22	0.0607	2.59	0
Amino Acid	L-Serine (3TMS)_ID65_RI1359_MZ218	19.202	1360.8	218	1.02	0.9175	0.69	0.19959	1.14	0.35179	1.29	0.02774	1.91	0.00689	1.47	0.00023
Amino Acid	L-Valine (2TMS)_ID35_RI1211.4_MZ144	15.689	1211.9	144	1.2	0.33655	1.15	0.37624	1.27	0.12769	1.34	0.04242	1.36	0.0977	1.19	0.17708
Amino Acid	L-Tryptophan (3TMS)_ID262_RI2210.3_MZ291	34.712	2211.5	291	1.61	0.14456	1.47	0.01641	1.44	0.08723	1.72	0.00024	1.48	0.00051	1.18	0.13594
Amino Acid	1-Aminocyclopropanecarboxylic acid (2TMS)_ID33_RI1205.4_MZ202	15.521	1204.3	202	0.93	0.71021	0.9	0.64377	1.53	0.00156	1.79	0.02214	1.76	0.04942	1.43	0.03758
Amino Acid	1-Aminocyclopropanecarboxylic acid (2TMS)_ID32_RI1204.7_MZ202	15.521	1204.3	202	0.93	0.71021	0.9	0.64377	1.53	0.00156	1.83	0.01546	1.76	0.04942	1.43	0.03758
Amino Acid	[Glycine, N-acetyl-, trimethylsilyl ester, MS71, RI1227.5]_ID37_RI1227.5_MZ174	16.065	1227.9	174	1.37	0.12405	1.78	0.00665	2.09	0.00097	1.83	0.00139	1.87	0.00263	2.85	1E-05
Amino Acid	[L-Alanine, N-(trifluoroacetyl)-, trimethylsilyl ester, MS85, RI1044.6]_ID7_RI1044.6_MZ242	11.825	1045	242	0.79	0.50744	1.22	0.68464	1.22	0.30911	1.86	0.00586	1.62	0.23306	4.02	5E-05
Amino Acid	L-Leucine (2TMS)_ID47_RI1271.8_MZ158	17.083	1272.2	158	1.28	0.32662	1.39	0.06715	1.91	0.00197	1.88	0.00993	1.9	0.01238	1.22	0.14256
Amino Acid	N-Acetyl-L-serine (2TMS)_ID106_RI1502_MZ261	22.429	1502.5	261	0.76	0.44739	1.27	0.48165	1.81	0.00757	1.9	0.01171	1.66	0.0313	1.66	0.01561

You can download statistical reports in various formats using the hyperlinks at the top of the page. If you see an interesting statistical result, try double-clicking on the analyte abundance ratio to see the underlying GC/MS signals.

6.5 Principal Components Analysis (PCA)

The Metabolome Express PCA tool provides you with the ability to very quickly find interesting patterns in datasets on the server. The PCA tool utilises sample class information contained in the experimental metadata file to provide publication-quality 2D and 3D PCA score plots, together with sortable, variable loadings heatmaps and scree plots. All you have to do is select a matrix, specify whether you want to include redundant signals (repeat measurements of the same metabolite but using different GC/MS signals - two different quantifier ions for example) and whether to include "unknown" metabolites that have not had there structures verified by comparison with authentic standards. You will then be given a chance to select which samples to include in the PCA. By default, all variables are scaled to unit variance before the PCA is carried out.

For this example, you don't need to change anything, just hit the 'Upload' button.

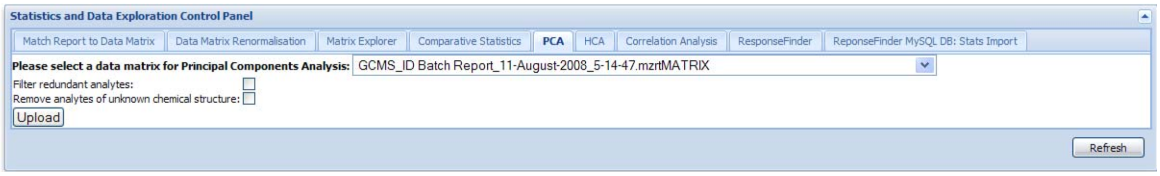

You will then be given a chance to select which samples you wish to include in the PCA:

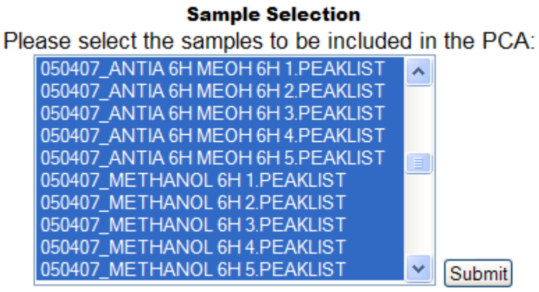

For example, try selecting the 6-hour Antimycin A and Methanol treated samples and then hit 'Submit'.

Wait a moment and the following items will appear in the Output window:

The table at the top provides you with hyperlinks to download the input and output files:

PCA Results File	File Name / Hyperlink
Tab-delimited PCA Input Data Matrix (filtered)	GCMS_ID Batch Report_11-August-2008_5-14-47.mzrtMATRIX_FILTERED_1218681255_375
Tab-delimited PCA Input Data Matrix - Transposed and Simplified (R Importable)	GCMS_ID Batch Report_11-August-2008_5-14-47.mzrtMATRIX_FILTERED_1218681255_375_TRANSPOSED
Tab-delimited PCA Scores Table	GCMS_ID Batch Report_11-August-2008_5-14-47.mzrtMATRIX_FILTERED_1218681255_375_TRANSPOSED_PCASCORES
Tab-delimited Variable Loadings Table	GCMS_ID Batch Report_11-August-2008_5-14-47.mzrtMATRIX_FILTERED_1218681255_375_TRANSPOSED_PCALOADINGS
Tab-delimited PC Standard Deviations Table	GCMS_ID Batch Report_11-August-2008_5-14-47.mzrtMATRIX_FILTERED_1218681255_375_TRANSPOSED_PCASD

A 2D PCA Score Plot in Portable Network Graphics (.PNG) format showing the first two principal components:

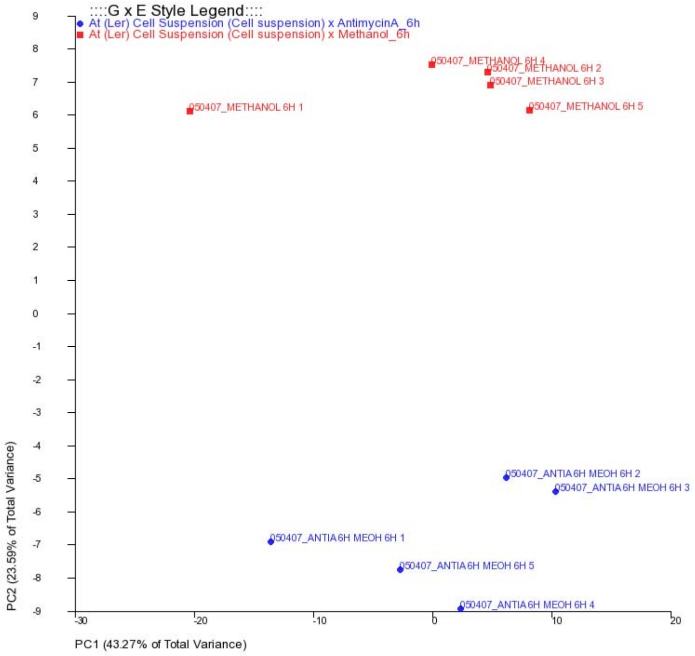

This plot shows that PC2 contains most of the interesting biological variance related to the Antimycin A treatment.

An identical copy of the plot is also provided in Scalable Vector Graphics (.SVG) format for editing in a compatible drawing program like Adobe Illustrator.

Below these plots you will see a scree plot:

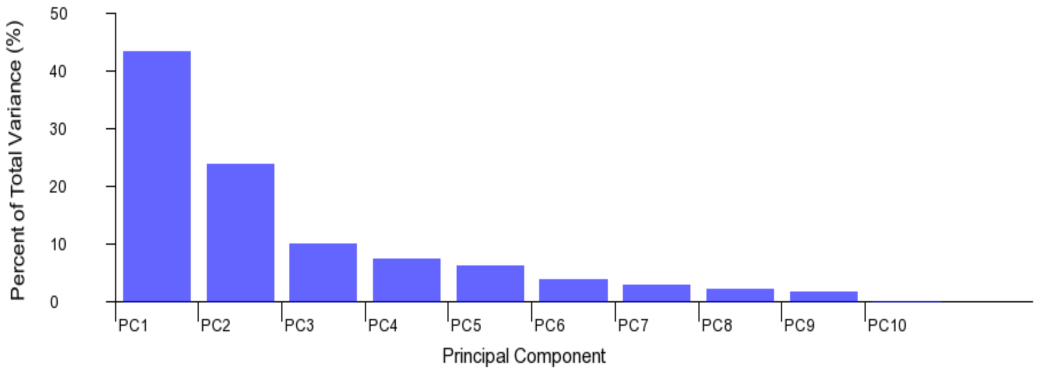

Below the scree plot, you will see a variable loadings heatmap. You can sort the columns by clicking on the headers. Here we have sorted by the most biologically interesting component, PC2. You will find that the metabolites with the highest absolute loading values in principal components that give good separation between two biological classes will also be the most significantly different ones when a t-test is used to compare those classes.

VARIABLE LOADINGS

Hint: Click on the PC column headers to sort table contents

	PC1	PC2	PC3	PC4	PC5	PC6	PC7	PC8	PC9	PC10
[Unknown, RI1335.8]_ID61_RI1335.8_MZ285	-0.0041	-0.130	0.0142	-0.0015	-0.0208	-0.0628	0.0916	-0.0288	-0.0019	0.0511
[Unknown, RI1231.2]_ID38_RI1231.2_MZ234	0.028	-0.1244	-0.0632	0.0046	0.0015	0.043	0.0266	-0.0166	0.0137	0.0702
Nicotinic acid (1TMS)_ID55_RI1303.3_MZ180	-0.0297	-0.1239	-0.0214	-0.0552	-0.0402	-0.0058	0.0604	-0.0273	0.0105	0.0563
Glyceric acid (3TMS)_ID59_RI1330.8_MZ292	-0.0229	-0.1223	0.0402	-0.0105	-0.0631	0.0339	0.0006	-0.1124	0.0454	0.0236
L-Valine (2TMS)_ID35_RI1211.4_MZ144	-0.0181	-0.119	-0.0834	0.0113	0.0069	-0.0534	0.0631	0.0352	-0.0651	-0.1077
1-Aminocyclopropanecarboxylic acid (2TMS)_ID33_RI1205.4_MZ202	-0.0181	-0.119	-0.0834	0.0113	0.0069	-0.0534	0.0631	0.0352	-0.0651	0.1283
1-Aminocyclopropanecarboxylic acid (2TMS)_ID32_RI1204.7_MZ202	-0.0395	-0.1187	0.0272	0.0704	0.0093	-0.0015	0.0274	0.0136	0.0361	0.0592
[Sedoheptulose methoxime (6TMS), MS86, RI2116.9]_ID251_RI2116.9_MZ319	-0.0493	-0.1168	0.0191	0.0263	0.0422	0.0155	-0.0139	-0.0121	-0.009	0.0117
L-Proline (2TMS)_ID53_RI1300_MZ142	-0.0442	-0.1165	0.0481	0.0151	-0.0052	0.0392	0.0447	-0.0576	-0.0271	0.0161
[Unknown, RI2767.2]_ID311_RI2767.2_MZ361	-0.038	-0.1164	0.0722	-0.0039	0.0008	0.0369	-0.0498	0.0362	0.0139	-0.0387
D-Fructose-6-phosphate methoxime (6TMS) EZ Peak 1_ID265_RI2292.9_MZ315	-0.0427	-0.1148	0.0658	0.0174	0.0232	-0.016	-0.0481	0.0355	0.0228	-0.0069
beta-Alanine (3TMS)_ID85_RI1428.2_MZ248	-0.0039	-0.1142	-0.0562	0.0868	-0.055	-0.0005	0.0964	-0.0696	0.0223	-0.0443
L-Malic acid (3TMS)_ID99_RI1482.8_MZ233	-0.0039	-0.1142	-0.0562	0.0868	-0.055	-0.0005	0.0964	-0.0696	0.0223	-0.0443
L-Leucine (2TMS)_ID47_RI1271.8_MZ158	-0.044	-0.1131	0.0189	0.0115	0.089	-0.0275	0.0465	0.0015	-0.0296	-0.05
[L-Alanine, N-(trifluoroacetyl)-, trimethylsilyl ester, MS85, RI1044.6]_ID7_RI1044.6_MZ242	0.0081	-0.1103	0.0378	0.1018	-0.0181	-0.02	-0.1043	-0.1307	-0.0453	0.3087
[Unknown, RI1217.2]_ID36_RI1217.2_MZ147	-0.0457	-0.1079	0.0557	0.029	0.0792	-0.0371	0.0106	0.0366	0.0158	0.0073
L-Tyrosine (3TMS)_ID228_RI1933_MZ218	-0.046	-0.1075	0.0827	0.0149	-0.0044	-0.0074	-0.062	0.0385	0.0324	-0.0332
L-Threonine (3TMS)_ID72_RI1385.8_MZ291	0.0306	-0.1057	0.0113	0.0577	-0.116	0.0624	-0.0703	-0.0667	0.0115	-0.015
p-Aminobenzoic acid (2TMS)_ID205_RI1838.1_MZ281	0.0395	-0.1056	0.0053	0.0651	-0.0794	0.1031	0.0199	0.0637	0.0083	0.0586
L-Phenylalanine (2TMS)_ID148_RI1630.8_MZ218	-0.0515	-0.1004	-0.0281	0.0111	-0.0838	-0.0493	-0.0216	0.1149	0.0775	0.0258
Pyroglutamic acid (2TMS)_ID116_RI1521.2_MZ156	-0.0139	-0.1	0.0631	0.088	0.0967	0.0203	0.1322	0.0283	0.0139	0.0577
[Arabinofuranose, 1,2,3,5-tetrakis-O-(trimethylsilyl)-, MS70, RI1607.5]_ID143_RI1607.5_MZ217	0.0035	-0.0986	-0.0991	-0.05	-0.0988	0.0904	0.0206	0.0526	0.0652	0.0536
	-0.0461	-0.0972	0.055	0.0403	-0.0515	0.052	-0.1423	-0.0752	0.0078	0.0189
[Unknown, RI2656.2]_ID295_RI2656.2_MZ319	-0.0527	-0.0965	0.0355	-0.0139	-0.0694	0.0148	-0.1485	-0.0474	0.0157	-0.0545
[Unknown Possible Organic Acid, RI1411.1]_ID79_RI1411.1_MZ219	0.0059	-0.0932	0.0272	0.0988	-0.1264	0.1099	-0.0705	-0.0509	0.0406	-0.0226
[N-Trimethylsilyl-2-pyrrolidinone, MS88, RI1137.1]_ID20_RI1137.1_MZ142	0.0245	-0.0909	-0.132	-0.0668	-0.0116	0.0692	-0.0119	0.0301	0.0258	-0.2958
D-(-)-Arabinose methoxime (4TMS)_ID161_RI1659.6_MZ217	-0.0657	-0.0897	0.0202	-0.0729	-0.0485	-0.0278	0.0591	0.0272	-0.0261	0.0126
2-Aminoadipic acid (3TMS)_ID170_RI1717.3_MZ260	-0.0657	-0.0897	0.0202	-0.0729	-0.0485	-0.0278	0.0591	0.0272	-0.0261	0.0126
[Unknown, RI2731.2]_ID304_RI2731.2_MZ425	-0.0027	-0.0883	-0.1414	0.022	0.0439	0.0766	-0.0743	0.0735	-0.0487	-0.0433
L-Tryptophan (3TMS)_ID261_RI2210.3_MZ202	-0.0027	-0.0883	-0.1414	0.022	0.0439	0.0766	-0.0743	0.0735	-0.0487	-0.0433
[Raffinose (11TMS), MS94, Putative but Unconfirmed]_ID342_RI3341.3_MZ437	-0.0241	-0.0844	-0.1233	0.0371	0.0594	0.1062	-0.0856	0.0618	-0.0534	-0.1
L-Tyrosine (3TMS)_ID228_RI1933_MZ280	-0.0617	-0.0838	0.0867	0.0326	0.047	-0.0283	-0.0395	0.0848	0.0276	0.0109
[4-Ketoglucose, bis(O-methyloxime), tetrakis(trimethylsilyl), MS72, RI1463.6]_ID93_RI1463.6_MZ173	-0.0559	-0.0837	0.0532	-0.0541	0.1116	-0.0577	0.0453	0.0436	-0.0208	-0.0543

Finally, if you have an X3D compatible virtual-reality browser plug-in installed, you will see a 3D PCA Score Plot showing the first three PCs. Here's a screenshot showing what the example PCA looked like when viewed using the trial version of the BS Contact X3D viewer available for free from Bitmanagement Software. If you like, you can download the X3D files using the hyperlink at the top of the page and edit the 3D scene using an X3D compatible 3D editor (to create a nice powerpoint slide for example).

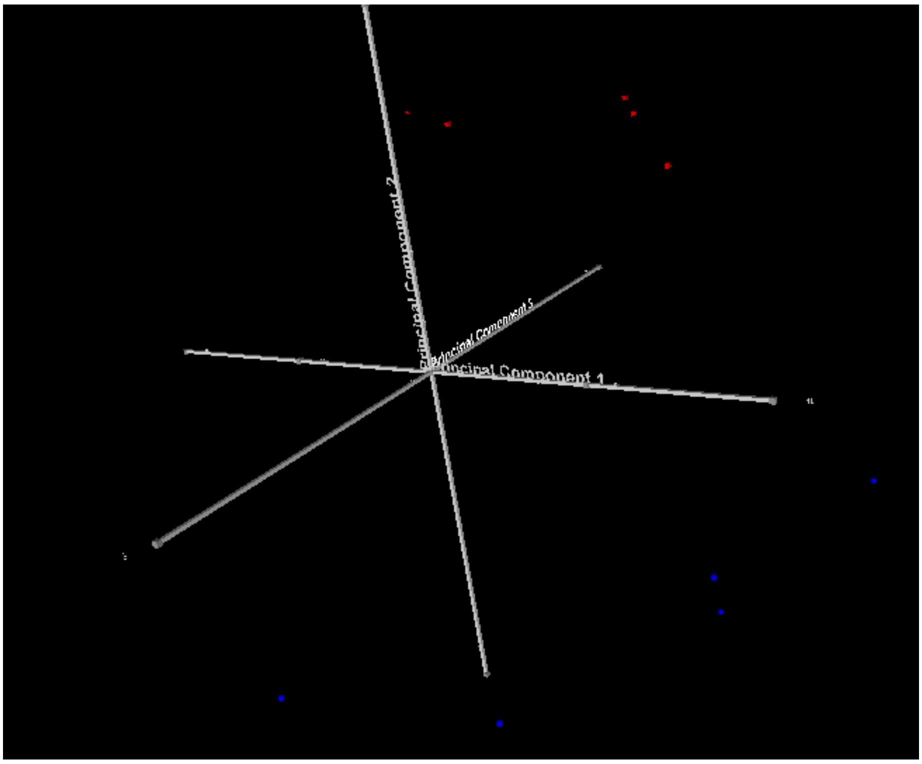

6.6 Hierarchical Cluster Analysis (HCA)

The HCA tool currently provides two modes of clustering:

1. Bi-clustering of the Metabolite x Sample Data Matrix, and

2. Bi-clustering of the Metabolite x Metabolite Correlation Matrix.

Both modes of clustering are performed using automatically generated R scripts that utilise the heatmap() function freely available as part of the Bioconductor open-source bioinformatics software package for R (see www.bioconductor.org). The output is generated as a PDF which is displayed directly in the Output window. You will therefore need to ensure you have installed a PDF viewer browser plugin if you wish to view the clustering results directly in the browser. Alternatively, you may wish to download the PDF document using the hyperlink at the top of the Output window and view the PDF using your normal PDF viewer.

To see a nice example, set the HCA control panel as shown below and hit 'Upload'.

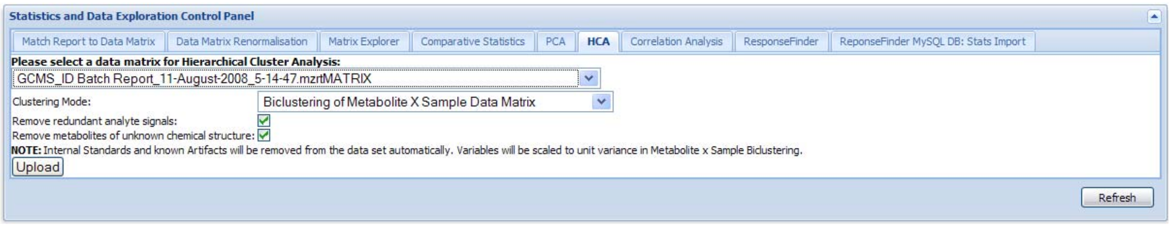

Then select the 24 hour Antimycin A and Methanol treated samples from the selection box and hit 'Submit'.

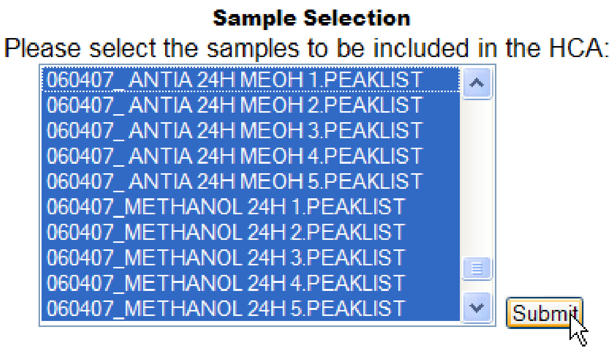

Wait a moment and, if you have your PDF viewer plugin installed correctly, you will be presented with a heatmap clustering like this:

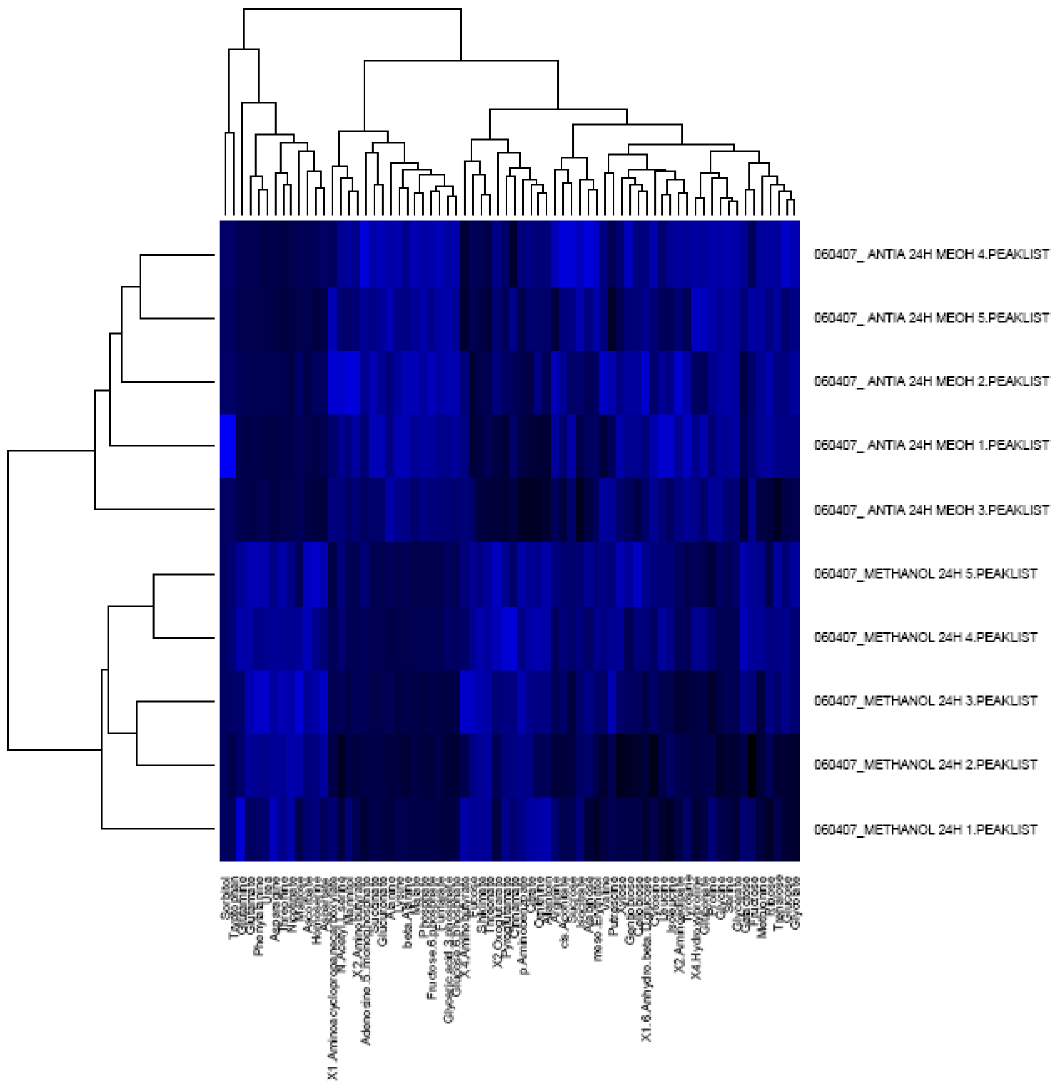

If you choose to do biclustering on the Metabolite x Metabolite Correlation Matrix, you will get a result like this:

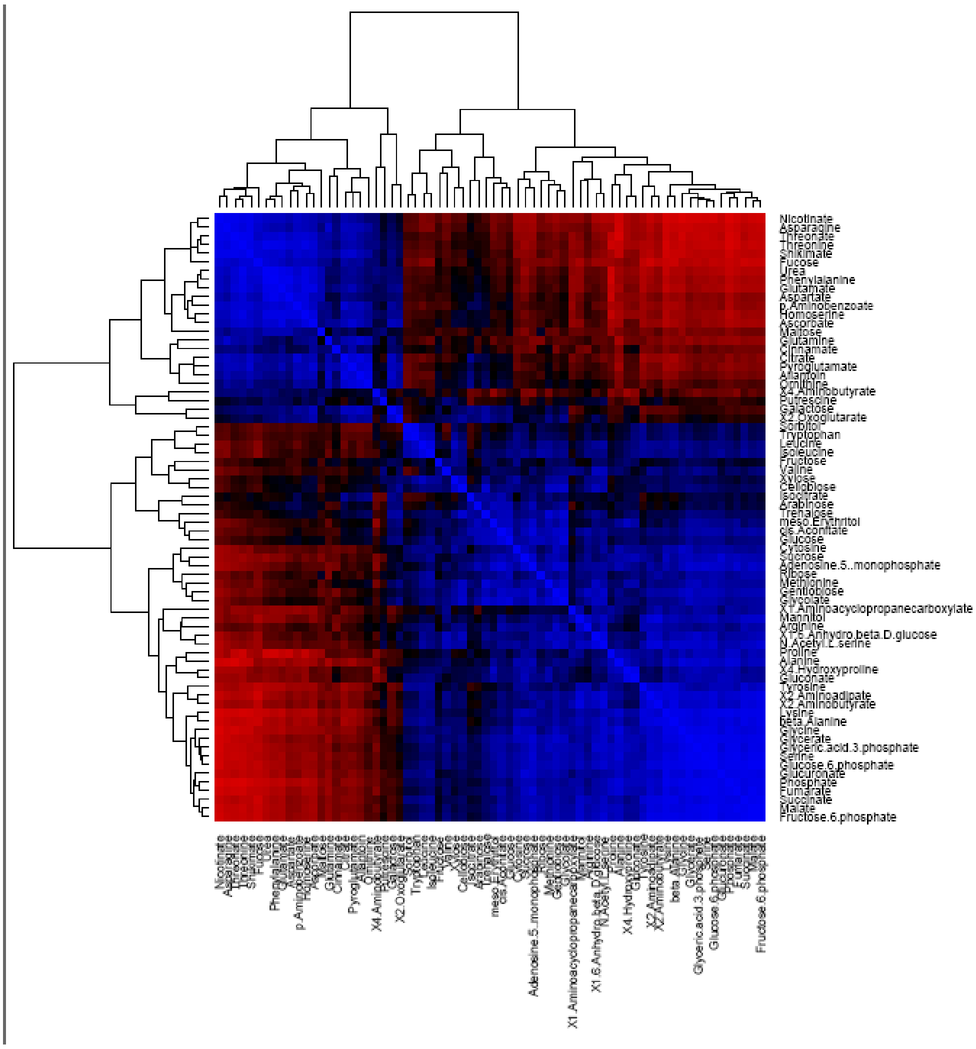

6.7 Correlation network construction

In addition to doing hierarchical bi-clustering of metabolite-metabolite correlation matrices, MetabolomeExpress also has a tool for generating correlation network graphs from data matrices. The tool is very simple. All you need to do is select a data matrix from the drop down list, specify a minimum correlation coefficient that two metabolites must exhibit in order for their nodes to be connected, and hit the 'Upload' button. The selected parameters will then be sent to the server and your network graphs will be constructed using all the values in the data matrix.

For the purposes of example, try setting the Correlation Analysis Control Panel up like this and hit 'Upload':

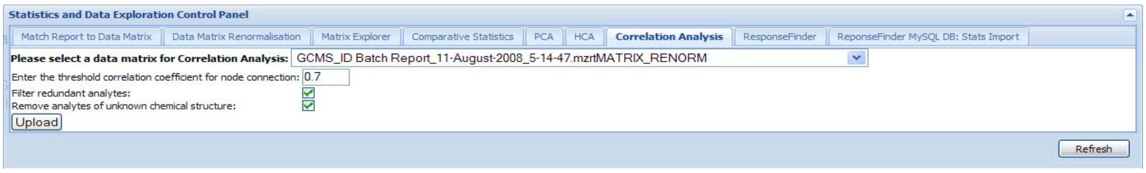

Wait a few moments while the correlations are calculated and the network graphs are constructed. You will then be presented with a page like this:

Output

Correlation Analysis: Progress

Reading Dataset...GCMS_ID Batch Report_11-August-2008_5-14-47.mzrtMATRIX_RENORM
Calculating Correlations |...

Output

CORRELATION NETWORKS
The following correlation network files have been generated. These may be downloaded using the links provided.

Format	Description	Download Link
Cytoscape	This format may be imported directly into the network visualisation and analysis software, Cytoscape. It contains all analyte-analyte correlations with absolute correlation coefficients above the threshold that was specified in the submitted request. It includes two attribute columns giving the absolute correlation coefficient and the sign of the correlation (ie. "Positive" or "Negative"). This allows the user to take advantage of Cytoscape's filtering and attribute-to-visual style mapping features.	GCMS_ID Batch Report_11-August-2008_5-14-47_CYTOSCAPE_CORNET_19-August-2008_4-35-21.TXT
Pajek	This format may be imported directly into the open-source network visualisation and analysis package, Pajek. It contains all analyte-analyte correlations above the specified threshold.	GCMS_ID Batch Report_11-August-2008_5-14-47_PAJEK_CORNET_19-August-2008_4-35-21.NET
Correlation Matrix (Tab-Delimited)	This format consists of a square tab-delimited correlation matrix giving all analyte-analyte correlation coefficients.	GCMS_ID Batch Report_11-August-2008_5-14-47_CORMAP_19-August-2008_4-35-21.TXT
Correlation Matrix (Colour-Mapped HTML Table)	This is the same correlation matrix as described above, except that the cells containing correlation coefficients have been colour-mapped so that positive correlations are blue, negative correlations are red, and the intensity of the colour is directly proportional to the strength of the correlation. It may be opened in MS Excel.	GCMS_ID Batch Report_11-August-2008_5-14-47_CORMAP_19-August-2008_4-35-21.HTML

You can download the various output files using the provided hyperlinks.

To import the Cytoscape compatible file into Cytoscape, go to the File > Import > Network from Table (Text/MS Excel) and set the dialog box up like this (HINT: be sure to unselect 'Space' as a delimiter under 'Text File Import Options' before trying to select columns):

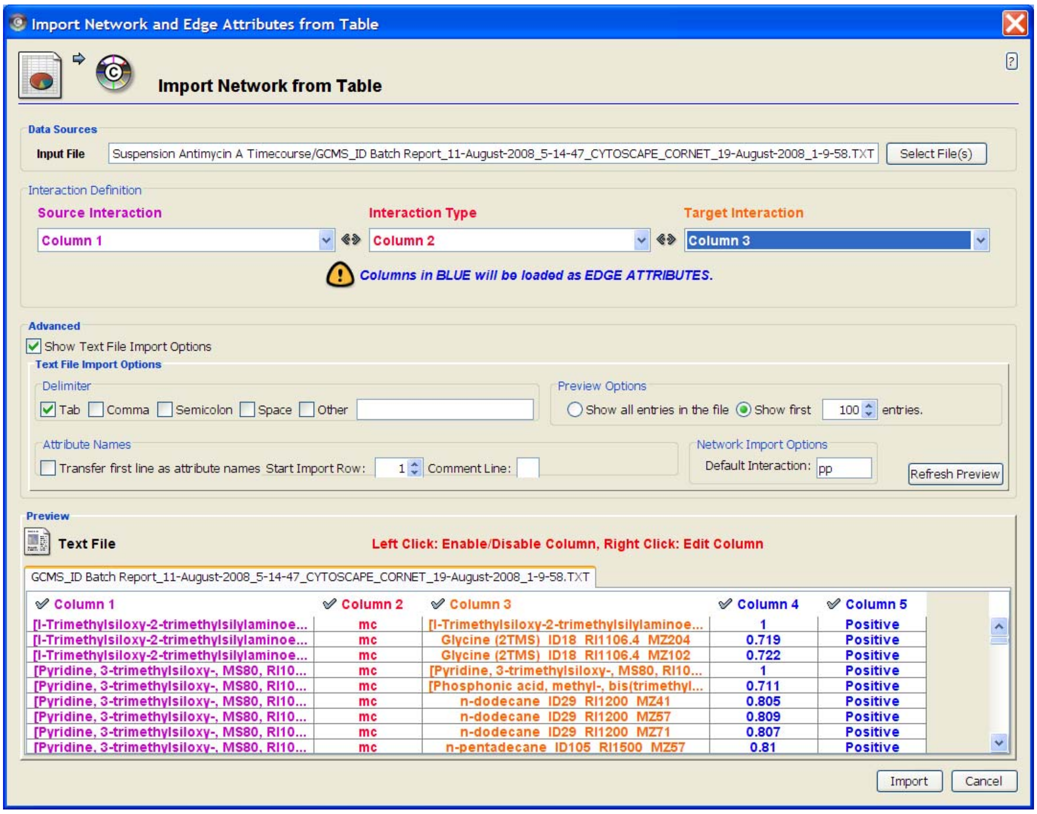

Here's what the example network looks like when imported into Cytoscape (ver 2.6.0) and laid out using the 'Organic' layout:

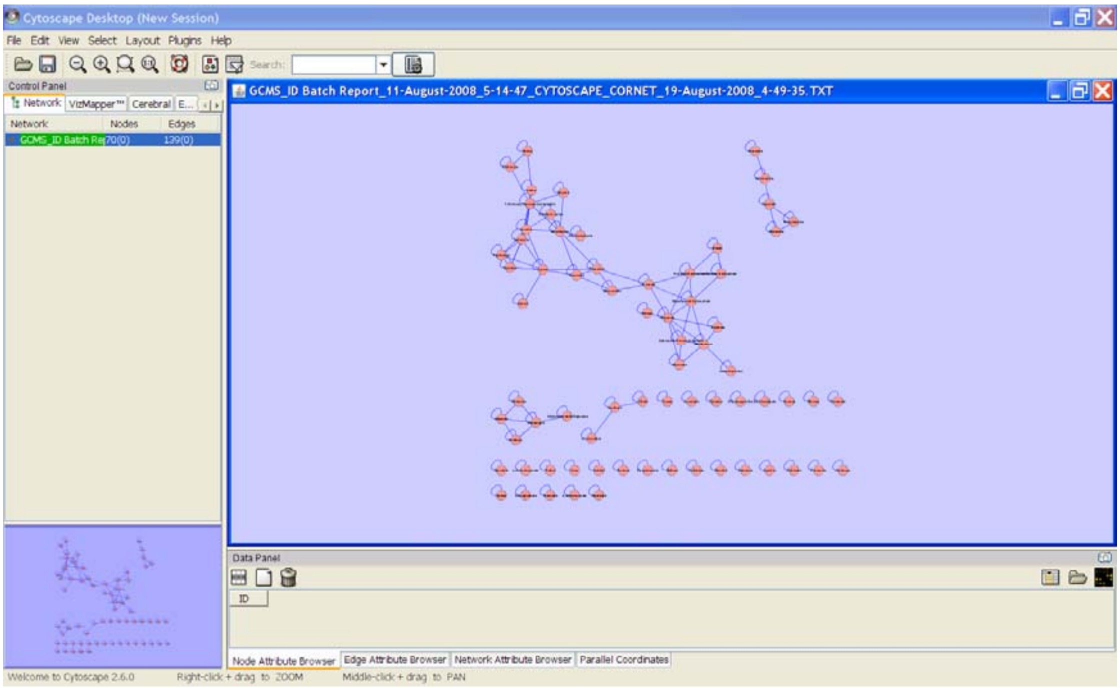

Here's a closeup:

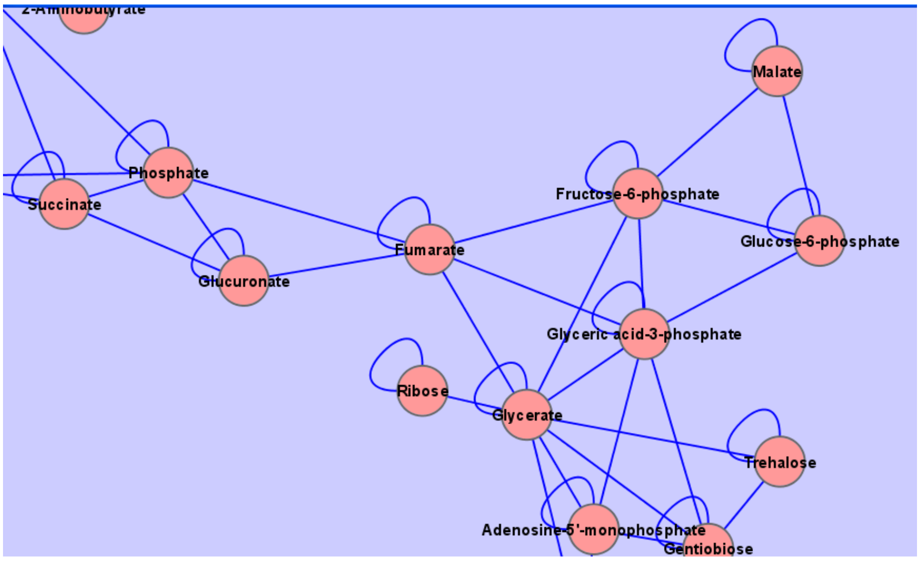

Notice the classic robust correlation between Fructose-6-P and Glucose-6-P in the top right corner.

6.8 Submitting a dataset to the main database of metabolite response statistics

Once you are happy with your data processing and have possibly even published your results, you are strongly encouraged to submit your dataset for indexing in the main database of metabolite response statistics. This will allow other researchers to use your data with the tools in *Database Explorer*.

To successfully submit your dataset, **your dataset will need to be in your public FTP repository folder and it will need to pass validation**.

Validation is done automatically by a computer script. The main purpose of validation is to check that all your raw and processed data files are present, that your dataset has been adequately described and that you have used the correct ontologies and string formats for your organism and research area. Therefore, you will need to complete all the required fields in your *_METADATA.TXT metadata file in accordance with the validation template file indicated for your area. The template applicable to your dataset should appear in the VERSION section of the metadata file you created during sample import.

NOTE: If you haven't seen your *_METADATA.TXT file before, check your experiment folder for a file with the name '*Name of Experiment Folder*_METADATA.TXT'. See the section in this manual on supported file formats for information about these files.

You can download the current validation templates from the Help page on the MetabolomeExpress website. You will need to refer to APPENDIX A in this manual to understand the templates. You should also refer to any minimal metadata reporting guidelines available for your research area to make sure the metadata values you provide meet these guidelines.

Datasets are submitted using the *ResponseFinder MySQL DB: Stats Import* tab of the *Statistics and Data Exploration Control Panel* (see below).

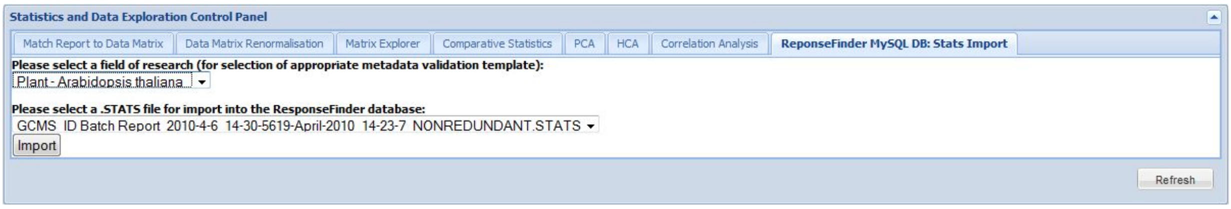

To submit your dataset, simply select the field of research corresponding to your metadata file version and the .STATS file containing the statistical results you wish to submit and then click the 'Import' button.

Your metadata file will then be checked against the appropriate template and if it passes validation, the MetabolomeExpress curator will be emailed with a notification of your wish to submit the dataset. A final manual security check will be conducted and, if passed, the dataset will soon be imported into the database. You will be notified by email as soon as this is done.

If your dataset does not pass validation, check the results panel for error messages indicating which fields are causing failure and fix your metadata file or required data files accordingly before trying again.

7 APPENDIX A – Interpretation of MetabolomeExpress Metadata Validation Templates

7.1 Background

Systematic annotation of biological datasets with contextual metadata adds great value to primary data by enabling the systematic analysis of relationships between contextual variables, technical parameters and biological phenotypes. Systematic annotation combines the use of defined data structures and controlled vocabularies (or ontologies) to facilitate computer-based processing of annotated data.

In MetabolomeExpress, annotation of datasets is achieved through the use of tab-delimited metadata (*_METADATA.TXT) files. Each experimental dataset in MetabolomeExpress is accompanied by a single metadata file providing answers to the following questions:

- Who did the experiment and how to contact them?
- What was the aim of the experiment?
- What were the genetic and phenotypic characteristics of the organisms studied?
- What environmental conditions were the organisms exposed to?
- What perturbations were applied to the organisms?
- What parts of the organisms were analysed?
- How were samples from the organisms processed and analysed?

The specific types of contextual information pertinent to a particular experiment and the range of possible values for each variable depend on the type of organism studied, the type of environment they were studied in and the type of experiment carried out with them. Minimum field sets appropriate to each research field are being determined by the Metabolomics Standards Initiative (MSI) and some recommendations have already been made in the form of 'roadmaps' for standard reporting in key metabolomics fields (http://msi-workgroups.sourceforge.net/).

To ensure that datasets contained in the main metabolite response database are annotated in a systematic and standardised manner that meets the recommendations of the MSI, *_METADATA.TXT files must be checked against an appropriate validation template before the associated datasets may be imported into the database. These validation templates define:

- The names of metadata fields
- Which fields are mandatory and which fields are optional
- The range of "allowed" values that a field may be assigned
- Written instructions for appropriate completion of each field.

7.2 Interpretation of MetabolomeExpress Metadata Template Files

Metadata *template* files have essentially the same structure as the MetabolomeExpress (*_METADATA.TXT) *metadata* format except special codes appear where metadata values would normally be. These codes define the acceptable

values that may be given for their respective fields. For example, the table below shows the `ADMINISTRATION` section of one metadata validation template.

MetabolomeExpress Experimental Metadata File - Mammalian Metabolomics (v1.0)	
**********ADMINISTRATION METADATA**********	
[ADMINISTRATION]	
Field	Value
Experiment Name:	[v]|vwn|1///Give the name of the experiment here
Project Name:	[v*]|vwn|1///Give the name of the bigger project of which this experiment is a part
Biological Experimentalist Name:	[v]|wv|2///Give the full name of the person who performed the biological part of the experiment
Biological Experimentalist Email:	[v]|e
Metabolome Analyst Name:	[v]|vv|2///Give the name of the person responsible for carrying out the metabolomic analysis
Metabolome Analyst Email:	[v]|e
Experimental Hypothesis:	[v]|vvn|10///Explain the hypothesis behind the experiment in at least 10 words.
Brief Description of Experiment:	[v]|vwn|30///Describe, in at least 30 words, how the experiment was carried out, including what other major pieces of data were acquired.
Literature Reference:	[v*]|vwn|5///If applicable, provide a literature reference (including full author list) to the article where this experiment has been published.
Journal:	[v*]|vwn|1///If applicable, give the unabbreviated name of the journal in which this experiment has been published
Publication Date (YYYY-MM-DD):	[v*]|d
PubMed ID:	[v*]|int

You can see the validation codes in the 'Value' column. The different codes will be explained below.

7.3 Validation Codes

The general code format is as follows:

`Validation level|data type|configuration parameters///Instructions for completion (optional)`

Validation Level

`Validation level` may be `[v]` or `[v*]`

`[v]` = field value must be present and must match the assigned data type and configuration parameters

`[v*]` = field value is optional. It may be blank, but if a value is present, it must match the assigned data type and configuration parameters

If neither of these validation level codes is present in the template field, then no validation of values for this field will occur.

7.4 Data types and associated configuration parameters

Float (f)

Data Type Code: `f`

Validation Description: Must be a number and the number may have a decimal point.

Configuration Parameters: None

Code Example: `[v]|f`

Example Valid Value: `1002.3652`

Example Invalid Value: `12defg`

Integer (int)

Data Type Code: `int`

Validation Description: Must be an integer

Configuration Parameters: None

Code Example: `[v]|int`

Example Valid Value: `1`

Example Invalid Value: `1.654`

Date (d)

Data Type Code: d

Validation Description: A date in YYYY-MM-DD format

Configuration Parameters: None

Example Code: `[v]|d`

Example Valid Value: `2010-01-01`

Example Invalid Value: `Jan 01 2010`

Time (t)

Data Type Code: `t`

Validation Description: A 24 h clock time in hh:mm:ss format

Configuration Parameters: None

Example Code: `[v]|t`

Example Valid Value: `11:59:59`

Example Invalid Value: `30:99:76`

Date/Time (dt)

Data Type Code: `dt`

Validation Description: A date time in YYYY-MM-DD hh:mm:ss format

Configuration Parameters: None

Example Code: `[v]|dt`

Example Valid Value: `2010-01-01 11:59:59`

Latitude and Longitude (latlong)

Data Type Code: `latlong`

Validation Description: A latitude and longitude in the latitude longitude format where latitude and longitude are each represented in the format *`degrees minutes seconds`* where *`degrees`* may be a +ve or -ve integer between -90 (S or W) and +90 (N or E), *`minutes`* is a +ve integer between 0 and 60 and *`seconds`* may be a +ve float between 0 and 60. For example, New York City is at around `40 42 51.36 -74 0 21.49`

Configuration Parameters: None

Example Code: `[v]|latlong`

Example Valid Value: `40 42 51.36 -74 0 21.49`

Email (e)

Data Type Code: e

Validation Description: A valid email address

Configuration Parameters: None

Example Code: `[v]|e`

Example Valid Value: `joe.bloggs@foo.com`

Example Invalid Value: `joe.bloggs(at)foo.com`

Name (name)

Data Type Code: `name`

Validation Description: A variable number of words with at least 2 words. Only letters are allowed – not numbers.

Configuration Parameters: None

Example Code: `[v]|name`

Example Valid Values: “`John Smith`”, “`John R. Smith`”, “`The Artist Formerly Known as Prince`”

Example Invalid Values: “`John`”, “`Prince`”, ”`John Smith_1`”

URL (url)

Data Type Code: `url`

Validation Description: A valid URL

Configuration Parameters: None

Example Code: `[v]|url`

Example Valid Value: `http://www.ncbi.nlm.nih.gov/pubmed/`

Example Invalid Value: `http:/www.ncbi.nlm.nih.gov/pubmed/`

Variable Words (vw)

Data Type Code: `vw`

Validation Description: A variable number of words with at least the minimum number of words. Numbers are allowed but not counted as words.

Configuration Parameters: An integer specifying the minimum number of words

Example Code: `[v]|vw|3`

Example Valid Values: “`Blue is beautiful`”, “`Blue is really beautiful`”

Example Invalid Values: “`Blue`”, “`Blue is`”, ”`John Smith_1`”

Variable Words (Row Release) (vw_rr)

Data Type Code: `vw_rr`

Validation Description: A variable number of words with at least the minimum number of words with a particular value releasing any further metadata requirements for the fields to the right of this field in the row. Numbers are allowed but not counted as words.

Configuration Parameters: `x,release_word` where `x` = An integer specifying the minimum number of words and `release_word` = a word that, if found it this field, releases any requirements for metadata in fields to the right of this field in the row.

Example Code: `[v]|vw_rr|1,WT`

Example Valid Values: "`WT`", "`Blue is really beautiful`"

Example Invalid Values: "`1`"

Variable Words or Numbers (vwn)

Data Type Code: `vwn`

Validation Description: A variable number of words with at least the minimum number of words. Numbers are allowed and counted as words.

Configuration Parameters: An integer specifying the minimum number of words

Example Code: `[v]|vwn|2`

Example Valid Values: "`Cabinet 1`", "`Cabinet A`", "`Cabinet A in room 106`", "`1234 9999`"

Example Invalid Values: "`1234`", "`Cabinet_1`", "`Cabinet_A`"

Species (sp)

Data Type Code: `sp`

Validation Description: A valid species name. Must be at least 2 words with no numbers. Species name will be checked against the NCBI Taxonomy database and a warning issued if the name is not recognised. Valid species names may be restricted to a particular branch of the taxonomic tree using an optional configuration parameter (explained below).

Configuration Parameters: Valid species names may be restricted to a particular branch of the NCBI taxonomic tree using an optional configuration parameter:

`NCBI_taxon_rank:value (eg. superkingdom:bacteria)`

Code Examples:

Example 1 `[v]|sp|///Enter any valid species name`

Example 2 `[v]|sp|superkingdom:bacteria///Enter the full name of any bacterial species`

Example 3 `[v]|sp|genus:saccharomyces///Enter the full name of any Saccharomyces sp.`

Example Valid Values:

Example 1 `Eucalyptus globulus`

Example 2 `Escherichia coli`

Example 3 `Saccharomyces cerevisiae`

Example Invalid Values:

Example 1 `E. globulus`

Example 2 `Homo sapiens`

Example 3 `Amanita phalloides`

Controlled Vocabulary (cv)

Data Type Code: `cv`

Validation Description: A single term that must match to a term specified in the configuration parameters

Configuration Parameters: A forward-slash (/)-separated list of allowed terms. A number of controlled vocabularies are built in to the MetabolomeExpress database and these may be included in the allowable term list by including as one of the allowable terms, their code name enclosed in square brackets (see code examples below). Code names for currently built-in controlled vocabularies are described in the table below:

Type of Entity	Name of Controlled Vocabulary	Description
Genes	`arabidopsis_genes`	Standard AGI convention names for genes of the model plant, *Arabidopsis thaliana* (eg. AT1G48030). Obtained from: ftp://ftp.arabidopsis.org/home/tair/Ontologies/Gene_Ontology/ATH_GO_GOSLIM.txt on 2010-04-01
	`human_genes`	Human gene symbols approved by the HUGO Gene Nomenclature Committee (eg. MDH1). Obtained from: http://www.genenames.org/cgi-bin/hgnc_stats.pl on 2010-04-01
	`mouse_genes`	Mouse gene marker symbols as per the Mouse Genome Informatics (MGI) website (eg. Mdh1). Obtained from: ftp://ftp.informatics.jax.org/pub/reports/gene_association.mgi on 2010-04-01
	`rice_genes`	Rice gene locus IDs as per the Rice Genome Annotation Project website (eg. LOC_Os07g43700). Obtained from: ftp://ftp.plantbiology.msu.edu/pub/data/Eukaryotic_Projects/o_sativa/annotation_dbs/pseudomolecules/version_6.1/all.dir/all.TU_model_brief_info.

		6.1 on 2010-04-02
	scerevisiae_genes	Systematic names of yeast (*Saccharomyces cerevisiae*) genes (eg. YDL078C) as per the Saccharomyces Genome Database (http://downloads.yeastgenome.org/chromosomal_feature/SGD_features.tab)
	ecoli_genes	Gene Names for genes in the *Escherichia coli* K-12 genome (eg. modB) as per the EcoGene database (http://www.ecogene.org/)
	dmelanogaster_genes	Genes symbols for genes in the *Drosophila melanogaster* genome as per the FlyBase FB2010_03 release version of the file gene_association.fb (http://flybase.org/static_pages/downloads/FB2010_03/go/gene_association.fb.gz)
Anatomy	plant_anatomy	Plant Ontology Consortium (http://www.plantontology.org) terms for plant structure. Obtained from: http://www.obofoundry.org/cgi-bin/detail.cgi?id=po_anatomy
	fungal_anatomy	Fungal Anatomy Ontology Project terms for fungal anatomy (http://www.yeastgenome.org/fungi/fungal_anatomy_ontology/#description). Obtained from: http://www.obofoundry.org/cgi-bin/detail.cgi?id=fungal_anatomy.
	human_anatomy	Human Developmental Anatomy Ontology, Abstract Version terms. Obtained from: http://www.obofoundry.org/cgi-bin/detail.cgi?id=human-dev-anat-abstract
	mouse_anatomy	Mouse Adult Gross Anatomy Ontology (http://www.informatics.jax.org/searches/AMA_form.shtml) terms. Obtained from: http://www.obofoundry.org/cgi-bin/detail.cgi?id=adult_mouse_anatomy
	fly_anatomy	FlyBase Drosophila Gross Anatomy Ontology terms for fly anatomy. Obtained from: http://www.obofoundry.org/cgi-bin/detail.cgi?id=fly_anatomy
Development	plant_development	Plant Ontology Consortium (www.plantontology.org) terms for plant growth and development. Obtained from: http://www.obofoundry.org/cgi-bin/detail.cgi?id=po_temporal.
	fly_development	FlyBase Drosophila Development Ontology terms for fly growth and development. Obtained from (http://www.obofoundry.org/cgi-bin/detail.cgi?id=fly_development)
Pathology	human_pathology	Human Disease Ontology (http://do-wiki.nubic.northwestern.edu/index.php/Main_Page) terms for human diseases. Obtained from: http://obo.cvs.sourceforge.net/*checkout*/obo/obo/ontology/phenotype/human_disease.obo
	mouse_pathology	Mouse Pathology Ontology (http://eulep.pdn.cam.ac.uk/Pathology_Ontology/index.php) terms for mouse pathologies. Obtained from: http://obo.cvs.sourceforge.net/*checkout*/obo/obo/ontology/phenotype/mouse_pathology/mouse_pathology.obo
Units of Measurement	units_time	Units of time without abbreviation (eg. 'milliseconds' NOT 'ms')
	units_percentconcentration	'% (v/v)' and '% (w/v)'
	units_molar	Molar units without abbreviation (eg. 'millimolar' NOT 'mM')
	units_mole	Mole units without abbreviation (eg. 'millimoles' NOT 'mmol')
	units_mass	Gram-based units of mass without abbreviation (eg. 'grams' NOT 'g')
	units_volume	Liter-based units of volume without abbreviation (eg. 'milliliters' NOT 'ml')
	units_tissuemass	Unabbreviated gram-based units of mass with a specification of either 'dry weight' or 'fresh weight' (eg. 'milligrams fresh weight' NOT 'mg FW')

Internal Reference (ir)

Data Type Code: `ir`

Validation Description: Field value must match with at least one value given for another metadata field specified by the configuration parameter. This validation method is used to ensure consistent naming of elements in the metadata file. For example, the `Extraction Protocol ID` given for an extract in the EXTRACTS

section should match to at least one of the values for `Extraction Protocol ID` given in the `BIOSAMPLE EXTRACTION PROTOCOLS` section.

Configuration Parameter: The name of other metadata field to search for a matching value. Given in the form: *`METADATA SECTION.Field Name`*.

Example Code: `[v]|ir|BIOSAMPLE EXTRACTION PROTOCOLS.Extraction Protocol ID`

Example Valid Value: Any value matching one of the values given for `Extraction Protocol ID` in the `BIOSAMPLE EXTRACTION PROTOCOLS` metadata section.

Experimental File Reference (fileref_exp)

Data Type Code: `fileref_exp`

Validation Description: The value must be the name of a computer file (without any path/directory information) that exists in the data folder of the experiment (not in a subfolder). Case sensitive.

Configuration Parameters: None

Example Code: `[v]|fileref_exp`

Example Valid Value: `calibration_data.cal` would pass validation if the file 'calibration_data.cal' was found in the same experimental data folder as the metadata file being validated.

Instrumental Data File Reference (run_ref)

Data Type Code: `run_ref`

Validation Description: The value must be the name of a NetCDF (*.CDF) file (without the '.CDF' file name extension or any path/directory information) that exists in the data folder of the experiment (not in a subfolder). Case sensitive. A *.PEAKLIST file and an *.XIC file with the same name (except with a '.PEAKLIST' and '.XIC' file name extension, respectively, instead of '.CDF') must also be present in order to pass validation.

Configuration Parameters: None

Example Code: `[v]|run_ref`

Example Valid Value: `mutant1_rep1` would pass validation if the files 'mutant1_rep1.CDF', 'mutant1_rep1.XIC', 'mutant1_rep1.PEAKLIST' were all found in the same experimental data folder as the metadata file being validated.

This user's manual was compiled by Adam J. Carroll.

Copyright 2010
