# Supplementary material for: The MetabolomeExpress Project: enabling web-based processing, analysis and transparent dissemination of GC/MS metabolomics datasets
Source: BMC Bioinformatics. 2010 Jul 14;11:376. doi: 10.1186/1471-2105-11-376 (PMC2912306; doi:10.1186/1471-2105-11-376)
Supplement: Additional file 2 — Notes on FTP repository and data formats. Detailed comments about the MetabolomeExpress FTP repository and data formats supported by MetabolomeExpress and rationales behind their designs. [file 1471-2105-11-376-S2.PDF]

Additional File 2 - Notes on the MetabolomeExpress FTP repository, data and metadata formats

The MetabolomeExpress FTP repository system

For primary data storage, MetabolomeExpress provides registered users each with their own password-protected FTP repository into which data may be uploaded (registration is free for academic use). Data is then accessible for download or analysis via tools provided in the web-interface (described under 'Utility' in the main text). Each FTP repository comes equipped with both public and private folders for storing experimental datasets and GC/MS reference libraries. Data and libraries placed in public folders are available to anonymous users while data and libraries placed in private folders are only accessible to users who have logged in via the web-interface and have permission to access private folders in that repository (permissions may be set by repository owners upon request). Data are organised into discrete 'experiment' folders, each containing all the data associated with a given experiment. Datasets and reference libraries made available in this way are not quality controlled or curated by the MetabolomeExpress team, nor are any minimum reporting standards or dataset completeness policies enforced at this level. However, to take advantage of the data processing and analysis tools of MetabolomeExpress, data must be provided in the supported formats (described briefly below and detailed in additional file 1: the MetabolomeExpress User's Manual). See Figure 1A in the main text for a visual representation of the FTP repository structure.

The FTP mode of data management was chosen over a more complicated HTTP upload/web form-based system because a wide range of FTP client programs are freely available that make it easy for users to upload, download, delete, rename or replace data in their repository as required without the need to build a complicated file management system into the web-interface. The file system (rather than a relational database) was chosen for storage of raw and processed GC/MS data for a number of reasons. Firstly, raw GC/MS datasets are large (typically >>1GB per experiment) and complex in nature (millions of data points per sample and tens to hundreds of samples per experiment) and storage of all these data in a relational database results in unacceptably long query times (data not shown). There is, therefore, little practical advantage in storing these data in a relational database. Secondly, storing entire datasets on the file system greatly simplifies data management since data becomes functionally accessible via the web-interface as soon as it is uploaded, without the need to undertake the additional task of importing all the data into a relational database that can no longer be managed simply by FTP. The added complexity of storing raw and processed data in a relational database would also introduce additional opportunities for bugs to arise and would greatly increase the likelihood that the data management activities of one user could adversely affect the stability of the whole database.

Instead of trying to manage all forms of processed data in the relational database, we use the relational database to store pre-extracted biological information (metabolite response statistics) and effectively ‘index’ relevant parts of the raw data stored in the file system. As discussed in the main text, not all metabolite response statistics in the FTP repository are stored in the main SQL database. Rather, the SQL database is intended only for datasets that meet minimal metadata reporting

and dataset completeness criteria. This approach was chosen to encourage provision of high-quality, transparent data that is described adequately enough and systematically enough (through the use of ontologies wherever possible) to maximise their long-term usefulness.

Raw GC/MS data formats

Raw GC/MS data are currently accepted in the AIA/ANDI-MS/NetCDF open exchange format (*.CDF) since, unlike other open exchange formats such as mzXML [1], mzData [2], mzML [3-4], NetCDF data may be directly exported from virtually every GC/MS instrument manufacturer's proprietary data processing software. The ANDI-MS NetCDF format is indexed by mass spectral scan (the mass spectrum appearing at a particular retention time during an analysis) making retrieval of a particular spectrum very fast. However, extraction of a particular extracted ion chromatogram (EIC, the signal in a particular m/z channel over time) from an ANDI-MS NetCDF file is relatively slow because each and every scan must be searched, in turn, for the relevant signal before the entire EIC may be reconstructed. Therefore, in the interests of enabling rapid display and analysis of EICs, we developed a simple complementary binary raw data format, named XIC (eXtracted Ion Chromatogram, *.XIC), that contains pre-extracted nominal mass EICs indexed by m/z. XIC files are created during the data import process and subsequently enable rapid retrieval of EICs for visualisation or processing while NetCDF files are used for rapid retrieval of spectral scans.

Processed instrument data and metadata formats

Experimental metadata, GC/MS reference libraries and processed GC/MS data (chromatographic peak integration results, library matching reports, data matrices

and statistical/multivariate analysis results) are generated and stored in simple yet extensible tab-delimited text formats with filename extensions indicating their type (for recognition by data-processing modules). We chose simple tab-delimited file structures for these data (as opposed to more complex structures such as XML or highly structured flat-file formats) to make compliance easier for data contributors who prefer to use their own data processing pipelines and also to ensure that the processed data available from MetabolomeExpress is readily importable by the general community into as many third-party software packages as possible without the need for specialist XML-handling skills or flat file parsing scripts. These file structures are detailed in the MetabolomeExpress User's Manual (additional file 1). The metadata exchange format was designed around structures described in ArMet [5] and recommendations of the Metabolomics Standards Initiative [6].

Metadata validation and controlled vocabularies

To address minimal metadata reporting and ontological requirements specific to different research fields, it is necessary to develop a slightly different validation template for each field. We have developed a simple syntax for quickly defining new metadata templates. This syntax defines field names and their associated valid data types (eg. text with minimum word count, email, URL, integer etc.) and controlled vocabularies. Full details of how to interpret metadata templates defined using the current syntax are provided in the MetabolomeExpress User's Guide (additional file 1). MetabolomeExpress currently provides 18 different metadata templates for major model organisms and research fields. These, are available from the Help tab of the MetabolomeExpress web interface. These templates should be viewed as 'beta' versions and may evolve as consensus on reporting standards is reached for

different research fields. MetabolomeExpress provides a tool to construct functional 'starting' metadata files (with only fields required for data processing filled out) from very simple tables containing the minimal sample information required for data processing. Empty fields may then be completed when it is convenient or necessary for public dissemination (via the MetabolomeExpress database of metabolite response statistics, for example).

References

1. Pedrioli PG, Eng JK, Hubley R, Vogelzang M, Deutsch EW, Raught B, Pratt B, Nilsson E, Angeletti RH, Apweiler R *et al*: **A common open representation of mass spectrometry data and its application to proteomics research**. *Nat Biotechnol* 2004, **22**(11):1459-1466.

2. Orchard S, Montechi-Palazzi L, Deutsch EW, Binz PA, Jones AR, Paton N, Pizarro A, Creasy DM, Wojcik J, Hermjakob H: **Five years of progress in the Standardization of Proteomics Data 4th Annual Spring Workshop of the HUPO-Proteomics Standards Initiative April 23-25, 2007 Ecole Nationale Superieure (ENS), Lyon, France**. *Proteomics* 2007, **7**(19):3436-3440.

3. Deutsch EW: **Mass spectrometer output file format mzML**. *Methods Mol Biol* 2010, **604**:319-331.

4. Deutsch E: **mzML: a single, unifying data format for mass spectrometer output**. *Proteomics* 2008, **8**(14):2776-2777.

5. Jenkins H, Hardy N, Beckmann M, Draper J, Smith AR, Taylor J, Fiehn O, Goodacre R, Bino RJ, Hall R *et al*: **A proposed framework for the description of plant metabolomics experiments and their results**. *Nat Biotechnol* 2004, **22**(12):1601-1606.

6. Fiehn O, Wohlgemuth G, Scholz M, Kind T, Lee do Y, Lu Y, Moon S, Nikolau B: **Quality control for plant metabolomics: reporting MSI-compliant studies**. *Plant J* 2008, **53**(4):691-704.
